# Twisted One-Dimensional Charge Transfer and Related Y-Shaped Chromophores with a 4*H*-Pyranylidene Donor: Synthesis and Optical Properties

**DOI:** 10.1021/acs.joc.0c02438

**Published:** 2021-02-02

**Authors:** Víctor Tejeda-Orusco, Raquel Andreu, Jesús Orduna, Belén Villacampa, Santiago Franco, Alba Civera

**Affiliations:** † Instituto de Nanociencia y Materiales de Aragón (INMA)-Departamento de Química Orgánica, CSIC-Universidad de Zaragoza, Zaragoza 50009, Spain; ‡ Instituto de Nanociencia y Materiales de Aragón (INMA)-Departamento de Física de la Materia Condensada, CSIC-Universidad de Zaragoza, Zaragoza 50009, Spain

## Abstract

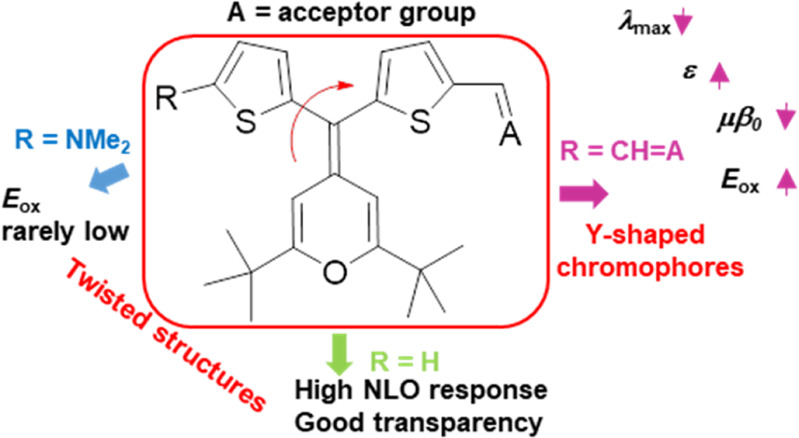

Three series of push–pull derivatives bearing 4*H*-pyranylidene as electron donor group and a variety of acceptors were designed. On one hand, one-dimensional chromophores with a thiophene ring (series **1H**) or 5-dimethylaminothiophene moiety (series **1N**) as an auxiliary donor, non-coplanar with the π-conjugated system, were synthesized. On the other hand, related two-dimensional (2D) Y-shaped chromophores (series **2**) were also prepared to compare how the diverse architectures affect the electrochemical, linear, and second-order nonlinear optical (NLO) properties. The presence of the 5-dimethylaminothiophene moiety in the exocyclic C=C bond of the pyranylidene unit gives rise to oxidation potentials rarely low, and the protonation (with an excess of trifluoroacetic acid) of its derivatives results in the apparition of a new blue-shifted band in the UV–visible spectra. The analysis of the properties of derivatives with and without the additional thiophene ring shows that this auxiliary donor leads to a higher NLO response, accompanied by an enhanced transparency. Y-shaped chromophores of series **2** present a blue-shifted absorption, higher molar extinction coefficients, and higher *E*_ox_ values compared to their linear twisted counterparts. As concerns NLO properties, 2D Y-shaped architecture gives rise to somewhat lower μβ values (except for thiobarbiturate derivatives).

## Introduction

Second-order nonlinear optical (NLO) materials^1^ based on organic molecules have been investigated for long time due to their potential applications^2^ related with second harmonic generation (SHG), optical switching, sensing,^3^ electro-optical modulation,^4^ and so on. Microscopic nonlinearities have been dramatically improved over time, and push–pull dipolar chromophores, with a donor−π–acceptor (D−π–A) structure and a suitable intramolecular charge transfer (ICT),^5^ have reached very high hyperpolarizability (β) values. The extent of the ICT can be delicately tuned by varying the components of this kind of molecules (D, π, and A), demonstrating to be essential to maximize the second-order NLO activity.^5^ Certainly, most applications require to transfer the molecular nonlinearity to the macroscopic level, looking for bulk materials with large NLO activity.^1a,6^

The “construction” of new chromophores remains an interesting research topic. Focusing on the molecular design, two strategies have been used in recent times. On one hand, multidimensional charge-transfer chromophores have been developed as a way to balance the nonlinearity-transparency trade-off arising from the strong push–pull structure and to tune the ICT. Extraordinary arrangements of D–A chromophores that may be pictured as uppercase letters (molecules with shape similar to H, L, T, V, X, and Y) appeared in the literature within the last few years.^7^ Furthermore, twisted ICT chromophores^8^ exhibit high hyperpolarizability compared to planar D–A molecules, showing to be a promising strategy for improving the microscopic nonlinearity of chromophores. Besides, these geometries hinder dipole aggregation at the macroscopic level.

Concerning donor units, the proaromatic character^9^ of the pyranylidene moiety, and the subsequent gain in aromaticity along the ICT, has turned this fragment into a versatile building block, widening their use not only in the field of NLO^9,10^ but also in different material research areas such as dye-sensitized solar cells (DSSCs),^11^ organic light-emitting diodes,^12^ organic photovoltaics (OPV),^13^ or hole-transporting materials for perovskite solar cells^14^ because of the special electron-donating ability and chemical stability of this moiety.^15^

**Chart 1:**
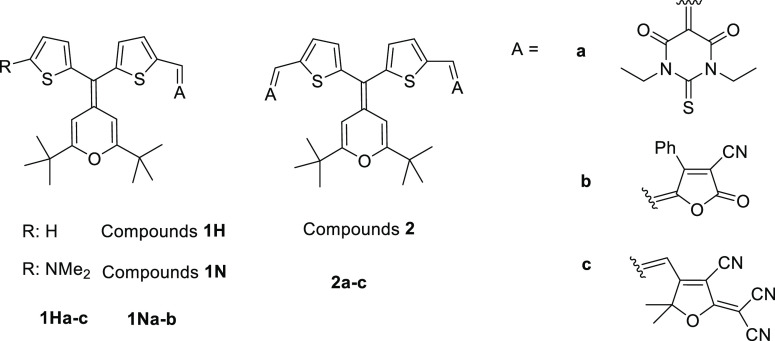
Molecular Structures of the Target Compounds; Synthesis, Cyclic Voltammetry (CV), and UV Studies in CH_2_Cl_2_ for Compounds **1Hb** and **2b** in Ref (13b).

Having in mind the abovementioned two approaches (multidimensional charge-transfer and twisted chromophores) and taking advantage of our consolidated experience in the synthesis of D−π–A systems with the 4*H*-pyranylidene as the donor moiety,^9,10a,10b,10e,11b,15^ we submit the design, synthesis, and characterization of three series of molecules (Chart 1) for their study as second-order NLO chromophores. We have included in this analysis compounds **1Hb** and **2b**, two derivatives previously studied^13b^ as small donor molecules for OPV (see below).

As acceptor units, two common strong organic electron-withdrawing fragments such as a thiobarbituric acid (derivatives **a**) and 2-dicyanomethylene-3-cyano-4,5,5-trimethyl-2,5-dihydrofuran (TCF) (derivatives **c**) were used, together with 4-phenyl-2-oxo-2,5-dihydrofuran-3-carbonitrile (derivatives **b**), a moderate acceptor that has given rise to an excellent matching of effective polarized chromophores with upgraded second-order NLO activity.^10e^

For the chromophores **1H** and **1N**, the exocyclic C=C double bond of the 4*H*-pyranylidene donor is equipped with a thiophene (**1H**) or a 5-dimethylaminothiophene (**1N**) unit. These substituents have the following three features: (i) they are necessarily not coplanar with the D–A system; (ii) they could be considered as auxiliary donors;^16^ and (iii) they introduce steric hindrance. As a result, they can improve the electron-donating ability of the 4*H*-pyranylidene moiety, also showing an isolating effect^17^ that hinders the antiparallel dipole–dipole orientations. Derivatives **2** can be considered as Y-shaped chromophores with the thiophene ring as the π-spacer and the withdrawing group arranged around the central 4*H*-pyranylidene core. Both types of chromophores (**1H**/**1N** and **2**) summarize the two strategies explained above: to have a multidimensional ICT and twisted structures.

Related compounds to those proposed in Chart 1 have been previously reported by our group for other two different applications, and the influence of the auxiliary thiophene ring has been explored: (i) dyes designed for DSSCs analogous to **1H** compounds, with cyanoacrylic acid as an acceptor, gave rise to twisted structures preventing the formation of aggregates and, therefore, increasing the efficiency of the derived cells;^11d^ (ii) compound **1Hb** and a related system with dicyanovinyl as an electron-withdrawing end were studied as donor materials for OPV, leading to poor efficiencies due to the fact that the twisted structure prevents the formation of suitable π–π intermolecular interactions.^13b^ In this paper, we will study how this architecture affects the second NLO activity.

Electrochemical, linear, and NLO properties of systems in Chart 1 were carefully studied both experimentally and theoretically and, eventually, compared to those of a planar conjugated analogue.

## Results and Discussion

### Synthesis

**Scheme 1:**
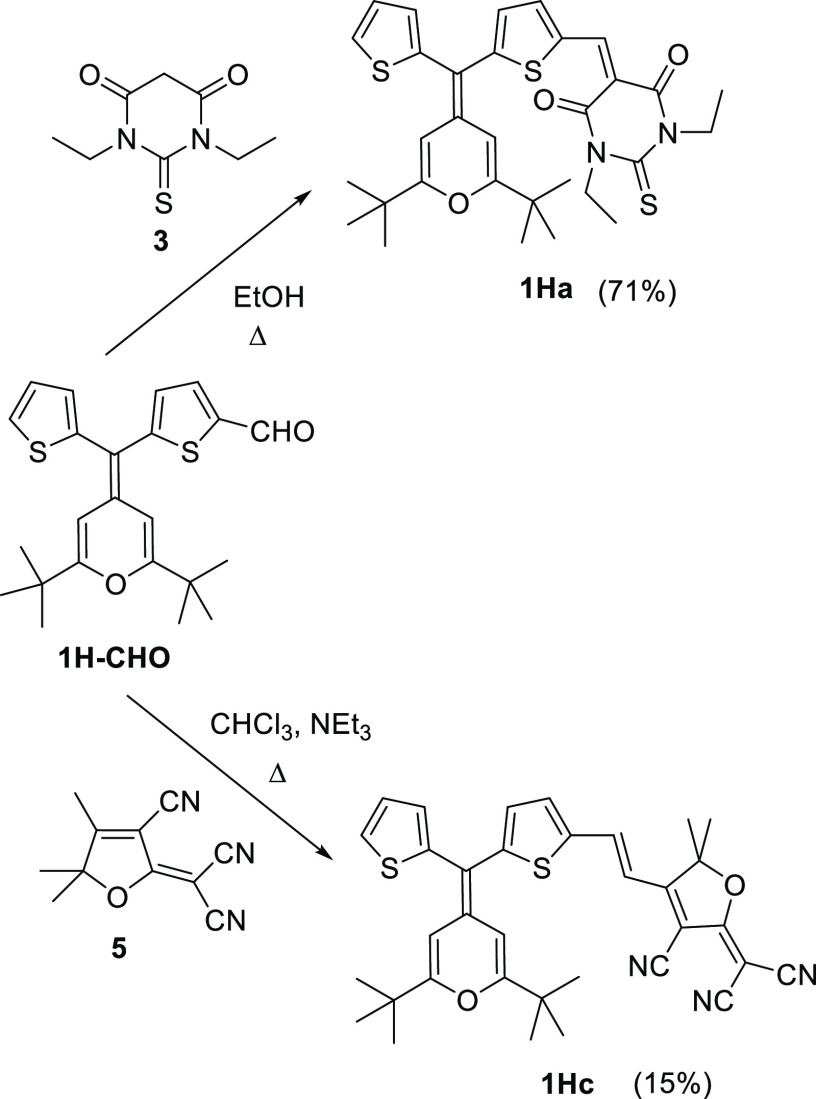
Preparation of 4*H*-Pyranylidene-Based Push–Pull Molecules **1Ha**,**c**

**Scheme 2:**
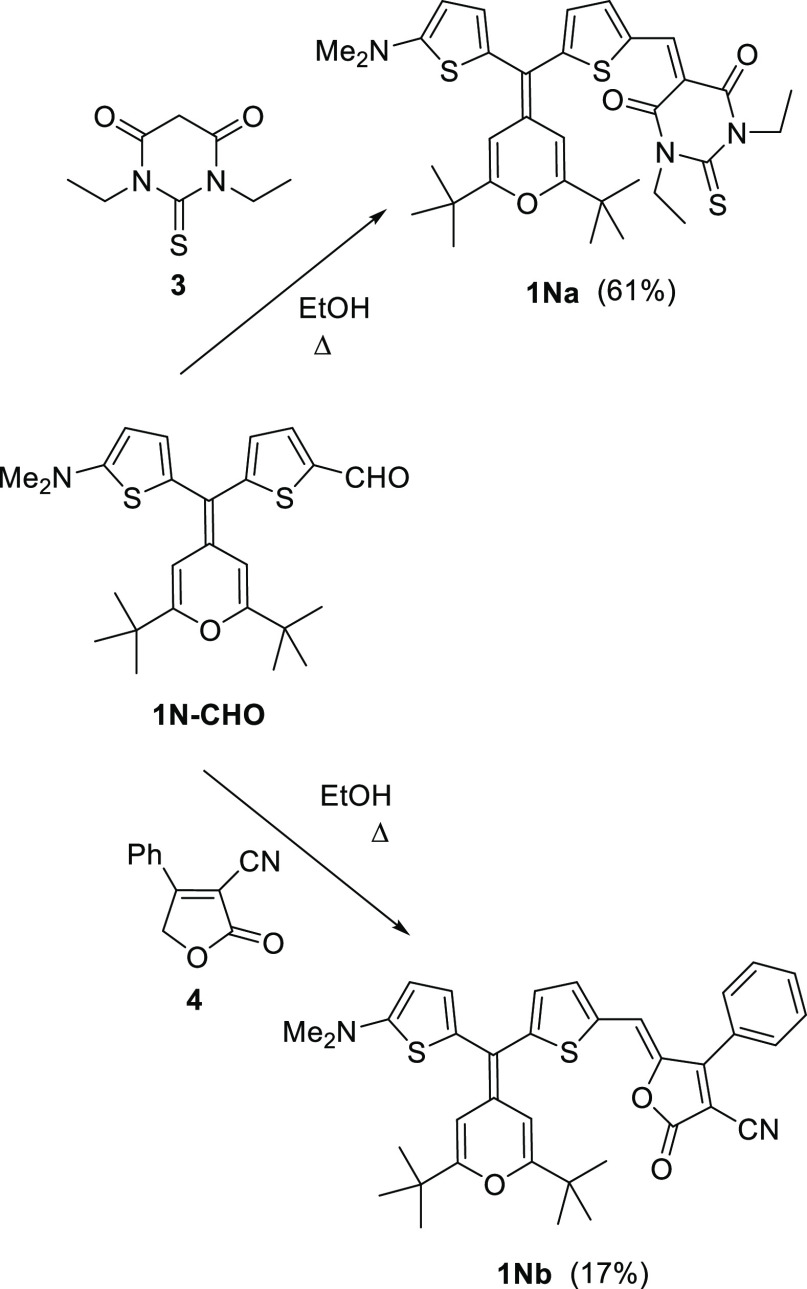
Preparation of 4*H*-Pyranylidene-Based Push–Pull Molecules **1Na**,**b**

**Scheme 3:**
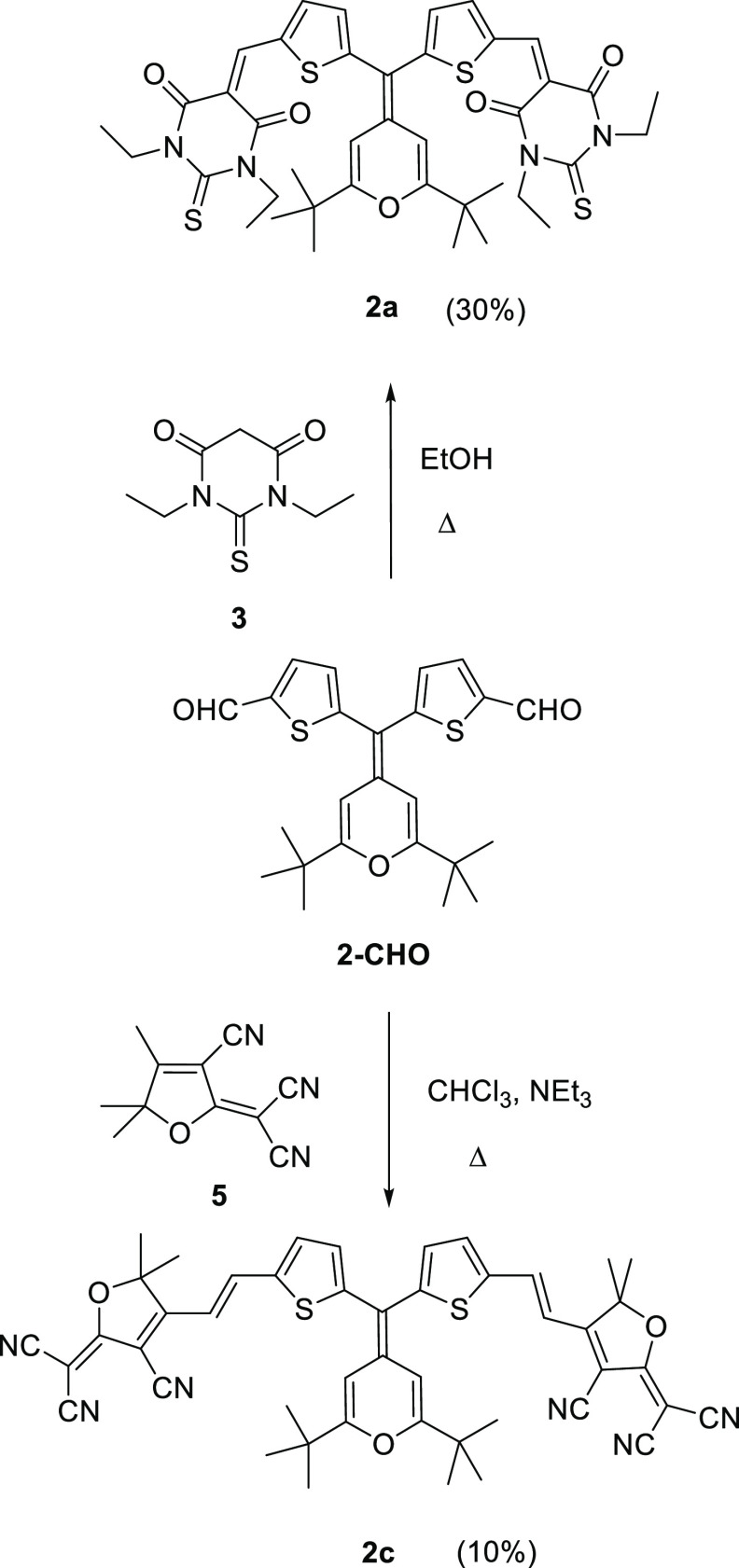
Synthesis of Y-Shaped Chromophores **2a**,**c**

**Chart 2:**
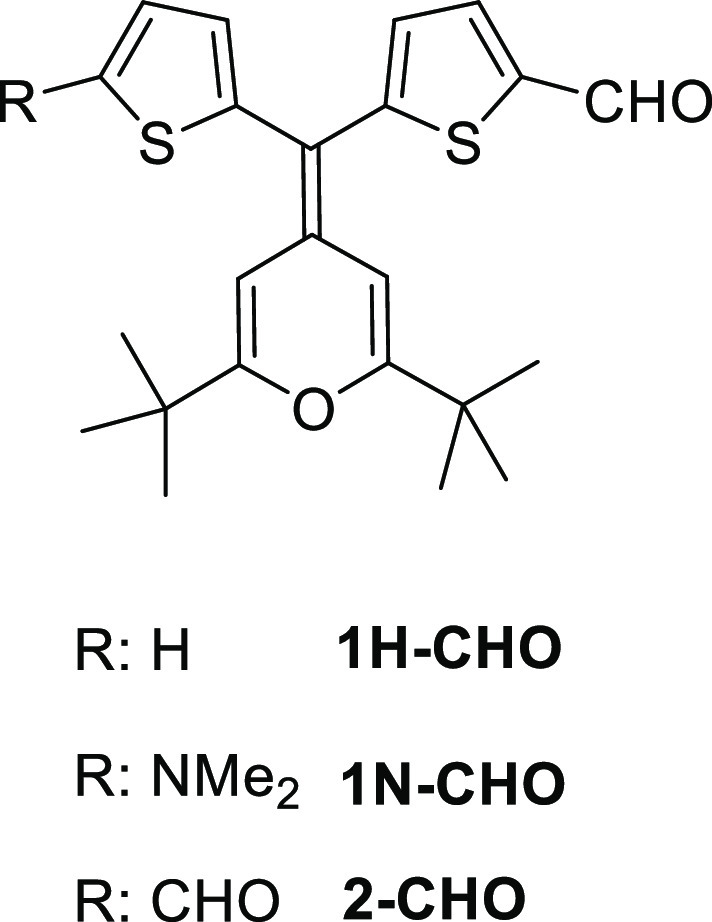
Precursor Aldehydes; For Their Synthesis, See Ref (11d).

Synthesis of compounds **1Ha**,**c**, **1Na**,**b**, and **2a**,**c** is shown in Schemes 1–3, respectively, with the previously reported 4*H*-pyranylidene-containing aldehydes **1H–CHO**, **1N-CHO**, and **2-CHO** (Chart 2)^11d^ as precursors.

Chromophores were synthesized by the Knoevenagel reaction between the aldehyde and the corresponding acceptors: (1,3-diethyl-2-thiobarbituric acid) (**3**), 4-phenyl-2-oxo-2,5-dihydrofuran-3-carbonitrile (**4**),^18^ and TCF (**5**).^19^ The reaction conditions (base and solvent) have been adapted to the nature of the electron-withdrawing moiety.^20^ Unfortunately, the TCF derivative **1Nc** could not be isolated.

Purification of compounds **2a**,**c** required one step more compared to their analogues **1Ha**,**c** in order to separate traces of the mono-condensation products.

The new chromophores were characterized by infrared (IR) and ^1^H and ^13^C NMR spectroscopies and mass spectrometry (see Experimental Section). The analysis of the ^3^*J*_HH_ coupling constants allows us to infer that the CH= CH bond in compound **c** has an *E* configuration.

### Calculated Structures

The molecular geometry and electronic structure of the titled chromophores have been studied by means of density functional theory (DFT) calculations. The conductor-like polarizable continuum model (CPCM) solvation method was used, choosing CH_2_Cl_2_ as the solvent.

**Figure 1:**
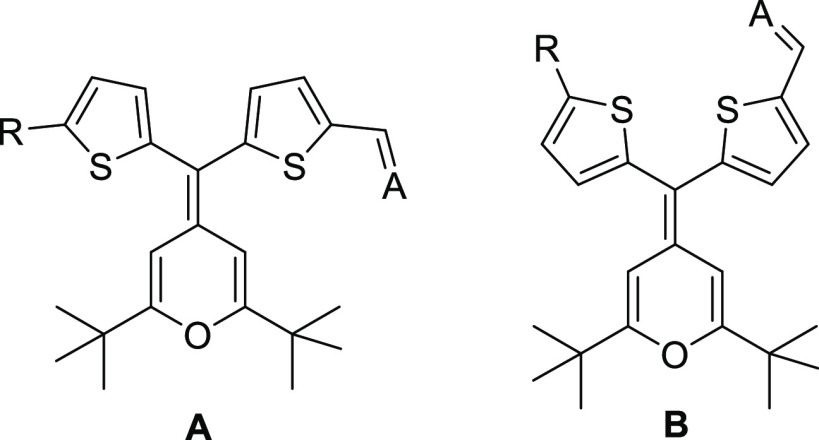
Conformations used in geometry optimizations.

We have considered two possible conformations (**A** and **B** in Figure 1) for geometry optimizations.

Calculations resulted in conformation **A** being more stable for compounds **1H** and **1Na**, while conformation **B** is more stable than **A** for compounds **2** and **1Nb**. Energy differences between conformers were however below 1 kcal/mol with the exception of **1Hb** (4.89 kcal/mol) and **2b** (3.14 kcal/mol). Most molecular properties have been calculated using the most stable conformation, while NLO properties were calculated (see the Nonlinear Optical Properties section) on conformation **B**. The reason is that since this has a larger dipole moment than **A**, it is expected to be favored by the large electric field used for the electric field-induced SHG (EFISHG) measurements.

The molecular geometry of these compounds results from the distortion caused by steric hindrance between pyrane and thiophene rings. For compounds **1H** and **1Na**, the thiophene spacer and the acceptor moiety are rotated approximately 15° with respect to the pyranylidene donor, thus allowing a good donor–acceptor conjugation. Contrary to this, the auxiliary donor thiophene ring is rotated by *ca.* 75° with respect to the pyrane ring and therefore does not interact with the donor–acceptor system (Figure S26). This distortion from planarity is in accordance with that found in the related compounds recently reported as dyes for DSSC^11d^ or donor materials for OPV.^13b^

Conformation **B** imposes a somewhat different geometry for **1Nb**, leading to a more distorted donor–thiophene–acceptor system with the thiophene spacer rotated 35° with respect to the pyranylidene unit and the auxiliary thiophene donor rotated 57°.

Compounds **2**, having two identical acceptor groups, arrange in a *C*_2_ symmetry with both thiophene rings rotated 40–45° with respect to the pyrane ring.

Bond lengths reflect the existence of two predominant resonance forms (Figure S27): the neutral one and a zwitterionic form with the aromatized pyrylium donor and a quinoid thiophene spacer.

For compounds **1H** and **1N**, all the C–C bond lengths in the thiophene spacer equal to 1.39–1.40 Å, distance between the expected lengths for single and double C–C bonds, which reflects a similar contribution of both resonance forms. Contrary to this, the auxiliary thiophene shows clearly differentiated single (1.43 Å) and double (1.36–1.38 Å) C–C bonds.

The two acceptor chromophores **2**, having two equivalents thiophene rings, display a less marked quinoid character with single C–C bonds of 1.40–1.41 Å and double C=C bonds of 1.38–1.39 Å.

**Figure 2:**
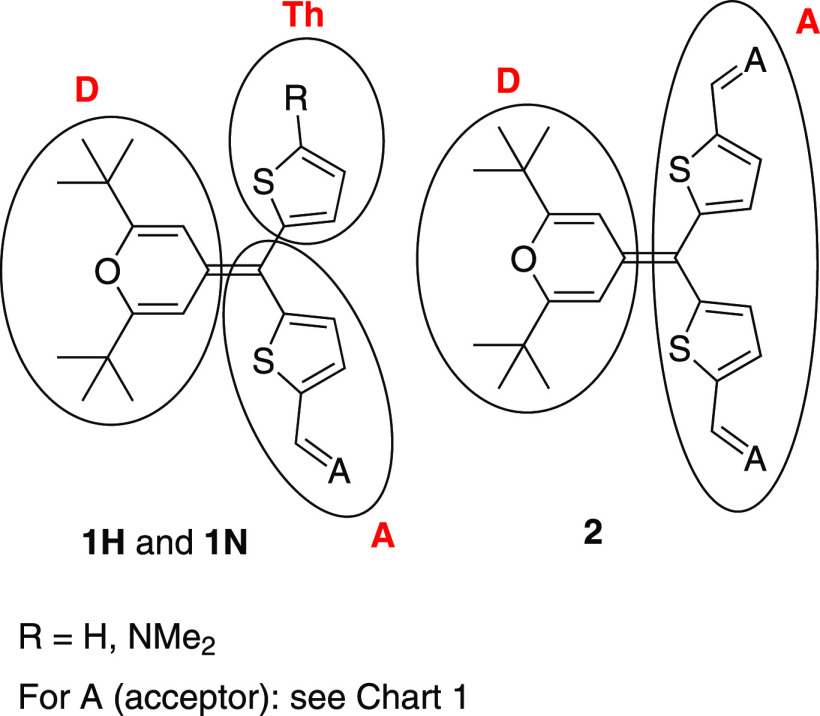
Molecular domains for title compounds.

The contribution of these two resonance forms is also denoted by the natural bond orbital (NBO) charge analysis (Table 1 together with Figure 2 for notation of molecular domains). While the pyranylidene donor supports a positive charge ranging from +0.259 to +0.352, and the thiophene spacer and acceptor support an equivalent negative charge, the auxiliary thiophene donor (denoted as Th) in compounds **1H** and **1N** remains nearly uncharged (−0.007 to +0.002).

**Table 1:** Calculated NBO Charges (CPCM-M06-2x/6-31G*) in CH_2_Cl_2_ on Different Molecular Domains (See Figure 2 for Notation)

compound	D	A	Th
**1Ha**	+0.352	–0.345	–0.007
**1Na**	+0.350	–0.350	0.000
**2a**	+0.351	–0.351	
**1Hb**	+0.299	–0.291	–0.008
**1Nb**	+0.259	–0.261	+0.002
**2b**	+0.296	–0.296	
**1Hc**	+0.339	–0.335	–0.004
**2c**	+0.338	–0.338	

Quite surprisingly, the charge on the pyrane ring of compounds **2** is nearly identical to that of their analogues **1H**, indicating that each acceptor group in **2** supports half the charge of their counterparts **1H** and that the charge on the donor is related to the nature of the acceptor group rather than on the number of acceptors.

### Electrochemical Study

The electrochemical characterization of the chromophores has been performed by CV in CH_2_Cl_2_ solution using Bu_4_NPF_6_ as the supporting electrolyte. Data are presented in Table 2, along with calculated highest occupied molecular orbital (HOMO) and lowest unoccupied molecular orbital (LUMO) energies and first oxidation potentials, showing a fairly good agreement to experimental values. We included data for compound **6b**, analogue to **1Hb** lacking the thiophene unit, whose synthesis and comparative study are explained in the NLO Properties section.

**Table 2:** Electrochemical Data^a^ and *E*_ox_, *E*_HOMO_, and *E*_LUMO_ Values Theoretically Calculated^b^

compound	*E*_ox1_^1/2^ (V)	*E*_ox2_^1/2^ (V)	*E*_red_ (V)	*E*_ox_ calc^c^ (V)	*E*_HOMO_ (eV)	*E*_LUMO_ (eV)
**1Ha**	0.71	1.00	–0.92	0.67	–6.42	–2.39
**1Na**	0.29^d^		–1.01	0.19	–6.27	–2.37
**2a**	0.77	1.09	–1.00	0.75	–6.43	–2.43
**1Hb**	0.64^e^	0.92^e^	–0.87^e^	0.54	–6.25^e^	–2.56^e^
**1Nb**	0.19	0.30	–0.91	0.13	–6.03	–2.50
**2b**	0.68^e^	0.95^e^	–0.82^e^	0.63	–6.30^e^	–2.58^e^
**1Hc**	0.65	0.92	–0.73	0.63	–6.36	–2.71
**2c**	0.74	0.99	–0.70	0.77	–6.46	–2.77
**6b**^f^	0.66		–0.92			

a 5 × 10^–4^ M in CH_2_Cl_2_*vs* Ag/AgCl (3 M KCl), glassy carbon working electrode, Pt counter electrode, 20 °C, 0.1 M NBu_4_PF_6_, 100 mV s^–1^ scan rate. For these conditions: *E*_ox_^1/2^ ferrocene = +0.45 V.

b Calculated at the CPCM-M06-2x/6-311+G(2d,p)//CPCM-M06-2x/6-31G* level in CH_2_Cl_2_.

c Referenced to Ag/AgCl.

d This wave corresponds to two oxidation processes. See interpretation in the text.

e Data taken from ref (13b).

f See the NLO section for synthesis and discussion of the properties.

All voltammograms show three redox processes corresponding to one irreversible reduction peak (implicating the acceptor end) and two reversible oxidation steps, related to the two one-electron oxidations of the pyranylidene unit, as previously described for other chromophores with a similar design^13b^ and D−π–A platinum complexes with this donor end.^21^

There is a liaison between the structure of these systems and their electrochemical behavior, being the singular oxidation behavior of derivatives **1Na**,**b** the most remarkable.

**Figure 3:**
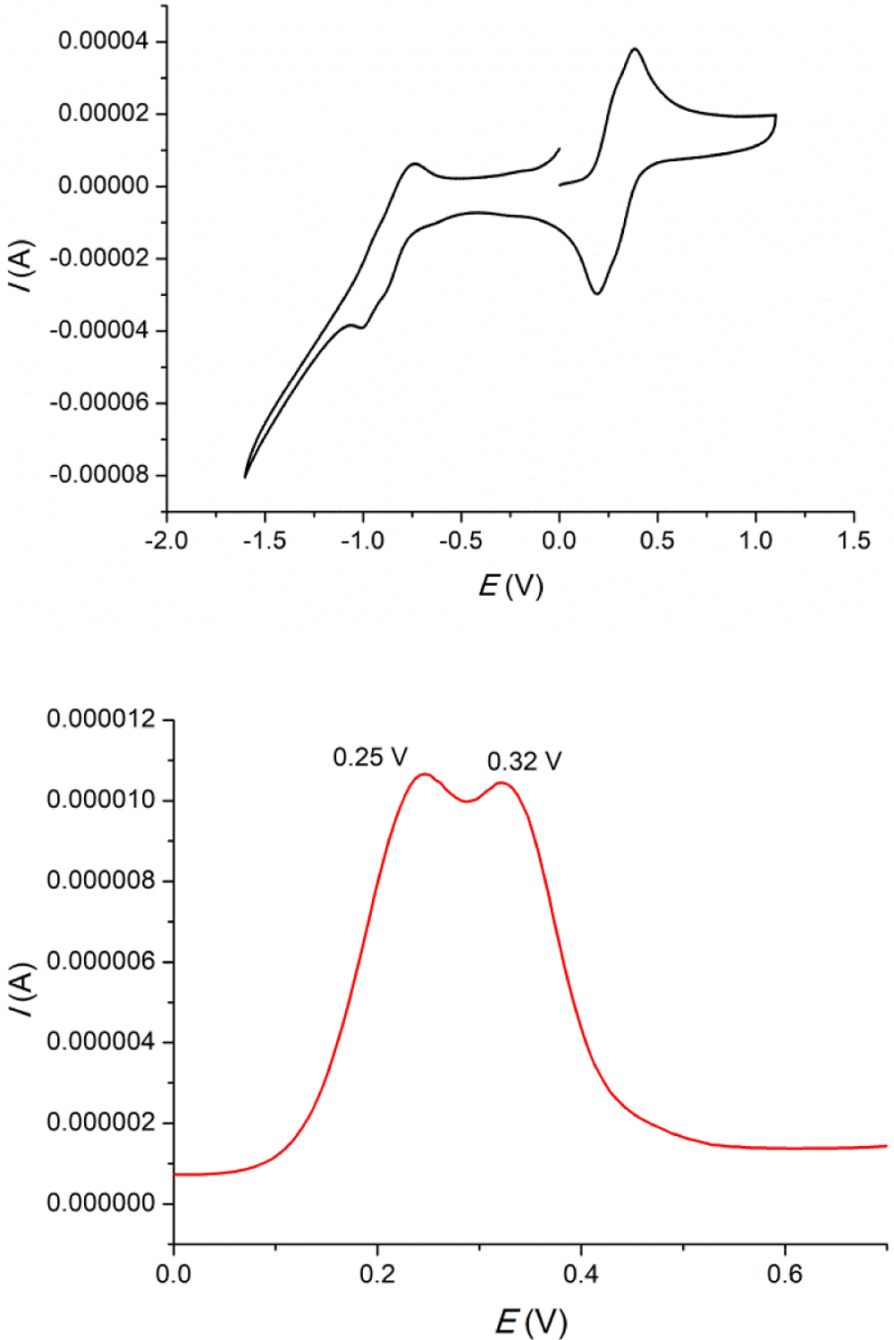
Voltammograms of compound **N1a**: CV (top) and DPV (bottom).

The oxidation potential values for **1Na**,**b** are extremely low for 4*H*-pyranylidene derivatives;^9,10e^ these compounds are easily oxidized, so the cation radical and the dication generated are very stable species. The two oxidation waves for **1Na**,**b** are very close to each other, to the point of appearing practically together at 0.29 V with a “shoulder” in the case of **1Na** (Figure 3-top). Therefore, this compound was alternatively measured using a more sensitive electrochemical technique (differential pulse voltammetry, DPV), which allowed the two expected oxidation peaks to be resolved (+0.25 and +0.32 V, respectively) (Figure 3, bottom). Precursor aldehyde **1N-CHO** (Chart 2) has a similar behavior, with two close oxidations processes at 0.19 and 0.34 V (no wave reduction was found).

Hence, comparing compounds **1Na**,**b** with their analogues **1Ha**,**b** lacking the dimethylamino group, a considerable decrease of the oxidation half-potentials is observed, whereas the reduction potential is slightly increased; both facts agree with the presence of an excellent donor substituent. Evaluation of calculated *E*_HOMO_ (*E*_LUMO_) data shows that systems **1N** have higher (only slightly higher) values than the corresponding **1H** analogues. Nevertheless, differences in HOMO energies are not large enough to rationalize the large decrease in oxidation potentials caused by the introduction of the dimethylamino group. We must consider that oxidation potentials arise from energy differences between the oxidized radical cations and neutral species and the large decrease in the oxidation potential caused by the dimethylamino fragment is mainly due to the stabilization of the oxidized radical cation provided by this functional group.^11d^

On the other hand, within each series (**1H**, **1N**, **2**) and focusing on the acceptor unit, |*E*_red_| decreases in the order **a** > **b** > **c**, corroborating the superior electron-withdrawing strength of the TCF unit, and points to a superior electron-withdrawing ability for furanone **4** than that could be expected. This trend is in agreement with computational calculations: *E*_LUMO_ decrease in the order **a** > **b** > **c**. Regarding *E*_ox_ values, both *E*_ox1_ and *E*_ox2_ decrease when passing from chromophores with the thiobarbiturate group (**a**) to their analogues **b**,**c**. In the case of Y-shaped compounds, system **2b** presents the lowest *E*_ox_ value.

Compounds **1H** and **1N**, with one acceptor moiety, show less anodic potentials than the corresponding analogues **2**; this result can be assigned to the higher planarization of the π-conjugated system in mono-functionalized compounds **1H** and **1N**, leading to an enhanced stabilization of the radical cation.^13b^

Eventually, the impact of this structural variation on |*E*_red_| values depends on the acceptor unit: for **a** series, with the thiobarbituric acid as the electron-withdrawing end, the lowest |*E*_red_| is found for compound **1Ha**, whereas for derivatives **b–c**, chromophores **2b** and **2c** show easier reduction processes.

### Optical Properties

UV–vis absorption data for the titled chromophores are gathered in Table 3. Different solvents with varied polarities have been used in the study. We included data for compound **6b**, analogue to **1Hb** lacking the thiophene unit, whose synthesis and comparative study is explained in the NLO Properties section.

**Table 3:** UV–Vis Absorption Data^a^

compound	λ_abs_ 1,4-dioxane	ε 1,4-dioxane	λ_abs_ CH_2_Cl_2_	ε CH_2_Cl_2_	λ_abs_ DMF	ε DMF
**1Ha**	611	43,417	635	56,896	620	40,720
**1Na**	622	40,453	646	41,284	635	32,790
**2a**	603	53,816	627	56,220	617	b
**1Hb**	603	30,205	645^c^	38,406^c^	613	31,197
**1Nb**	629	16,410	671	14,428	639	15,062
**2b**	592	34,628	626^c^	40,131^c^	597	35,355
**1Hc**	625	24,569	707	31,152	652	19,163
**2c**	594	29,464	664	31,975	648	21,224
**6b**^d^			662	33,940	645	30,516

a All λ_abs_ data in nanometer; the unit for ε is M^–1^ cm^–1^.

b Determination not possible due to the low solubility of the compound.

c Data taken from ref (13b).

d See the NLO section for synthesis and discussion of the properties.

Broad and intense bands located in the visible region can be observed for all compounds. These bands are related with an ICT process between the donor and the acceptor fragments (spectra are shown in Figures S14–S23).

Concerning the presence of the dimethylamino group in the thiophene ring (comparison between compounds **1Ha–b** and **1Na–b**), a bathochromic shift of the ICT is encountered for systems **1N**. In contrast, the molar extinction coefficient (ε) decreases for the three solvents studied, being particularly important for furanone derivatives **b**, with a factor decrease of 2. Therefore, the dimethylamino substituent implies an absorption at higher wavelengths and a decrease in the ability to absorb the light.

As regards the effect of the acceptor end, it can be observed that variation of λ_max_ depends on the structure of the chromophore. For **1H** series, λ_max_ decreases in the order **c** > **b**,**a**. In the case of **1N** compounds, a red shift of the maximum absorption wavelength is observed for **1Nb** when compared to its analogue **1Na** for the three studied solvents. This feature confirms, as it has been mentioned in the Electrochemical Study section, that furanone **4** could be read as an acceptor end stronger than the thiobarbituric moiety.

In contrast, solvent is a factor to keep in mind for chromophores **2**: for the less polar 1,4-dioxane, **2a** presents the highest λ_max_, while for the more polar dimethylformamide (DMF) is the TCF derivative **2c**, the compound with the largest λ_max_ value. Moreover, thiobarbiturate systems **a** present larger ε values than their **b**,**c** counterparts according to other donor−π–thiobarbituric derivatives previously reported.^9^

The presence of 1D or 2D ICT, triggered by the presence of one or two acceptor units, has a significant influence on the electronic absorption properties of the studied systems. Thus, compounds **2** show blue-shifted absorptions and higher molar extinction coefficients when compared to their analogues **1H**. This latter feature is in agreement with previous results on other 2D chromophores including a 4*H*-pyranylidene moiety.^10d,22^

Data in Table 3 show for all compounds positive solvatochromism when comparing 1,4-dioxane and CH_2_Cl_2_, which becomes negative on going from CH_2_Cl_2_ to DMF. This variety of behavior is the same as found for other D−π–A compounds,^23^ including some 4*H*-pyranylidene derivatives.^15,20a^ A ground state with an enhanced contribution of the charge-separated resonance structure could be favored by increasing the polarity of the solvent^23c^ (CH_2_Cl_2_ to DMF) and could become greater than that in the excited state giving rise to a hypsochromic effect.

The UV–vis spectra of the new chromophores have been also studied using time-dependent DFT (TD-DFT) calculations. The calculations were performed in dichloromethane using a CPCM solvation model, and both **A** and **B** conformations (see Figure 1) were considered since they are supposed to co-exist in solution at room temperature. These results are gathered in Table 4.

**Table 4:** TD-DFT-Calculated (CPCM-M06-2x/6-311+G(2d,p)//CPCM-M06-2x/6-31G*) Absorption Wavelengths and Oscillator Strengths (*f*) in Dichloromethane

	conformation A		conformation B	
**compound**	λ (nm)	*f*	λ (nm)	*f*
**1Ha**	579	1.34	560	1.59
**1Na**	594	1.28	583	1.42
**2a**	575	1.68	553	0.66
	552	0.41	531	1.61
**1Hb**	623	1.20	610	1.71
**1Nb**	641	1.16	612	1.26
**2b**	611	1.69	571	0.64
	583	0.21	550	1.53
**1Hc**	651	1.58	616	1.82
**2c**	633	2.19	593	0.80
	595	0.26	563	1.96

**Figure 4:**
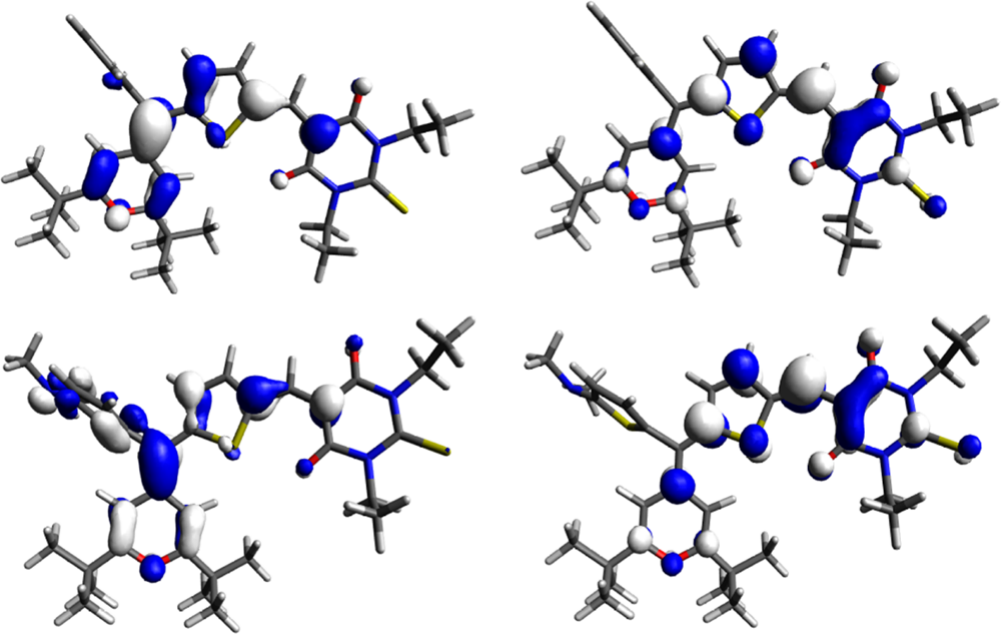
0.04 contour plots of the HOMO (left) and LUMO (right) of compounds **1Ha** (top) and **1Na** (bottom).

The calculations underestimate the lower absorption wavelengths by 15–56 nm, denoting errors below 0.2 eV in excitation energies that are reasonable for this kind of calculations.^24^ For compounds **1H** and **1N**, the excitation of a HOMO electron into the LUMO is the main contribution to the lowest energy transition (see Figure 4).

Although the HOMO and LUMO are mainly located on the donor and on the acceptor, respectively, both frontier orbitals extend over the thiophene spacer. The large HOMO–LUMO overlap results in a large oscillator strength (*f*) and therefore a large ε.

Comparing **1H** and **1N** series, it can be seen (Figure 4) that the dimethylamino group increases the contribution of the auxiliary thiophene to the HOMO but not to the LUMO. The energy of the HOMO is therefore higher for compounds **1N** than that for **1H**, while the energy of LUMO is nearly identical (see Table 2), thus a reduced HOMO–LUMO gap and consequently a bathochromic shift for **1N** with respect to **1H** is observed (see Table 3). Given that the auxiliary thiophene in compounds **1N** contributes to the HOMO but not to the LUMO, a reduced HOMO–LUMO overlap is encountered, causing lower *f* and ε values compared to **1H**.

Considering the effect of the acceptor group, its electron-withdrawing strength causes a stabilization of the LUMO that results in lower HOMO–LUMO gaps and larger absorption wavelengths following this order **c** > **b** > **a**, in agreement with experimental results.

**Figure 5:**
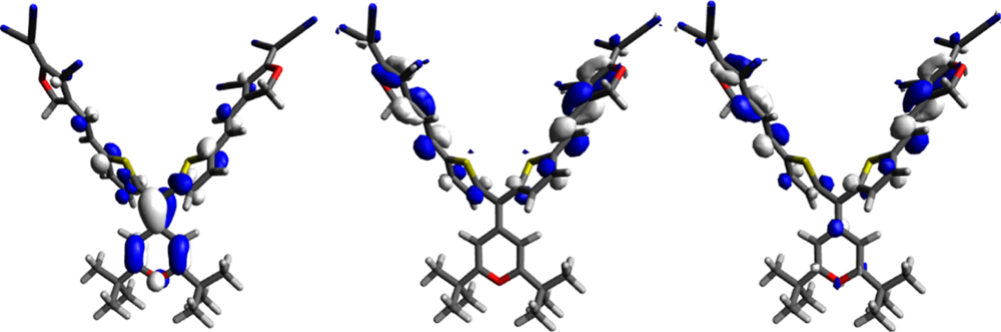
0.04 contour plots of the HOMO (left), LUMO (center), and LUMO + 1 (right) of compound **2c**.

The presence of two acceptor groups (compounds **2**) results in two unoccupied orbitals (LUMO and LUMO + 1) (Figure 5) with similar energy arising from the combination of the orbitals of each acceptor moiety. This aspect provides two electronic transitions (HOMO → LUMO and HOMO → LUMO + 1), close in energy, and probably overlap in the absorption spectrum, resulting in large observed ε values.

For chromophores **1Na**,**b** bearing a dimethylamino group, the effect of its protonation in CH_2_Cl_2_ solution was studied by titration with trifluoroacetic acid (TFA) (10^–2^ M) and registration of the corresponding absorption spectra. In order to have a good control of the titration process, compound **1Hb**, lacking the dimethylamino group, was also studied (Figure S24).

**Figure 6:**
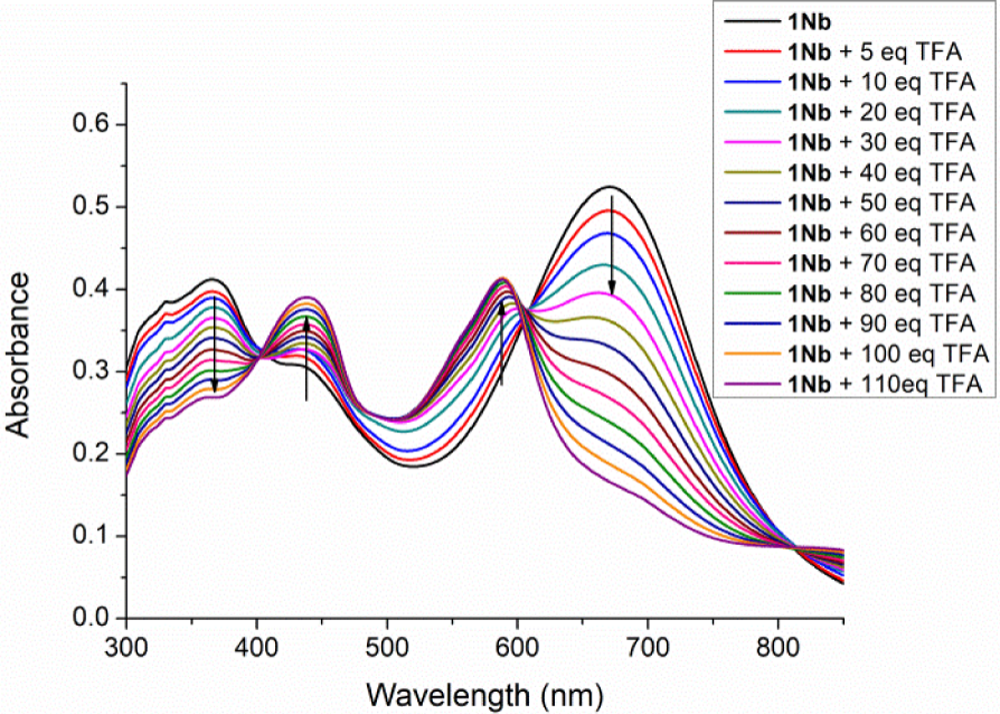
Absorption spectra of a CH_2_Cl_2_ solution of compound **1Nb** (*c* = 3 × 10^–5^ M) upon addition of TFA (5–110 equiv).

In the case of compound **N1b**, the changes observed in its UV–vis spectra upon the addition of this acid are illustrated in Figure 6.

The progressive attenuation of the charge-transfer absorption band for the neutral compound (centered at 671 nm) is encountered on increasing the concentration of acid, and a new higher-energy band corresponding to the protonated species appeared (λ_max_ = 588 nm). The difference of 83 nm accounts for the acceptor character of the protonated dimethylamino group.

On the other hand, the band in **1Nb** associated to transitions π–π* (366 nm) decreases with protonation, with the appearance of a new red-shifted band (438 nm).

A significant TFA concentration (5 equiv) was needed before changes in the absorption spectra were remarked. Two isosbestic points were observed at 401 and 605 nm.

Comparing with the titration of compound **1Hb** (Figure S24), the formation of other species apart the chromophore **1Hb** is not observed, validating the protonation of the dimethylamino group, with any sign of the protonation of other moieties in the chromophore.

Compound **1Na** (see Figure S25) followed similar trends, although a sharper decrease of the ICT absorption was encountered and the disappearance of the band at 646 nm occurs with 40 equiv of TFA.

### Nonlinear Optical Properties

In order to evaluate the second-order nonlinear response of the compounds, EFISHG measurements were performed at 1907 nm in dichloromethane. A simple two-level model^25^ was used to obtain the dispersion-corrected μβ_0_ values from the experimental μβ (Table 5). The second harmonic wavelength is not overlapped with any of the absorption bands of the studied compounds.

**Table 5:** Experimental and Calculated NLO Properties

compound	μβ^a^ (10^–48^ esu)	μβ_0_^b^ (10^–48^ esu)	μβ^c^ (10^–48^ esu)	μβ_0_^d^ (10^–48^ esu)	μ^e^ (debye)	β_0_^e^ (10^–30^ esu)	μβ_0_^e^ (10^–48^ esu)
**1Ha**	1260	625			12.0	78	841
**1Na**	1900	910			12.2	83	950
**2a**	1400	710			15.1	52	778
**1Hb**	2600	1250	1200	630	13.3	198	1795
**1Nb**	2050	910	1280	625	11.4	161	1278
**2b**	2350	1190	1050	575	13.4	139	1857
**1Hc**	5500	2140			21.5	211	4068
**2c**	3900	1770			30.4	140	4244
**6b**^f^	2200	1000	1050	505			

a μβ values determined in CH_2_Cl_2_ at 1907 nm (experimental uncertainty less than ±15%).

b Experimental μβ_0_ values in CH_2_Cl_2_ extrapolated using the two-level model.

c μβ values determined in DMF at 1907 nm (experimental uncertainty less than ±20%).

d Experimental μβ_0_ values in DMF calculated using the two-level model.

e Calculated at the HF/6-31G*//CPCM-M06-2x/6-31G* level.

f See below for synthesis and discussion of the properties.

The benchmark chromophore Disperse Red 1 has been measured under the same experimental conditions. μβ_0_ values of 510 × 10^–48^ and 444 × 10^–48^ esu in CH_2_Cl_2_ and DMF, respectively, have been obtained.

Molecular hyperpolarizabilities (β) and dipole moments (μ) have also been estimated by quantum chemical calculations. Having in mind that DFT methods usually fail to determine NLO properties,^26^ calculations have been performed using the Hartree–Fock (HF) method. While theoretical results overestimate the experimental values, they reproduce the observed trends.

With respect to the influence of the electron-withdrawing end on the NLO properties of the studied chromophores, for series **1H** and **2**, the nonlinearities increase in the order **a** < **b** < **c**, which again indicates the higher acceptor ability of the TCF unit. Modification of the acceptor is more significant for **1H** systems than for Y-chromophores **2**. The increasing trend when acceptor changes from **a** to **c** is also reproduced by theoretical calculations. Considering a two-level approach,^25a,27^ hyperpolarizability depends on the transition dipole moment (μ_01_) or the oscillator strength (*f*), the dipole moment change on excitation (Δμ_01_), and the excitation energy (*E*_01_). The change in the molecular hyperpolarizability may be mostly due to the decreased excitation energies along the series **a** > **b** > **c**.

On the other hand, compounds **1Na–b** show essentially the same NLO response. Theoretical calculations show that while the hyperpolarizability (β_0_) of **1Nb** is more than double that of **1Na**, the dipole moment is better aligned to the β_0_ vector in **1Na** (22°) than that in **1Nb** (46°), thus resulting in scarce differences in the dot product μβ_0._

The effect of the dimethylamino group in the NLO properties depends on the acceptor unit. Thus, for thiobarbituric derivatives **a**, going from **1H** chromophore to **1N**, one implies an increase on the NLO response [μβ_0_ (**1Na**)/μβ_0_ (**1Ha**) = 1.46]. Nonetheless, for furanone derivatives **b**, a slight decrease in the μβ_0_ value is observed [μβ_0_ (**1Nb**)/μβ_0_ (**1Hb**) = 0.73].

Theoretical calculations also show that the effect of the dimethylamino fragment on hyperpolarizability depends on the acceptor. Paying attention to the parameters involved in the two-level approach, this substituent causes a decreased excitation energy (*E*_01_) accompanied by a decreased oscillator strength (*f*) and an increased dipole moment change (Δμ_01_). The relative weight to these opposed factors determines the final increased or decreased hyperpolarizability.

Y-compounds **2** (except **2a** with a thiobarbituric acid acceptor) show lower experimental μβ_0_ values than those of their analogues **1H**. This result can be considered a bit surprising since the opposite trend has been observed in other chromophores with the pyran ring incorporated into the π-spacer,^22a,22b,28^ acting as the donor in D–A–D compounds^10a,10d^ or in A–D–A systems.^21c^ While theoretical calculations do not reproduce exact trends in μβ_0_ values on passing from compounds **1H** to **2**, the calculated values for chromophores **2** are similar to their **1H** analogues. The calculations reveal that the effect of the increased dipole moment on passing from **1H** to **2** is opposed by decreased hyperpolarizability, and therefore, the resulting μβ_0_ depends on the relative effect of these parameters.

**Scheme 4:**
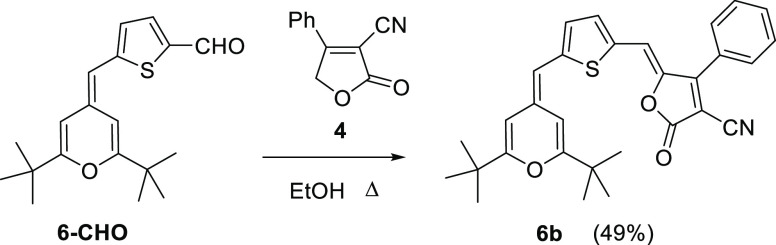
Synthesis of Compound **6b**

In order to study the effect of the additional thiophene ring, nearly orthogonal to the extended π-system, on the final properties, compound **6b**, analogue to derivative **1Hb** lacking this unit, was prepared (Scheme 4). This compound was synthesized from the previously reported aldehyde **6-CHO**(29) and acceptor **4**. The electrochemical and optical properties of this new chromophore are gathered in Tables 2, 3, and 5.

Comparison of compounds **1Hb** and **6b** shows that **6b**, lacking the thiophene ring, features (i) a red shift in λ_abs_^13b^ together with a decrease for the ε value; (ii) a single irreversible anodic peak corresponding to the formation of the pyrylium radical cation and subsequent dimerization process, as described for other methylene pyran derivatives^30^ together with a slightly more cathodic *E*_red_ value; and (iii) a decrease in the NLO response. Hence, it is noteworthy that chromophore **1Hb**, with the auxiliary thiophene ring while being more transparent, shows a higher NLO activity [μβ_0_ (**1Hb**)/μβ_0_ (**6b**) = 1.25].

Calculations on **6b** predict a bathochromic shift with respect to **1Hb** (635 *vs* 623 nm on the more stable conformation **A**) but a nearly identical NLO behavour with μβ_0_ = 1794 × 10^–48^ esu (on the more polar conformation **B**).

In order to study the influence of solvent in the NLO activity, measurements in DMF were performed for furanone-containing derivatives **b**. These measurements show some limitations: it is not a usual solvent and its EFISHG parameters are not accurately calibrated. For these reasons, μβ values (10^–48^ esu) given in Table 5 present a wider margin of error (20%), although the reproducibility is similar to that observed in CH_2_Cl_2_.

The lower μβ_0_ values obtained in DMF indicate that **b** chromophores are more polarized in this solvent than in dichloromethane (the zwitterionic form, as shown in Figure S27, has a more important presence than in CH_2_Cl_2_) and that these systems are left-handed chromophores in Marder’s plot^31^ (A/B region), with the neutral form predominating in this solvent polarity range.

## Conclusions

Three series of compounds featuring a 4*H*-pyranylidene moiety have been designed and studied based on two current approaches for the optimization of NLO properties: twisted chromophores (series **1H** and **1N**) and multidimensional charge transfer (series **2**).

Y-arranged chromophores **2** show lower absorption wavelengths, higher *E*_ox_ values, and lower μβ_0_ values than their 1D analogues **1H**. Thiobarbituric acid derivatives represent an exception to the trend observed in NLO response.

The incorporation of 5-dimethylaminothiophene moiety into the exocyclic C= C bond of the pyranylidene unit leads to derivatives **1N** to be easily oxidized. The effect of this no coplanar moiety in the NLO response depends on the acceptor unit.

Twisted chromophore **1Hb**, with the thiophene ring in the exocyclic position, shows higher NLO activity and wider transparency range than the analogue **6b**, lacking this moiety. These results point out that this design is suitable to achieve structures with high second-order NLO responses, allowing an isolating effect needed for preparing organic electro-optic devices.

## Experimental Section

### General Experimental Methods

IR measurements were carried out in KBr using a Fourier transform IR spectrometer. Melting points were obtained in open capillaries and are uncorrected. ^1^H-NMR spectra were recorded at 300 or 400 MHz. ^13^C-NMR spectra were recorded at 100 MHz, respectively; δ values are given in parts per million (relative to tetramethylsilane) and *J* values in Hertz. The apparent resonance multiplicity is described as s (singlet), d (doublet), t (triplet), q (quartet), and m (multiplet). Electrospray mass spectra were recorded on a Q-ToF spectrometer; accurate mass measurements were achieved using sodium formate as an external reference.

CV measurements were performed using a glassy carbon working electrode, a Pt counter electrode, and a Ag/AgCl reference electrode. The experiments were carried out under argon in CH_2_Cl_2_, with Bu_4_NPF_6_ as the supporting electrolyte (0.1 mol L^–1^). Step potential was 0.01 V, and the interval time was 0.5 s.

EFISHG measurements have been carried out using an excitation wavelength of 1907 nm. This fundamental radiation is the output of a H_2_ Raman shifter pumped by a Q-switched Nd:YAG laser at 1064 nm. The laser repetition rate is 10 Hz, and the pulse width is 8 ns. A computer-controlled NLO spectrometer completes the SHG experimental setup. The excitation beam is split into two; the less intense one is directed to a *N*-(4-nitrophenyl)-(l)-prolinol (NPP) powder sample whose SHG signal is used as a reference in order to reduce the effects of laser fluctuations. The second one is passed through a linear (vertical) polarizer and focused into the EFISHG wedge-shaped liquid cell. Voltage pulses of 5 kV and 3 μs are applied across the cell (2 mm gap between the electrodes) synchronously with the laser pulses. The harmonic signals from both the EFISHG cell and the NPP reference are measured with two photomultipliers. Interference filters are used to remove the residual excitation light beyond the sample and the reference.

The molecular μβ values of the reported compounds have been determined in dichloromethane and DMF (for **b** derivatives). Several solutions of concentration in the range 1.5 × 10^–3^ to 5 × 10^–4^ M were measured. μβ_0_ values were extrapolated using a two-level dispersion model^25a^ and λ_max_ corresponding to the lowest energy band. Under the same experimental conditions, μβ_0_ deduced for DR1 in dichloromethane was 510 × 10^–48^ esu, quite close to the value reported in the same solvent by Dirk *et al.*(32) For DMF, the deduced value was 444 × 10^–48^ esu.

DFT calculations were performed using Gaussian 16^33^ with the ultrafine integration grid. Solvent effects were estimated using a CPCM.^34,35^ Equilibrium geometries were optimized using the M06-2x hybrid meta-GGA exchange correlation functional^36^ and the medium size 6-31G* base.^37^ Optimized geometries were characterized as minima by frequency calculations. Excitation energies were calculated by time-dependent single-point calculations using the M06-2x/6-311G(2d,p) model chemistry. Absorption spectra were estimated through the calculation of vertical excitations at the ground-state geometry. Ground-state oxidation potentials (*E*_ox_) were calculated using the M06-2x/6-311-G(2d,p) energies and calculating the thermal corrections to Gibbs free energy at the M06-2x/6-31G* level. Molecular orbital contour plots were obtained using the Avogadro software^38^ at an 0.04 isosurface value.

Aldehydes **1H-CHO**,^11d^**1N-CHO**,^11d^**2-CHO**,^11d^ and **6-CHO**(29) and acceptors **4**(18) and **5**(19) were prepared, as previously described.

#### 5-(5-((2,6-Di-*tert*-butyl-4*H*-pyran-4-ylidene)(thiophen-2-yl)methyl)thiophen-2-yl)methylene-1,3-diethyl-2-tioxodihydropyrimidine-4,6(1*H*,5*H*)-dione (**1Ha**)

A mixture of aldehyde **1H–CHO** (50 mg, 0.125 mmol) and 1,3-diethyl-2-thiobarbituric acid (**3**) (28 mg, 0.14 mmol) in absolute ethanol (3 mL) was refluxed in a heating block under argon with exclusion of light for 7 h [thin-layer chromatography (TLC) monitoring]. After cooling, the resulting solid was isolated by filtration and washed with cold pentane. A green solid was obtained (51.6 mg; 71%).

mp (°C) 226–228. IR (KBr) ν: (cm^–1^) 2973 (C sp^3^–H), 1647 (C=O), 1551, 1530 and 1501 (C=C Ar.), 1393 (C=S). ^1^H NMR (CD_2_Cl_2_, 300 MHz): δ 8.48 (s, 1H), 7.73 (d, *J* = 4.5 Hz, 1H), 7.41 (dd, *J*_1_ = 5.2 Hz, *J*_2_ = 1.2 Hz, 1H), 7.13–7.10 (m, 2H), 6.95–6.93 (m, 2H), 5.92 (d, *J* = 2.2 Hz, 1H), 4.57–4.49 (m, 4H), 1.31 (s, 9H), 1.28–1.22 (m, 6H), 1.13 (s, 9H). ^13^C{^1^H} NMR (CD_2_Cl_2_, 100 MHz): δ 179.4, 168.9, 167.5, 165.8, 161.9, 160.4, 148.0, 147.2, 142.8, 138.9, 135.1, 129.0, 128.3, 127.9, 126.9, 108.8, 107.7, 104,4, 102.9, 44.1, 43.4, 36.8, 36.4, 28.2, 28.1, 12.7. HRMS (ESI^+^/Q-TOF) *m*/*z*: [M]^+•^ calcd for C_31_H_36_N_2_O_3_S_3_, 580.1883; found, 580.1862.

#### (*E*)-2-(3-Cyano-4-(2-(5-((2,6-di-*tert*-butyl-4*H*-pyran-4-ylidene)(thiophen-2-yl)methyl)thiophen-2-yl)vinyl)-5,5-dimethylfuran-2(5*H*)-yliden)malononitrile (**1Hc**)

Triethylamine (41 μL, 0.30 mmol) was added to a solution of **1H–CHO** (119.4 mg, 0.30 mmol) and acceptor TCF (**5**; 66.3 mg, 0.33 mmol) in CHCl_3_ (6 mL) under an argon atmosphere, and the mixture was refluxed in a heating block for 5 days (TLC monitoring). Then, the solvent was evaporated and the crude product was purified by flash chromatography (silica gel) using hexane/AcOEt 8:2 as the eluent to afford a dark blue solid (26 mg; 15%).

mp (°C) 278–279 (dec.). IR (KBr) ν: (cm^–1^) 2926 (C sp^3^–H), 2219 (C≡ N), 1658 (C=C), 1595, 1558 and 1533 (C=C Ar).

^1^H NMR (CD_2_Cl_2_, 400 MHz): δ 7.78 (d, *J* = 15.5 Hz, 1H), 7.42–7.41 (m, 2H), 7.10 (dd, *J*_1_ = 5.2 Hz, *J*_2_ = 3.5 Hz, 1H), 6.97 (d, *J* = 4.4 Hz, 1H), 6.94 (dd, *J*_1_ = 3.5 Hz, *J*_2_ = 1.2 Hz, 1H), 6.75 (d, *J* = 2.1 Hz, 1H), 6.48 (d, *J* = 15.5 Hz, 1H), 5.91 (d, *J* = 2.1 Hz, 1H), 1.71 (s, 6H), 1.28 (s, 9H), 1.14 (s, 9H). ^13^C{^1^H} NMR (CD_2_Cl_2_,100 MHz): δ 173.7, 168.1, 166.9, 158.0, 143.3, 140.0, 137.8, 137.5, 137.1, 128.9, 127.9, 126.8, 113.2, 112.6, 112.0, 111.2, 107.9, 103.7, 101.9, 97.6, 67.1, 36.6, 36.3, 28.2, 28.1, 26.9. HRMS (ESI^+^/Q-TOF) *m*/*z*: [M + Na]^+^ calcd for C_34_H_33_N_3_NaO_2_S_2_, 602.1906; found, 602.1932.

#### 5-5′-((((2,6-Di-*tert*-butyl-4*H*-pyran-4-ylidene)methylene)bis(thiophene-2,5-diyl))bis(methanylylidene))bis(1,3-diethyl-2-thioxodihydropyrimidine-4,6(1*H*,5*H*)-dione) (**2a**)

A mixture of aldehyde **2-CHO** (80 mg, 0.19 mmol) and 1,3-diethyl-2-thiobarbituric acid (**3**) (80 mg; 0.40 mmol) in absolute ethanol (5 mL) was refluxed in a heating block under argon with exclusion of light for 24 h (TLC monitoring). After cooling, the resulting solid was isolated by filtration, washed with cold pentane, and finally purified by flash chromatography (silica gel) with CH_2_Cl_2_ as the eluent. A dark blue solid was obtained (47 mg; 30%).

mp (°C) 290–291 (dec.). IR (KBr) ν: (cm^–1^) 3072 (C sp^2^–H), 2980 (C sp^3^–H), 1684 (C=O), 1655 (C=C), 1547 and 1515 (C=C Ar.), 1395 (C=S). ^1^H NMR (CD_2_Cl_2_, 400 MHz): δ 8.62 (s, 2H), 7.88 (d, *J* = 4.2 Hz, 2H), 7.13 (d, *J* = 4.2 Hz, 2H), 6.62 (s, 2H), 4.58–4.51 (m, 8H), 1.30–1.25 (m, 30H). ^13^C{^1^H} NMR (CD_2_Cl_2_, 100 MHz): δ 179.4, 169.1, 162.8, 161.6, 160.3, 148.9, 147.2, 139.7, 136.9, 129.9, 109.9, 108.1, 103.8, 44.3, 43.6, 36.7, 30.3, 28.1, 12.7. HRMS (ESI^+^/Q-TOF) *m*/*z*: [M + Na]^+^ calcd for C_40_H_46_N_4_NaO_5_S_4_, 813.2243; found, 813.2228.

#### (*E*,*E*)-2,2′-(((2,6-Di-*tert*-butyl-4*H*-pyran-4-ylidene)methylene)bis(thiophene-2,5-diyl))bis(ethene-1,2-diyl))bis(3-cyano-5,5-dimethylfuran-4(5*H*)-yl-2(5*H*)-ylidene)dimalononitrile (**2c**)

Triethylamine (68 μL, 0.50 mmol) was added to a solution of **2-CHO** (80 mg, 0.19 mmol) and acceptor TCF (**5**; 100 mg, 0.50 mmol) in CHCl_3_ (6 mL) under an argon atmosphere, and the mixture was refluxed in a heating block for 3 days (TLC monitoring). Then, the solvent was evaporated and the crude product was purified by flash chromatography (silica gel) using hexane/AcOEt 8:2 as the eluent. A further purification by flash chromatography (silica gel) with CH_2_Cl_2_/AcOEt 9.7:0.3 was needed. Finally, the resulting solid was washed with cold pentane to afford compound **2c** as a dark blue solid (15 mg; 10%).

mp (°C) 172–173. IR (KBr) ν: (cm^–1^) 2963 (C sp^3^–H), 2224 (C≡N), 1659 (C=C), 1577 (C=C Ar.). ^1^H NMR (CD_2_Cl_2_, 400 MHz): δ 7.72 (d, *J* = 15.8 Hz, 2H), 7.45 (d, *J* = 4.0 Hz, 2H), 7.04 (d, *J* = 4.0 Hz, 2H), 6.60 (d, *J* = 15.8 Hz, 2H), 6.39 (s, 2H), 1.75 (s, 12H), 1.22 (s, 18H). ^13^C{^1^H} NMR (CD_2_Cl_2_, 100 MHz): δ 173.9, 168.5, 154.4, 139.7, 139.6, 138.2, 137.0, 130.0, 113.0, 112.8, 112.2, 111.5, 102.9, 97.9, 36.6, 31.2, 28.1, 26.9. HRMS (ESI^+^/Q-TOF) *m*/*z*: [M + Na]^+^ calcd for C_46_H_40_N_6_NaO_3_S_2_, 811.2496; found 811.2470.

#### 5-((5-((2,6-Di-*tert*-butyl-4*H*-pyran-4-ylidene) (5-(dimethylamino)thiophen-2-yl)methyl)thiophen-2-yl)methylene)-1,3-diethyl-2-thioxodihydropyrimidine-4,6(1*H*,5*H*)-dione (**1Na**)

This compound was prepared by following the same procedure as for **1Ha**, starting from **1N–CHO** (54 mg, 0.12 mmol) with a reaction time of 5 h. A dark blue solid was obtained (47 mg; 61%).

mp (°C) 176–180. IR (KBr) ν: (cm^–1^) 2965 and 2868 (C sp^3^–H), 1653 (C=O), 1537 (C=C, Ar.), 1382 (C=S). ^1^H NMR (CD_2_Cl_2_, 400 MHz): δ 8.49 (s, 1H), 7.78 (d, *J* = 4.4 Hz, 1H), 7.12 (d, *J* = 4.4 Hz, 1H), 6.98 (d, *J* = 2.1 Hz, 1H), 6.62 (d, *J* = 3.7 Hz, 1H), 6.17 (d, *J* = 2.1 Hz, 1H), 5.85 (d, *J* = 3.7 Hz, 1H), 4.58–4.52 (m, 4H), 2.93 (s, 6H), 1.30–1.25 (m, 15H), 1.19 (s, 9 H). ^13^C{^1^H} NMR (CD_2_Cl_2_, 100 MHz): δ 179.4, 167.4, 161.9, 160.4, 147.9, 147.5, 135.3, 129.1, 128.2, 126.5, 44.1, 43.4, 31.2, 28.2,12.8. HRMS (ESI^+^/Q-TOF) *m*/*z*: [M]^+•^ calcd for C_33_H_41_N_3_O_3_S_3_, 623.2305; found, 623.2315; *m*/*z*: [M + Na]^+^ calcd for C_33_H_41_N_3_NaO_3_S_3_, 646.2202; found, 646.2184.

#### 5-((5-((2,6-Di-*tert*-butyl-4*H*-pyran-4-ylidene) (5-(dimethylamino)thiophen-2-yl)methyl)thiophen-2-yl)methylene)-2-oxo-4-phenyl-2,5-dihydrofuran-3-carbonitrile (**1Nb**)

To a solution of aldehyde **1N–CHO** (66 mg, 0.15 mmol) in absolute ethanol (4 mL), acceptor **4** (30.5 mg; 0.16 mmol) was added. The mixture was refluxed in a heating block under argon with exclusion of light for 48 h (TLC monitoring). After cooling, the resulting solid was isolated by filtration and washed with cold pentane. Finally, filtration through a plug of silica gel with hexane/AcOEt 9.8:0.2 as the eluent afforded a dark green solid (16 mg, 17%).

mp (°C) 217–219. IR (KBr) ν: (cm^–1^) 2923 (C sp^3^–H), 2224 (C≡N), 1749 (C=O), 1659 (C=C), 1604 and 1543 (C=C, Ar.). ^1^H NMR (CD_2_Cl_2_, 400 MHz): δ 7.64–7.59 (m, 5H), 7.42 (d, *J* = 4.3 Hz, 1H), 7.02 (d, *J* = 4.3 Hz, 1H), 6.73 (s, 1H), 6.62 (d, *J* = 2.1 Hz, 1H), 6.61 (d, *J* = 3.8 Hz, 1H), 6.12 (d, *J* = 2.1 Hz, 1H), 5.81 (d, *J* = 3.8 Hz, 1H), 2.92 (s, 6H), 1.24 (s, 9H), 1.17 (s, 9H). ^13^C{^1^H} NMR: not registered due to its low solubility. HRMS (ESI^+^/Q-TOF) *m*/*z*: [M]^+•^ calcd for C_36_H_36_N_2_O_3_S_2_, 608.2162; found, 608.2169.

#### 5-((5-((2,6-Di-*tert*-butyl-4*H*-pyran-4-ylidene)methyl)thiophen-2-yl)methylene)-2-oxo-4-phenyl-2,5-dihydrofuran-3-carbonitrile (**6b**)

A solution of aldehyde **6-CHO** (173.3 mg, 0.55 mmol) and acceptor **4** (104.3 mg, 0.56 mmol) in EtOH (10 mL) was refluxed in a heating block for 24 h under an argon atmosphere (TLC monitoring), and then the solvent was evaporated under reduced pressure. The crude was purified by flash chromatography (silica gel) using hexane/AcOEt 9:1 as the eluent to obtain a blue solid. (131.3 mg, 49%).

mp (°C) 79 (dec.). IR (KBr) ν: (cm^–1^) 3062 (C sp^2^–H), 2964 (C sp^3^–H), 2219 (C≡N), 1751 (C=O), 1659, 1601 and 1538 (C=C, Ar.). ^1^H NMR (CD_2_Cl_2_, 400 MHz): δ 7.64–7.61 (m, 5 H), 7.45 (d, *J* = 4.3 Hz, 1 H), 6.91 (d, *J* = 4.3 Hz, 1 H), 6.76 (s, 1 H), 6.61 (d, *J* = 1.9 Hz, 1 H), 5.98 (s, 1 H), 5.88 (d, *J* = 1.9 Hz, 1 H), 1.32 (s, 9 H),1.24 (s, 9 H). ^13^C{^1^H} NMR (CD_2_Cl_2_, 100 MHz): δ 167.5, 164.7, 164.3, 159.6, 154.7, 141.4, 136.4, 134.8, 132.0, 131.2, 128.9, 128.4, 127.9, 126.0, 115.1, 112.6, 105.3, 104.5, 100.0, 35.7, 35.1, 27.2 (×2). HRMS (ESI^+^/Q-TOF) *m*/*z*: [M + Na]^+^ calcd for C_30_H_29_NNaO_3_S, 506.1760; found, 506.1761.

## Supporting Information Available

General experimental methods, NMR and UV–vis spectra of new compounds, NLO measurements and computed energies, and Cartesian coordinates of optimized geometries of all chromophores (PDF)

## Dedication

Dedicated to the memory of Dr. Thi-Thao Nguyen-Pipaud, good friend and warm-hearted colleague.
