# Supplementary material for: Twisted One-Dimensional Charge Transfer and Related Y-Shaped Chromophores with a 4H-Pyranylidene Donor: Synthesis and Optical Properties
Source: J Org Chem. 2021 Feb 2;86(4):3152–63. doi: 10.1021/acs.joc.0c02438 (PMC9161450; doi:10.1021/acs.joc.0c02438)
Supplement: Supplementary file 1 — jo0c02438_si_001.pdf [file jo0c02438_si_001.pdf]

Twisted one-dimensional charge transfer and related Y-shaped chromophores with a 4*H*-pyranylidene donor: synthesis and optical properties

Víctor Tejeda-Orusco,[a] Raquel Andreu,[a,*] Jesús Orduna,[a] Belén Villacampa,[b] Santiago Franco,[a] Alba Civera[a].

[a] Instituto de Nanociencia y Materiales de Aragón (INMA)-Departamento de Química Orgánica, CSIC-Universidad de Zaragoza, Zaragoza 50009, Spain.

[b] Instituto de Nanociencia y Materiales de Aragón (INMA)-Departamento de Física de la Materia Condensada, CSIC-Universidad de Zaragoza, Zaragoza 50009, Spain.

Corresponding author's e-mail address: randreu@unizar.es

TABLE OF CONTENTS

1. NMR spectra for new compounds

Figures S-1 to S-2 (NMR spectra of compound **H1a**) pages S2-S3

Figures S-3 to S-4 (NMR spectra of compound **H1c**) pages S4-S5

Figures S-5 to S-6 (NMR spectra of compound **1Na**) pages S6-S7

Figure S-7 (^{1}H-NMR spectrum of compound **1Nb**) page S8

Figures S-8 to S-9 (NMR spectra of compound **2a**) pages S9-S10

Figures S-10 to S-11 (NMR spectra of compound **2c**) pages S11-S12

Figures S-12 to S-13 (NMR spectra of compound **6b**) pages S13-S14

2. UV-vis spectra pages S15-S26

(Figures S-14 to S-25)

3. Quantum Chemistry Calculations pages S27-S55

(Figures S-26 to S-27)

1. NMR spectra for new compounds

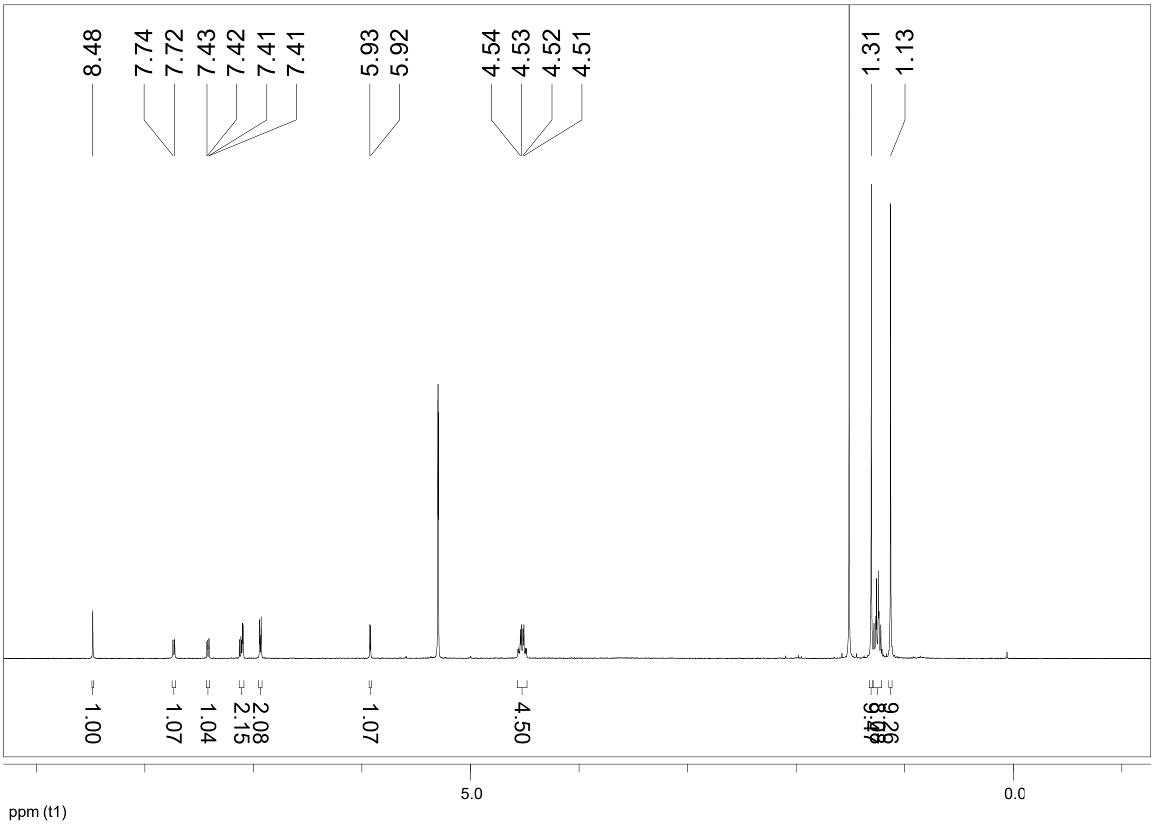

Figure S-1: ^{1}H NMR spectrum of compound **1Ha** (300 MHz, CD_2Cl_2).

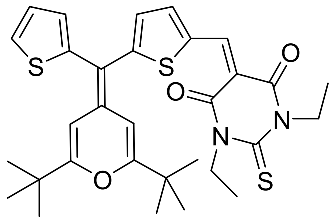

1Ha

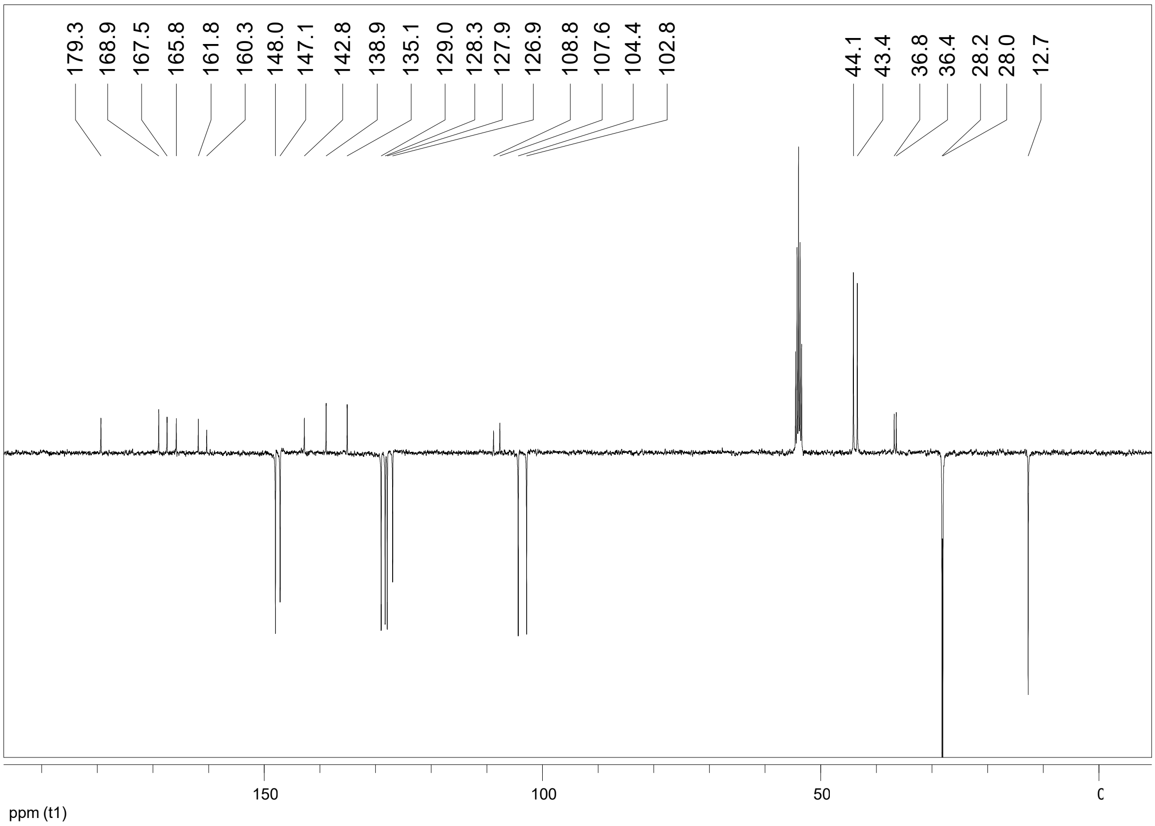

Figure S-2: $^{13}C\{^1H\}$ NMR (APT) spectrum of compound **1Ha** (100 MHz, CD_2Cl_2).

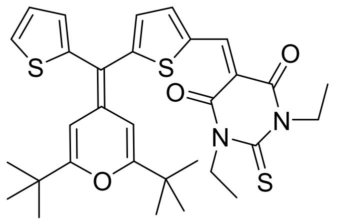

1Ha

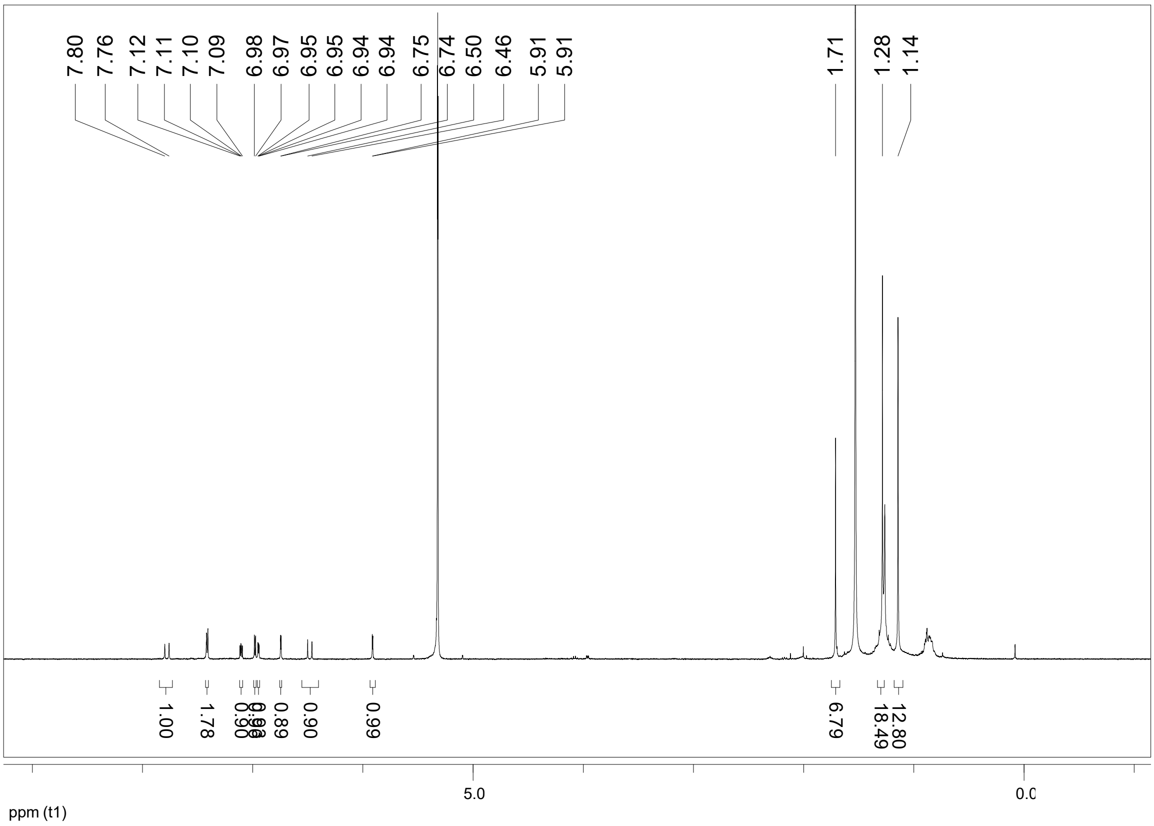

Figure S-3: ^{1}H NMR spectrum of compound **1Hc** (400 MHz, CD_2Cl_2).

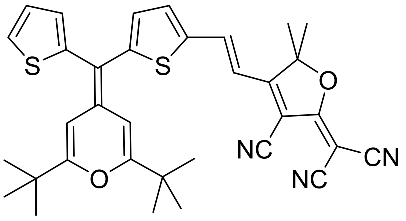

1Hc

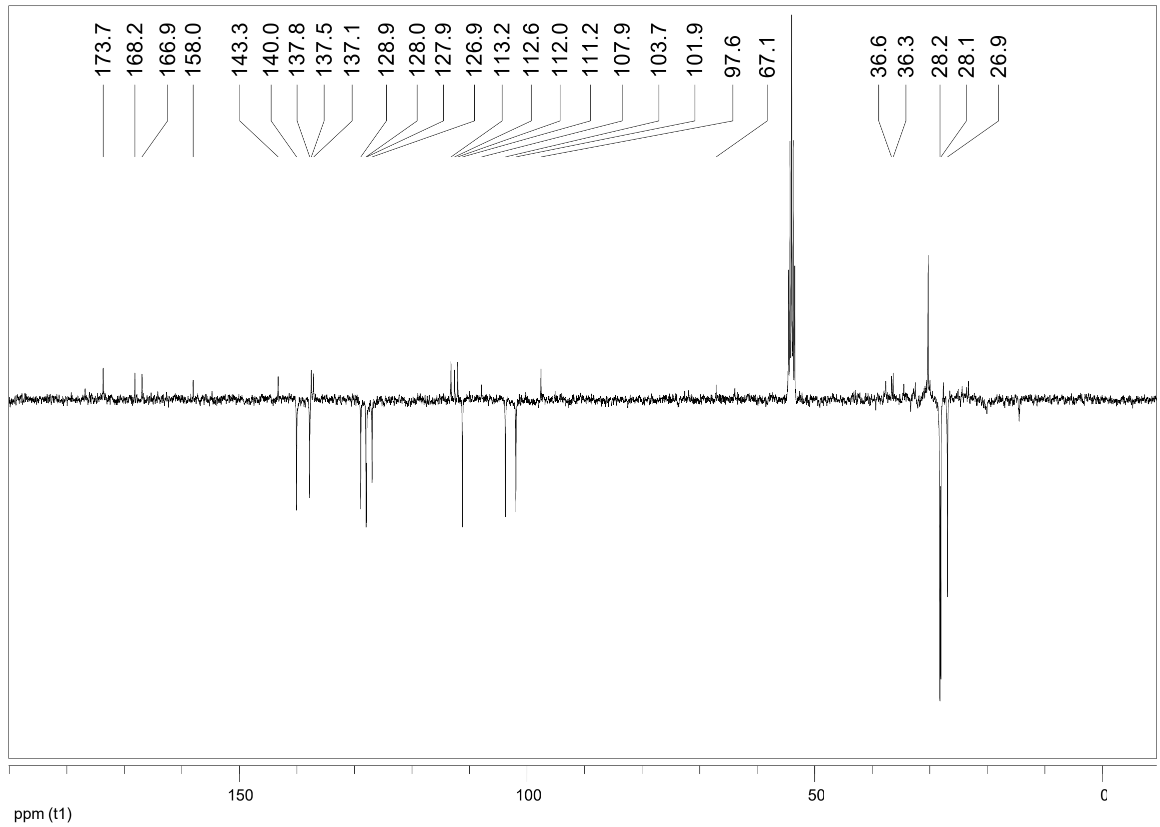

Figure S-4: $^{13}C\{^{1}H\}$ NMR (APT) spectrum of compound **1Hc** (100 MHz, CD_2Cl_2).

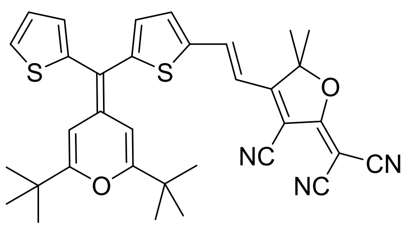

1Hc

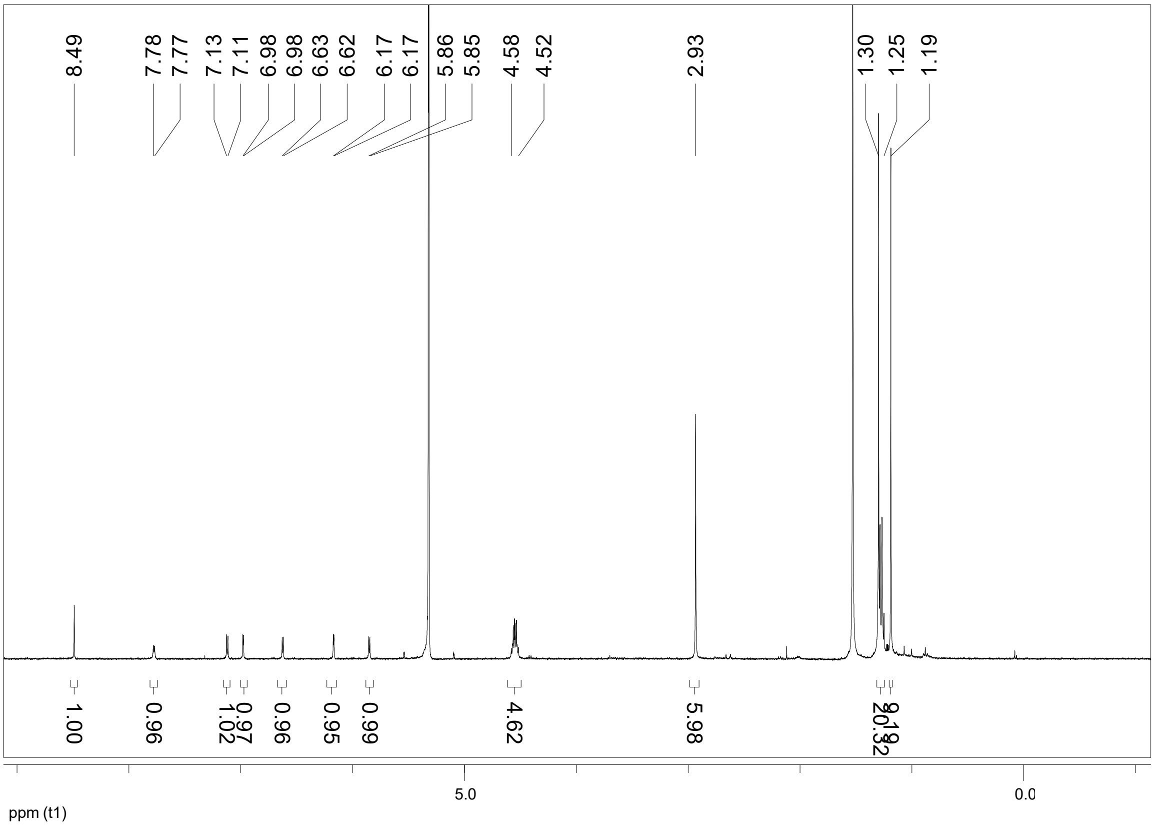

Figure S-5: ^{1}H NMR spectrum of compound **1Na** (400 MHz, CD_2Cl_2).

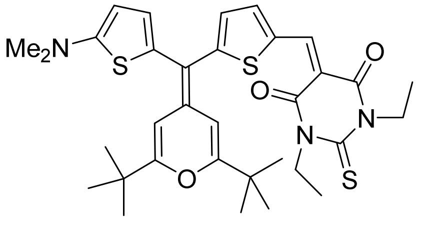

1Na

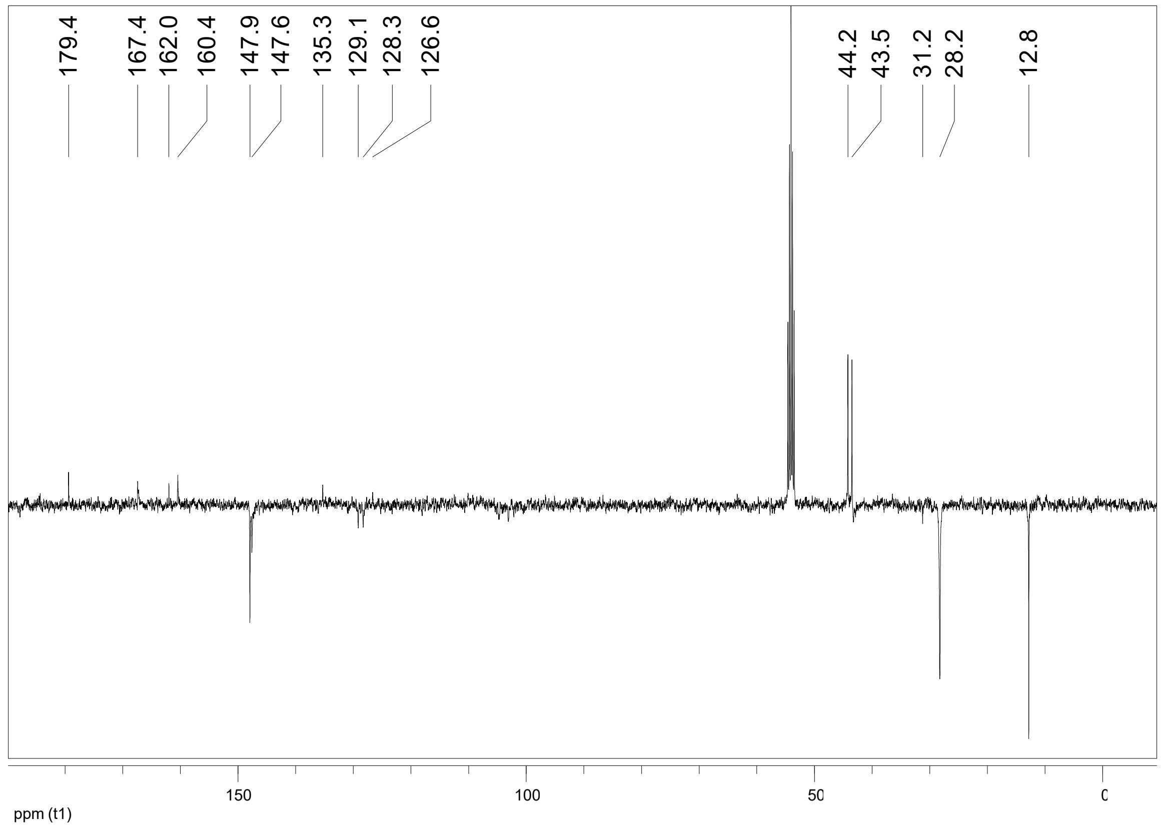

Figure S-6: $^{13}C\{^{1}H\}$ NMR (APT) spectrum of compound **1Na** (100 MHz, CD_2Cl_2).

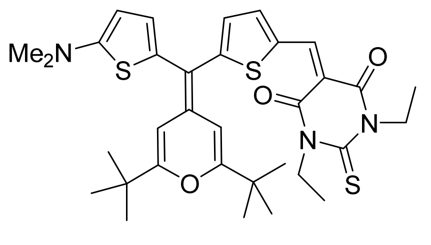

1Na

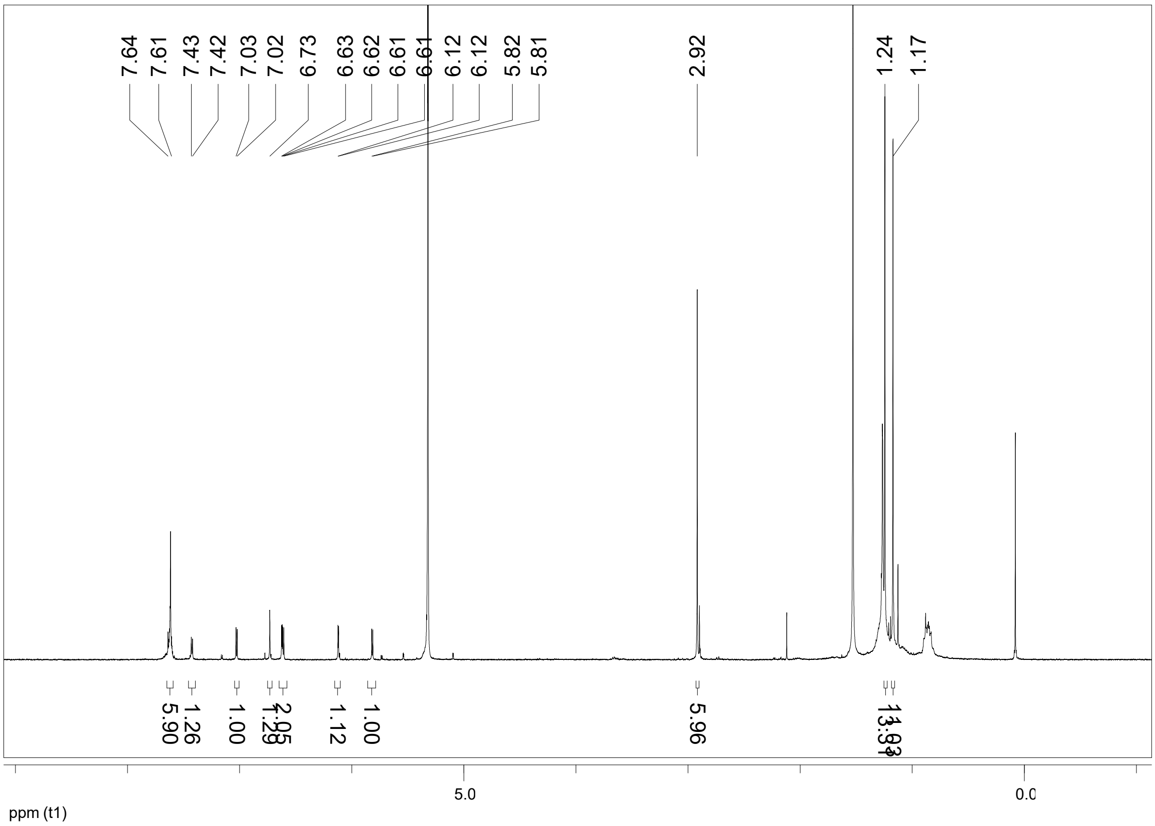

Figure S-7: ^{1}H NMR spectrum of compound **1Nb** (400 MHz, CD_2Cl_2).

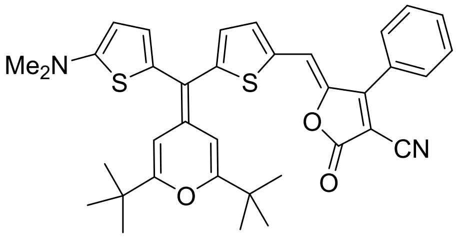

1Nb

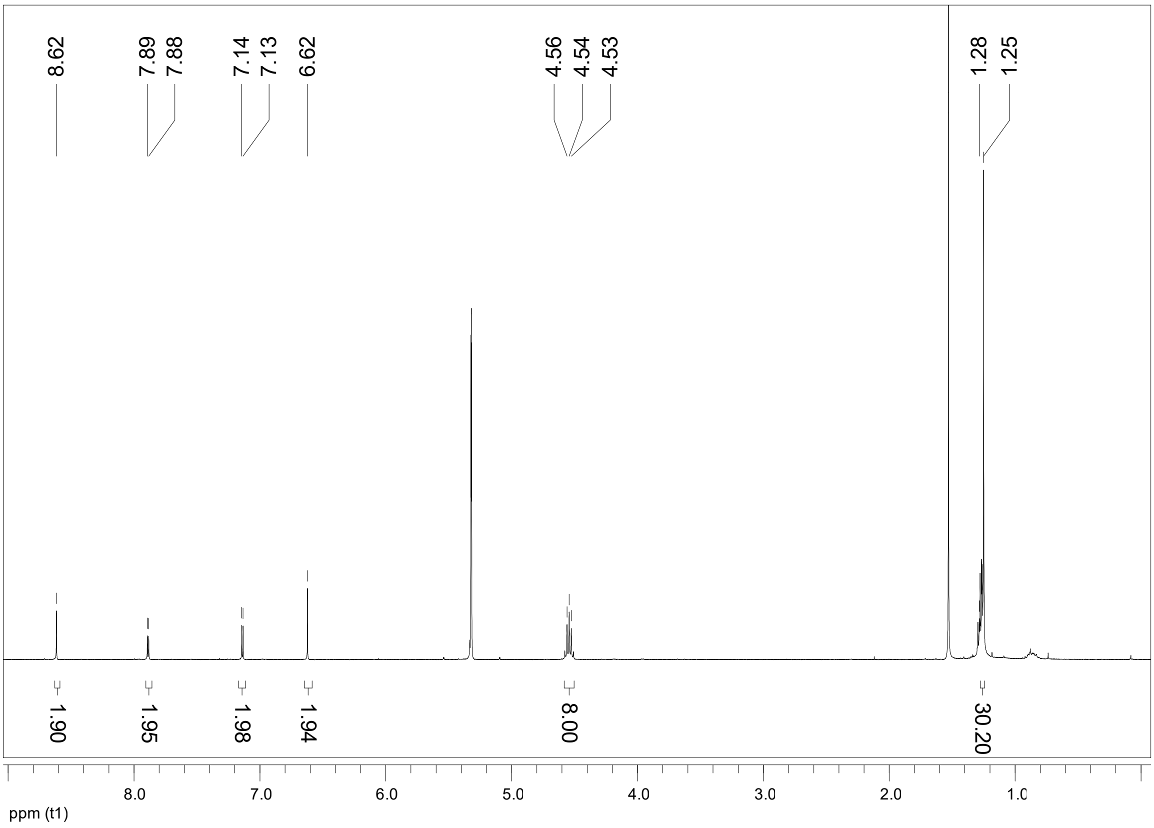

Figure S-8: ^{1}H NMR spectrum of compound **2a** (400 MHz, CD_2Cl_2).

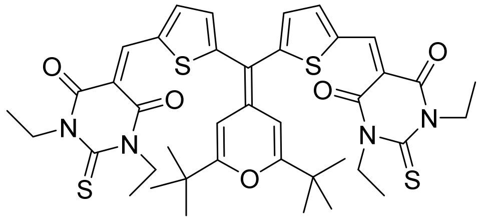

2a

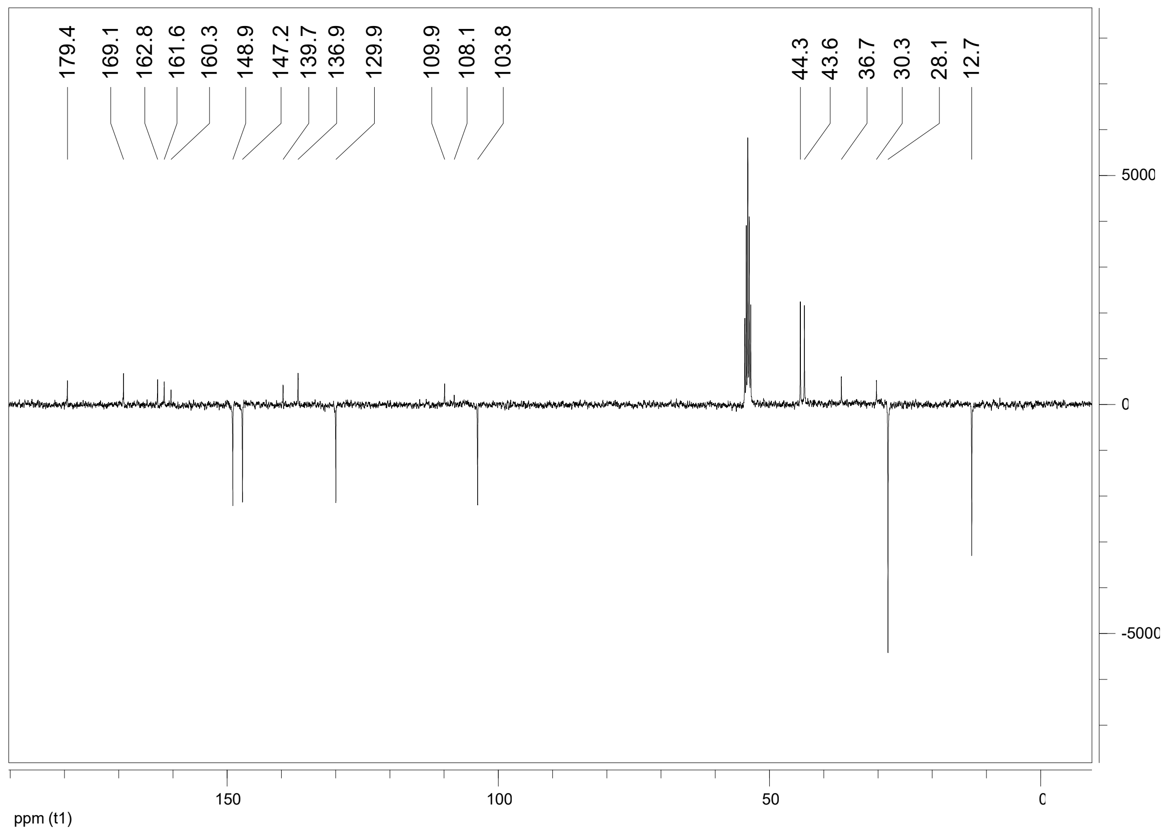

Figure S-9: $^{13}C\{^{1}H\}$ NMR (APT) spectrum of compound **2a** (100 MHz, CD_2Cl_2).

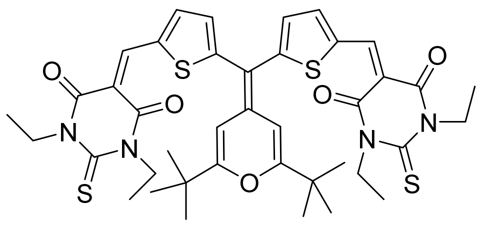

2a

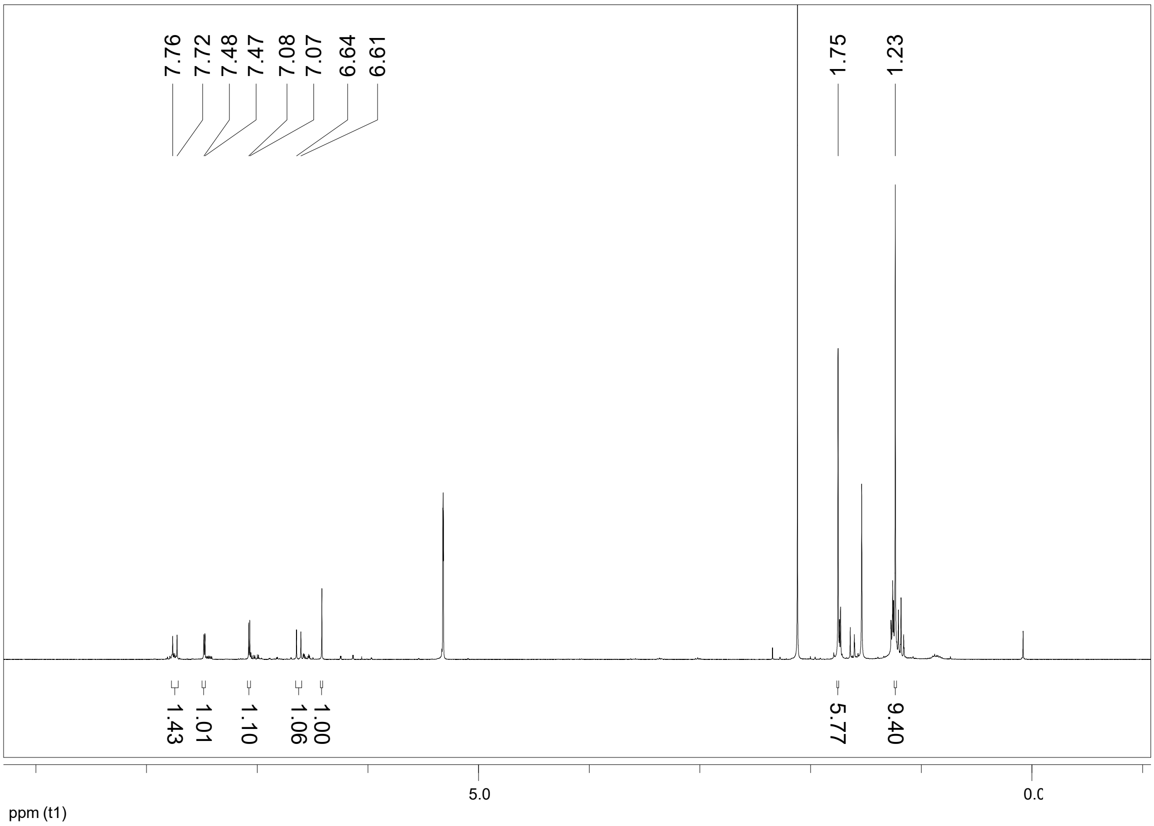

Figure S-10: ^{1}H NMR spectrum of compound **2c** (400 MHz, CD_2Cl_2).

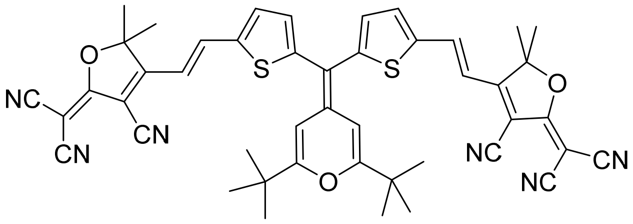

2c

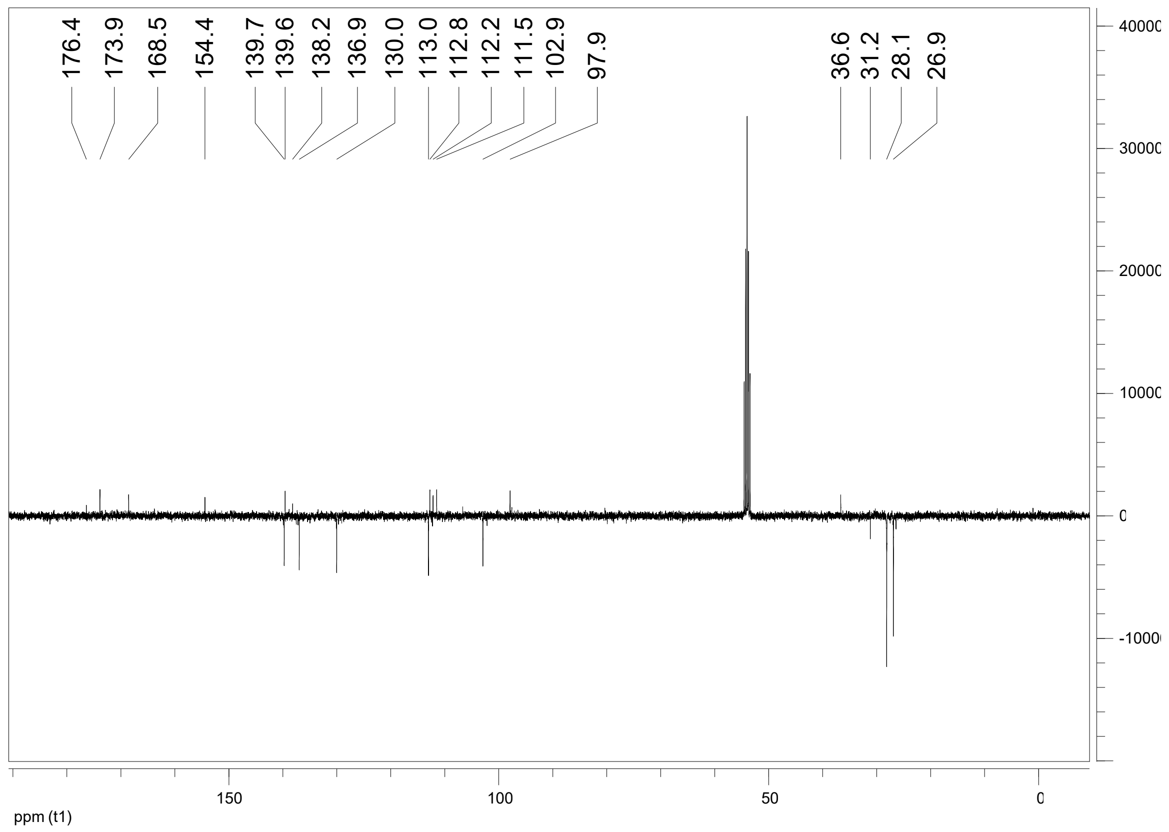

Figure S-11: $^{13}C\{^{1}H\}$ NMR (APT) spectrum of compound **2c** (100 MHz, CD_2Cl_2).

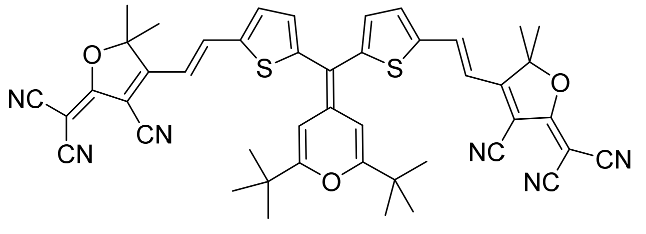

2c

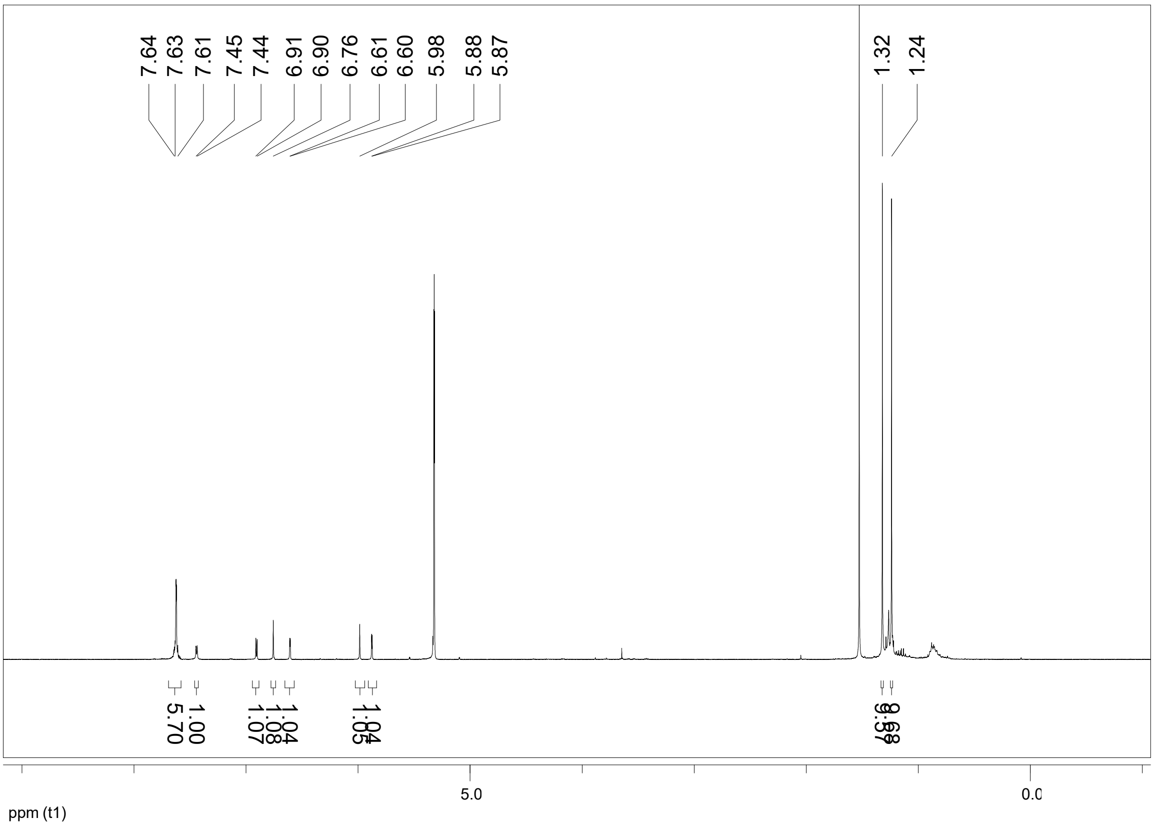

Figure S-12: ^{1}H NMR spectrum of compound **6b** (400 MHz, CD_2Cl_2).

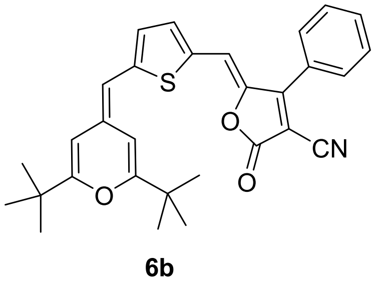

6b

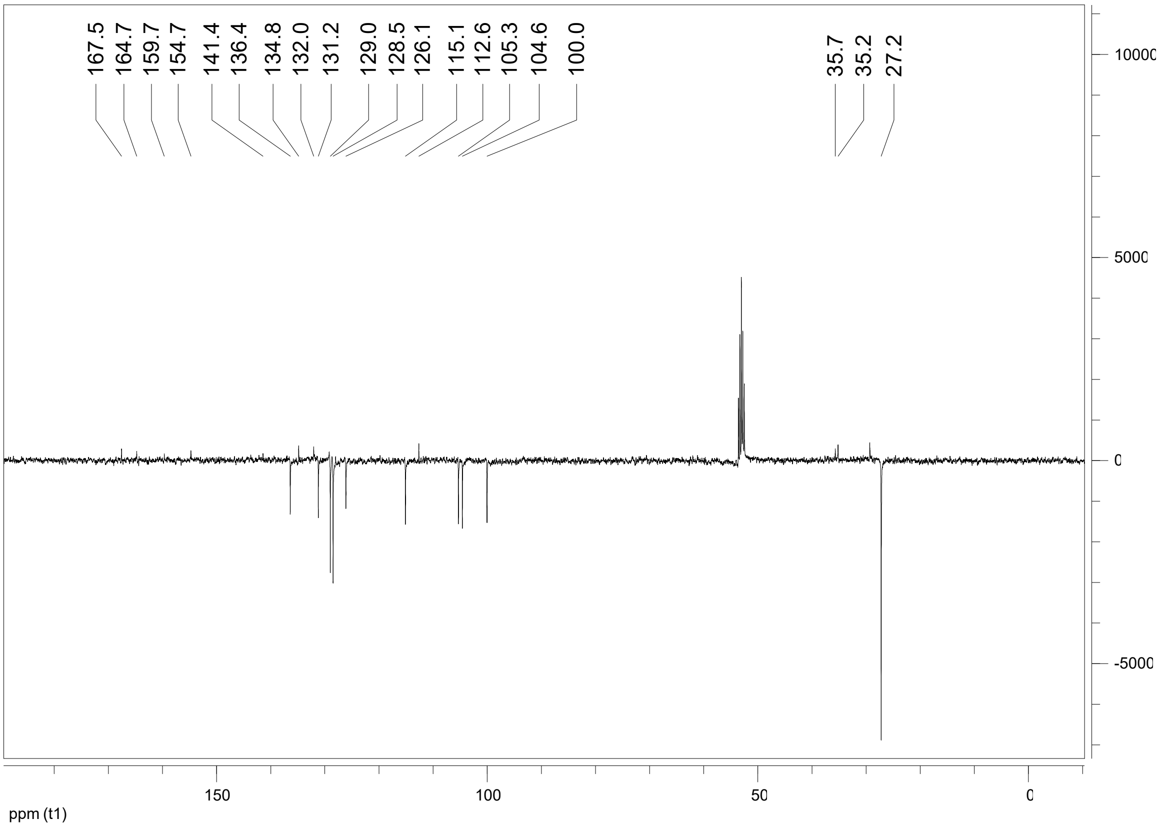

Figure S-13: $^{13}C\{^{1}H\}$ NMR (APT) spectrum of compound **6b** (100 MHz, CD_2Cl_2).

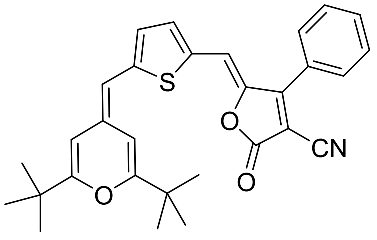

6b

2. UV-Vis spectra

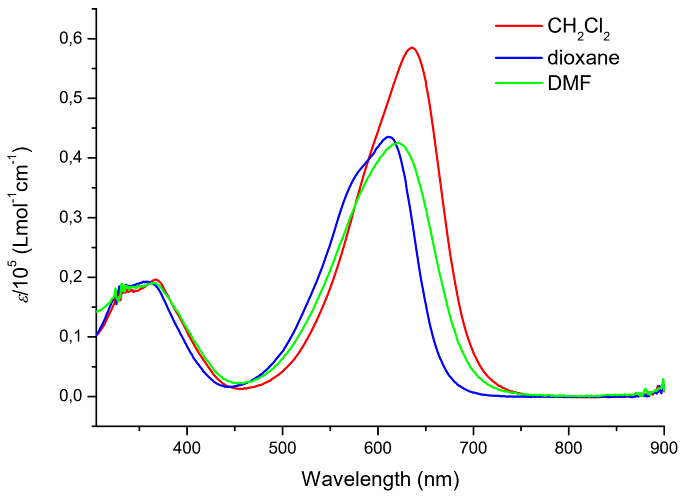

Figure S-14: UV-vis absorption spectra of compound **1Ha.**

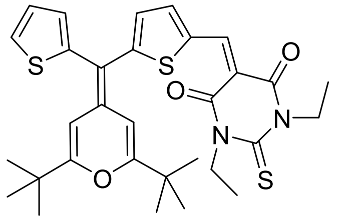

1Ha

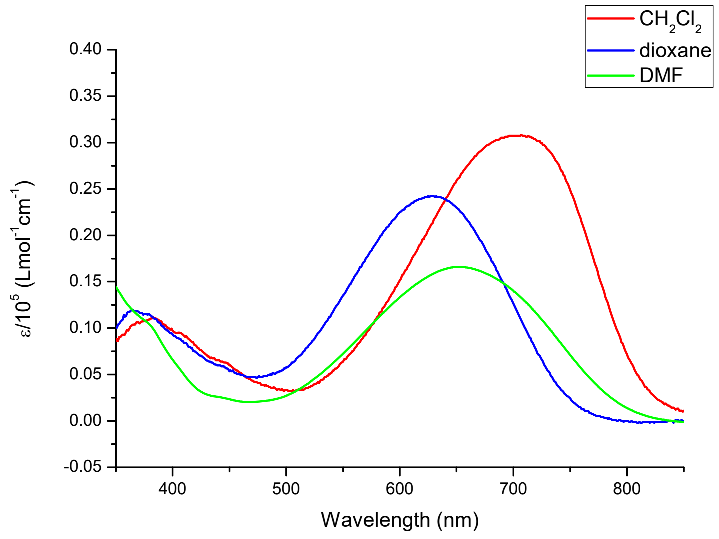

Figure S-15: UV-vis absorption spectra of compound **1Hc**.

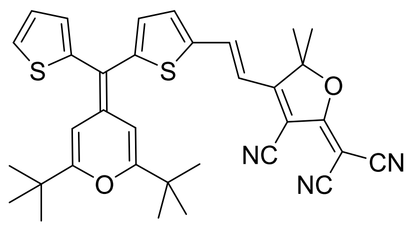

1Hc

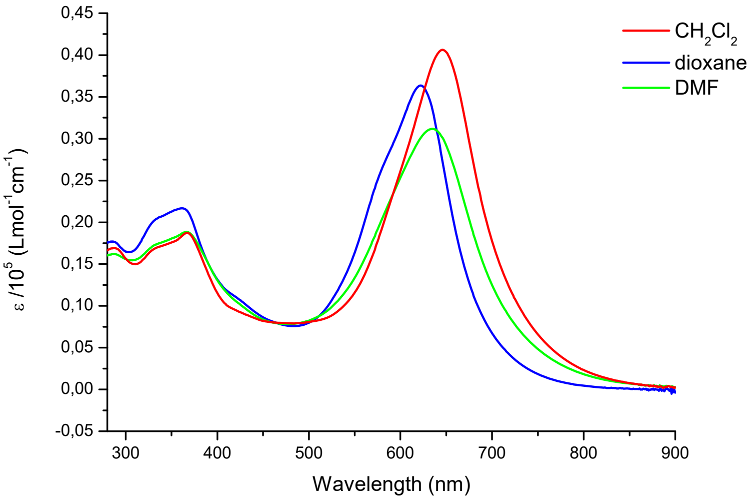

Figure S-16: UV-vis absorption spectra of compound **1Na**.

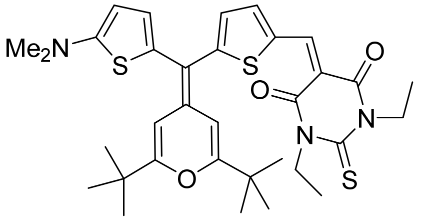

1Na

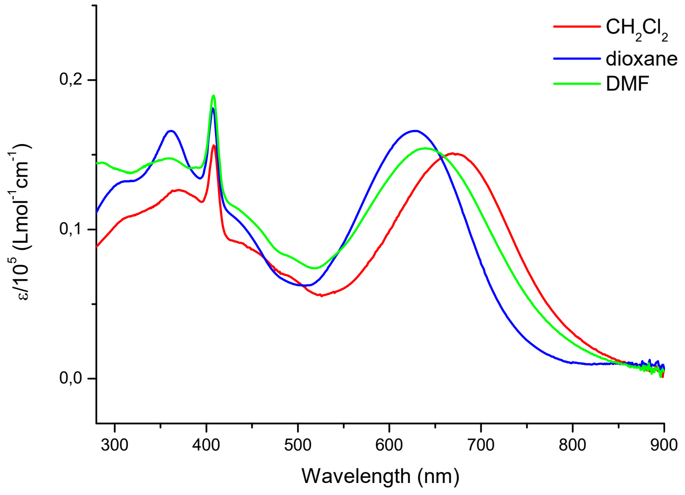

Figure S-17: UV-vis absorption spectra of compound **1Nb**.

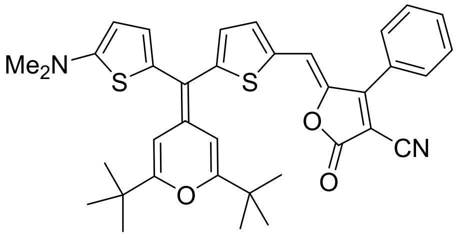

1Nb

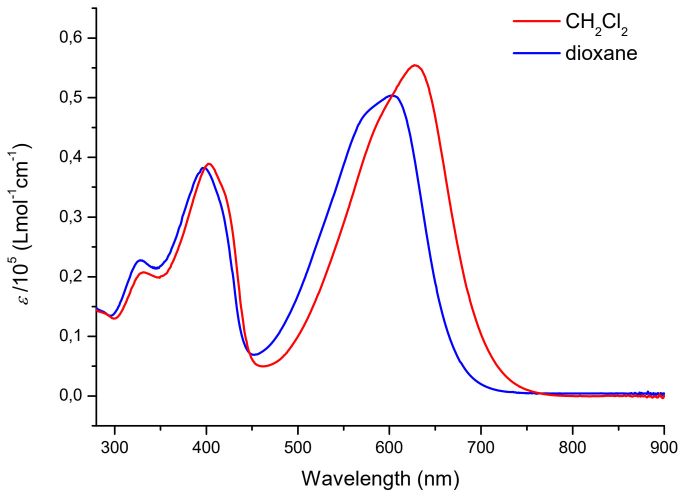

Figure S-18: UV-vis absorption spectra (in CH_2Cl_2 and dioxane) of compound **2a**.

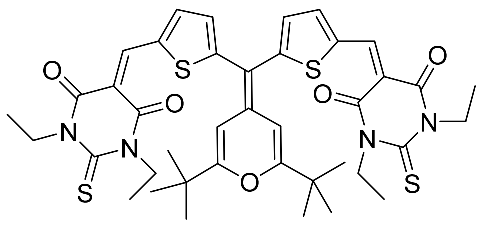

2a

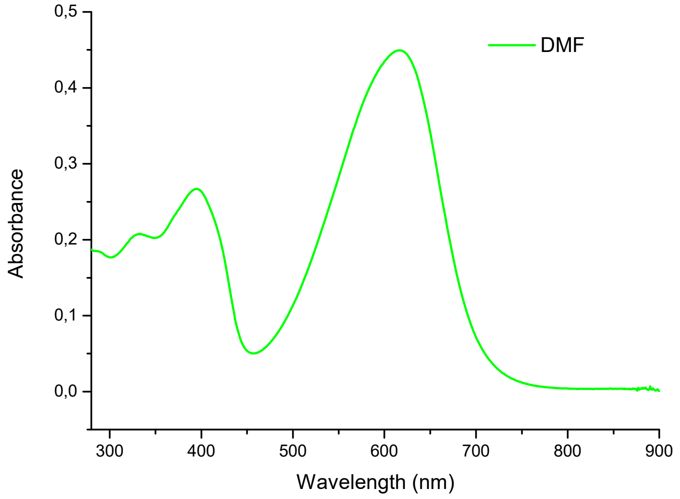

Figure S-19: UV-vis absorption spectrum in DMF of compound **2a**.

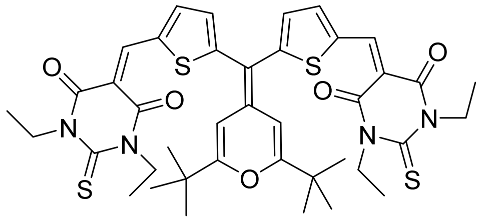

2a

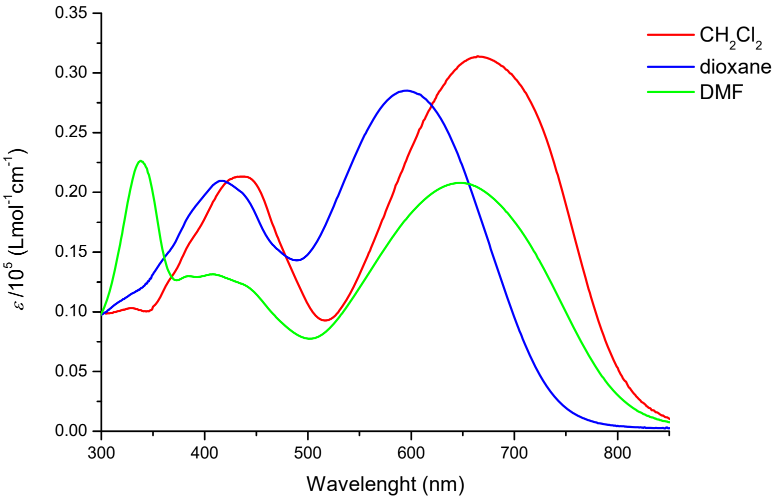

Figure S-20: UV-vis absorption spectra of compound **2c**.

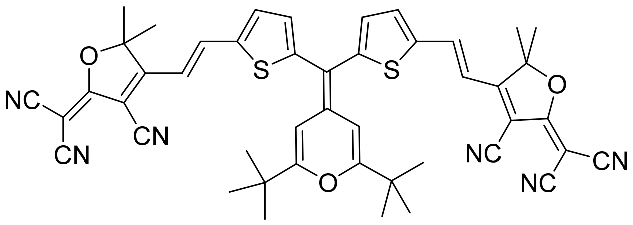

2c

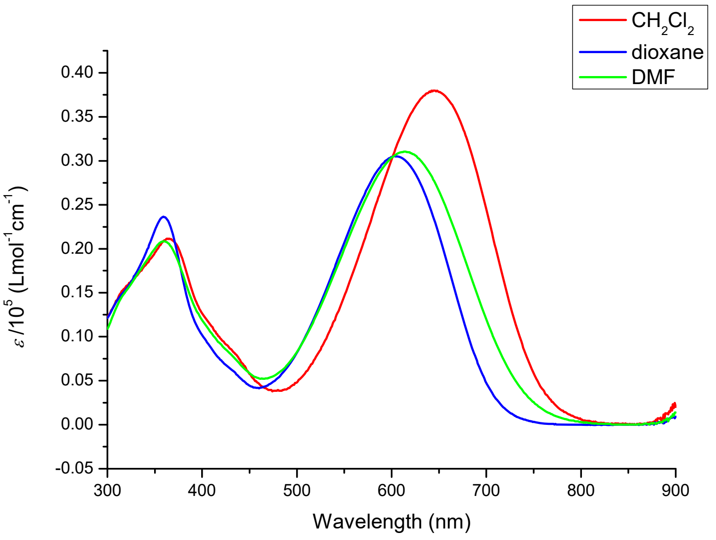

Figure S-21: UV-vis absorption spectra of compound **1Hb**.

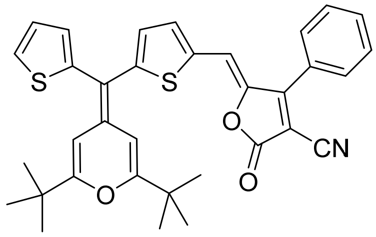

1Hb

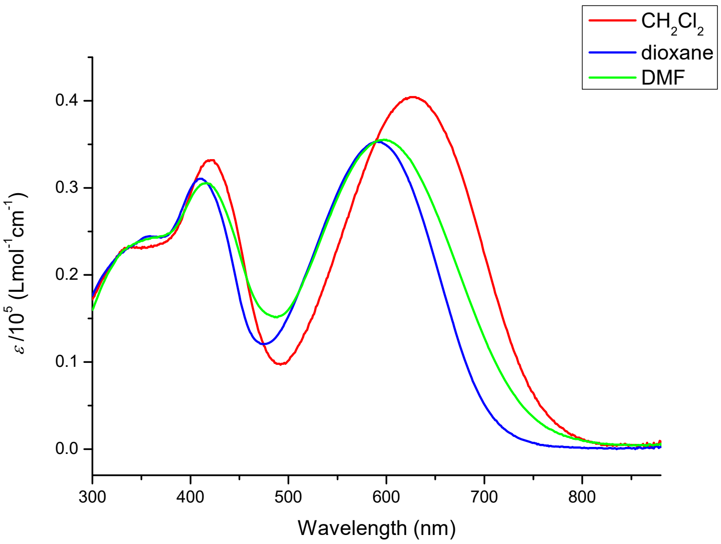

Figure S-22: UV-vis absorption spectra of compound **2b**.

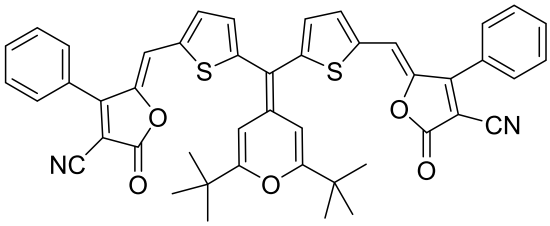

2b

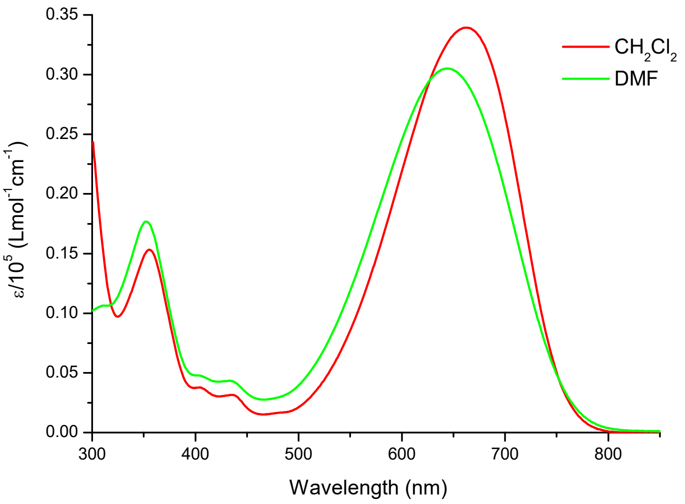

Figure S-23: UV-vis absorption spectra of compound **6b**

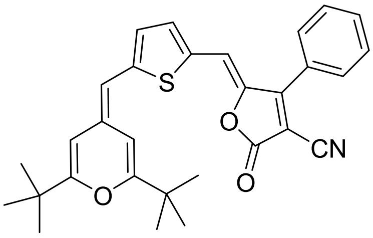

6b

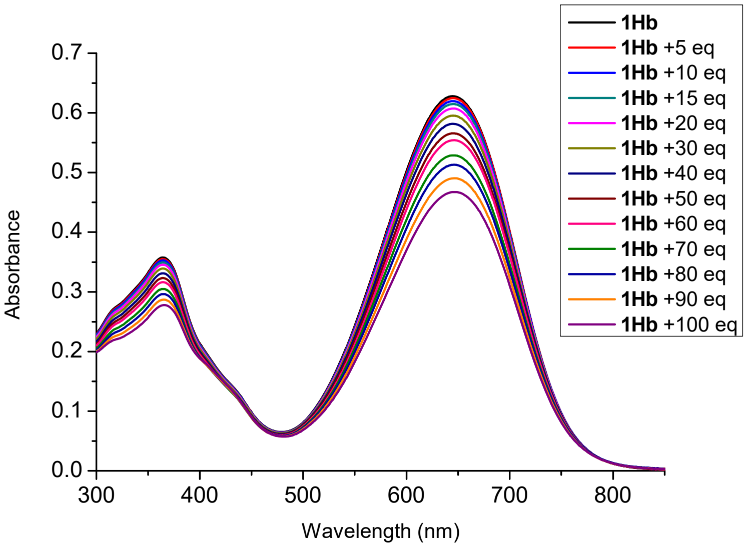

Figure S-24. Changes in the absorption spectra of a CH_2Cl_2 solution of compound **1Hb** (c = 3.1×10^{-5} M) upon addition of TFA (5–100 eq.).

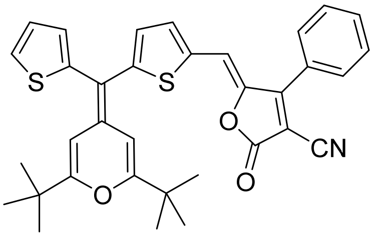

1Hb

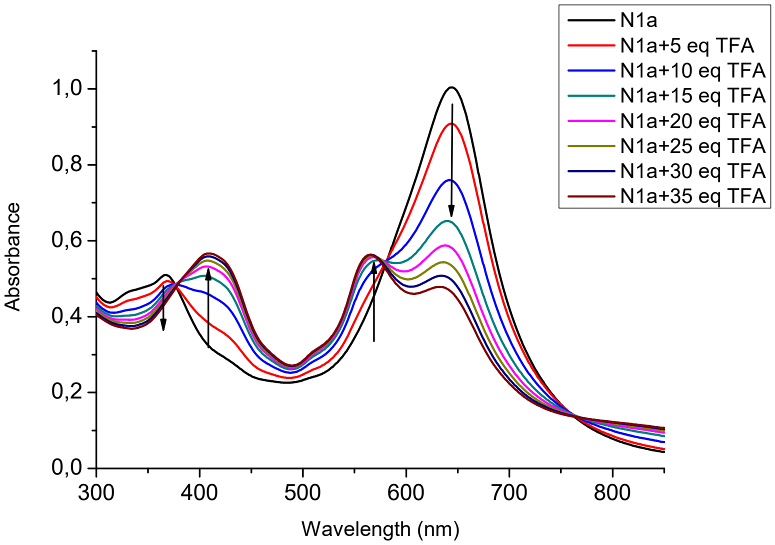

Figure S-25: Changes in the absorption spectra of a CH_2Cl_2 solution of compound **1Na** (c = 3.1×10^{-5} M) upon addition of TFA (5–35 eq.)

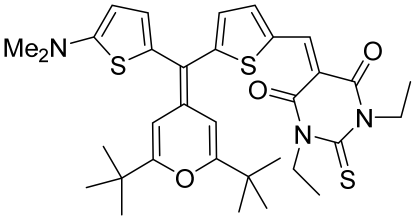

1Na

3. Quantum Chemistry Calculations

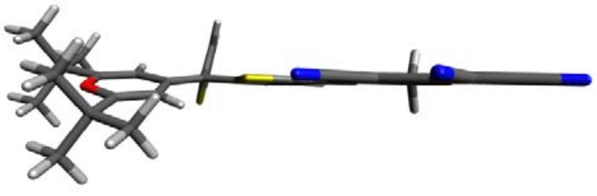

Figure S-26. Optimized geometry (CPCM-M06-2x/6-31G*) for **1Hc** in CH_2Cl_2.

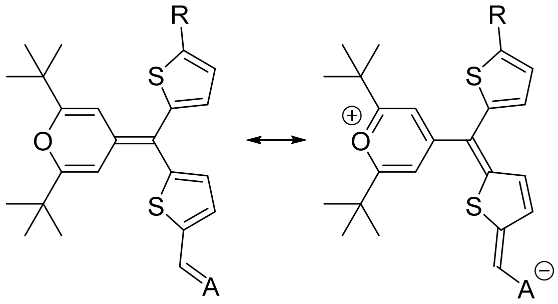

Figure S-27. Resonance forms for the studied dyes.

Optimized geometries (CPCM-M06-2x/6-31G*) and calculated energies

1Ha. Conformation A.

Standard orientation:

Center Number	Atomic Number	Atomic Type	Coordinates (Angstroms) X	Y	Z
1	6	0	-4.819153	1.182925	-0.266104
2	6	0	-4.289048	-0.057407	-0.237275
3	6	0	-2.891924	-0.283867	0.067882
4	6	0	-2.174352	0.920899	0.415042
5	6	0	-2.767048	2.134455	0.382907
6	1	0	-4.921200	-0.897156	-0.490028
7	1	0	-1.153696	0.884145	0.758498
8	8	0	-4.064736	2.277664	0.021098

9	6	0	-2.351257	-1.562332	0.025004
10	6	0	-0.958255	-1.917530	0.146609
11	6	0	-0.487934	-3.235430	0.271014
12	16	0	0.377198	-0.806108	0.060594
13	6	0	0.894381	-3.321407	0.311177
14	1	0	-1.153319	-4.087198	0.326530
15	6	0	1.560521	-2.091402	0.210771
16	1	0	1.441521	-4.252511	0.415302
17	6	0	-3.269774	-2.711238	-0.172663
18	6	0	-3.493725	-3.442970	-1.305108
19	16	0	-4.221052	-3.328885	1.148514
20	6	0	-4.439609	-4.498406	-1.120315
21	1	0	-2.994271	-3.228356	-2.243536
22	6	0	-4.917263	-4.555024	0.155169
23	1	0	-4.748983	-5.179209	-1.904402
24	6	0	2.966038	-2.036063	0.252600
25	1	0	3.405425	-3.028244	0.352580
26	6	0	-6.240259	1.570146	-0.604270
27	6	0	-6.217276	2.511805	-1.820891
28	6	0	-7.081984	0.334571	-0.928192
29	6	0	-6.848298	2.303721	0.604153
30	1	0	-5.636178	3.414064	-1.611096
31	1	0	-5.783167	2.011886	-2.692599
32	1	0	-7.240470	2.811136	-2.068771
33	1	0	-7.124703	-0.355258	-0.079077
34	1	0	-8.103616	0.649003	-1.160373
35	1	0	-6.687908	-0.202904	-1.796597
36	1	0	-7.875886	2.599621	0.370781
37	1	0	-6.867870	1.654163	1.485058
38	1	0	-6.277785	3.204346	0.847303
39	6	0	-2.133686	3.464550	0.721278
40	6	0	-2.912016	4.097750	1.887646
41	6	0	-0.665766	3.289762	1.118210
42	6	0	-2.226442	4.375189	-0.515975
43	1	0	-3.961388	4.254368	1.622927
44	1	0	-2.867374	3.461626	2.777327
45	1	0	-2.470795	5.068254	2.135139
46	1	0	-0.078702	2.838294	0.311143
47	1	0	-0.237299	4.271687	1.339097
48	1	0	-0.562980	2.668837	2.014109
49	1	0	-1.784388	5.349057	-0.283793
50	1	0	-1.681925	3.941616	-1.360774
51	1	0	-3.266807	4.531572	-0.814158
52	1	0	-5.638486	-5.245878	0.569885
53	6	0	3.928780	-1.046439	0.203962
54	6	0	3.643968	0.362554	0.066227
55	6	0	5.312953	-1.499568	0.291085
56	7	0	4.747210	1.238435	0.039746
57	7	0	6.307949	-0.502851	0.244475
58	6	0	6.062292	0.836120	0.054383
59	6	0	7.694015	-1.008138	0.315456
60	6	0	8.213338	-1.406282	-1.058498
61	1	0	7.666756	-1.868079	0.982063
62	1	0	8.303577	-0.227954	0.763961
63	1	0	9.237116	-1.780321	-0.972573
64	1	0	7.590673	-2.197160	-1.484451
65	1	0	8.213839	-0.547151	-1.734283
66	6	0	4.401286	2.666441	-0.111144
67	6	0	4.231379	3.049891	-1.574088
68	1	0	5.188607	3.244386	0.365881
69	1	0	3.471456	2.810708	0.436384
70	1	0	3.969142	4.108627	-1.650254
71	1	0	5.159305	2.882195	-2.126812
72	1	0	3.431100	2.461521	-2.030132
73	16	0	7.316461	1.936380	-0.153348
74	8	0	5.643766	-2.671308	0.392588
75	8	0	2.514663	0.823808	-0.034724

6-31G* Energies:

SCF Done: E (RM062X) = –2732.00207204 a.u.
Thermal correction to Gibbs Free Energy = 0.5440 a.u.
Single point 6-311+G(2d,p) energy: –2732.5558 a.u.

1Ha. Conformation B.

Standard orientation:

Center Number	Atomic Number	Atomic Type	Coordinates (Angstroms) X	Y	Z
1	6	0	-5.603825	-0.352598	0.161605
2	6	0	-4.383392	-0.883476	-0.050768
3	6	0	-3.197646	-0.053917	-0.131637
4	6	0	-3.447488	1.349483	0.127458
5	6	0	-4.691842	1.812416	0.363911
6	1	0	-4.294492	-1.951271	-0.195599
7	1	0	-2.622807	2.043773	0.191070
8	8	0	-5.770715	0.985752	0.349294
9	6	0	-1.950205	-0.588904	-0.394192
10	6	0	-0.775555	0.218018	-0.657794
11	6	0	-0.677506	1.474952	-1.259355
12	16	0	0.790011	-0.400158	-0.237814
13	6	0	0.637274	1.920308	-1.357534
14	1	0	-1.528565	2.014916	-1.654544
15	6	0	1.592137	1.030725	-0.851249
16	1	0	0.925151	2.865397	-1.806146
17	6	0	-1.763904	-2.055972	-0.372582
18	6	0	-1.872161	-2.909096	0.692746
19	16	0	-1.369974	-2.942841	-1.820397
20	6	0	-1.633393	-4.275351	0.353157
21	1	0	-2.111417	-2.560173	1.691538
22	6	0	-1.346466	-4.441578	-0.969364
23	1	0	-1.666326	-5.091840	1.064557
24	6	0	2.963252	1.370217	-0.908181
25	1	0	3.117239	2.349522	-1.360529
26	6	0	-6.927581	-1.079995	0.220624
27	6	0	-7.844793	-0.521063	-0.881071
28	6	0	-6.736806	-2.583498	0.013354
29	6	0	-7.565605	-0.828090	1.597944
30	1	0	-8.011797	0.551544	-0.749014
31	1	0	-7.409490	-0.687068	-1.871677
32	1	0	-8.814006	-1.028149	-0.840779
33	1	0	-6.094661	-3.016483	0.787108
34	1	0	-7.710850	-3.078605	0.065950
35	1	0	-6.299219	-2.800771	-0.966250
36	1	0	-8.530080	-1.342750	1.652962
37	1	0	-6.925175	-1.209097	2.399882
38	1	0	-7.735260	0.239220	1.764701
39	6	0	-5.098298	3.234720	0.676563
40	6	0	-5.763296	3.262101	2.063707
41	6	0	-3.885563	4.166997	0.671686
42	6	0	-6.107395	3.700495	-0.387699
43	1	0	-6.646975	2.618539	2.090086
44	1	0	-5.064727	2.927498	2.837256
45	1	0	-6.074706	4.284700	2.299143
46	1	0	-3.388709	4.173313	-0.304149
47	1	0	-4.217012	5.186627	0.888713
48	1	0	-3.155063	3.880334	1.434938
49	1	0	-6.421736	4.725569	-0.167695
50	1	0	-5.656984	3.684393	-1.385354
51	1	0	-6.995255	3.062357	-0.395715
52	1	0	-1.119208	-5.359370	-1.494243
53	6	0	4.150285	0.782212	-0.533632

54	6	0	4.254372	-0.511067	0.106629
55	6	0	5.360900	1.556513	-0.802093
56	7	0	5.546852	-0.952182	0.447963
57	7	0	6.580713	0.975838	-0.407847
58	6	0	6.694859	-0.214382	0.272563
59	6	0	7.776419	1.797305	-0.686510
60	6	0	8.039553	2.798271	0.429162
61	1	0	7.576158	2.309237	-1.625982
62	1	0	8.614065	1.117835	-0.821710
63	1	0	8.924299	3.394203	0.189413
64	1	0	7.187636	3.473681	0.541445
65	1	0	8.215556	2.282179	1.376451
66	6	0	5.595550	-2.265884	1.121938
67	6	0	5.390069	-2.131302	2.623834
68	1	0	6.556472	-2.716583	0.887458
69	1	0	4.802777	-2.863021	0.675063
70	1	0	5.424417	-3.119230	3.090939
71	1	0	6.174111	-1.511351	3.066098
72	1	0	4.416163	-1.682562	2.835434
73	16	0	8.173935	-0.744416	0.865481
74	8	0	5.362658	2.656704	-1.331622
75	8	0	3.292089	-1.218060	0.368918

6-31G* Energies:

SCF Done: E (RM062X) = –2732.00217183 a.u.
Thermal correction to Gibbs Free Energy = 0.5441 a.u.
Single point 6-311+G(2d,p) energy: –2732.5555 a.u.

1Na. Conformation A.

Standard orientation:

Center Number	Atomic Number	Atomic Type	Coordinates (Angstroms) X	Y	Z
1	6	0	4.020110	2.346345	0.341785
2	6	0	3.715439	1.032043	0.357966
3	6	0	2.393602	0.550803	0.016256
4	6	0	1.495279	1.593252	-0.423113
5	6	0	1.865921	2.892255	-0.433634
6	1	0	4.469207	0.326357	0.678300
7	1	0	0.514320	1.360145	-0.803776
8	8	0	3.100717	3.277797	-0.030378
9	6	0	2.081056	-0.800317	0.109977
10	6	0	0.768740	-1.385666	-0.025934
11	6	0	0.524746	-2.765829	-0.128511
12	16	0	-0.733883	-0.509897	0.022517
13	6	0	-0.822910	-3.080688	-0.182139
14	1	0	1.324862	-3.493966	-0.159456
15	6	0	-1.686193	-1.976299	-0.113823
16	1	0	-1.206477	-4.091449	-0.274641
17	6	0	3.172162	-1.766384	0.372976
18	6	0	3.440099	-2.490030	1.495108
19	16	0	4.302397	-2.190025	-0.900889
20	6	0	4.546555	-3.385491	1.373078
21	1	0	2.844775	-2.395382	2.397566
22	6	0	5.136837	-3.331222	0.133081
23	1	0	4.883448	-4.034527	2.170618
24	6	0	-3.079718	-2.154630	-0.175953
25	1	0	-3.348338	-3.207366	-0.259715
26	6	0	5.334287	2.992976	0.714173
27	6	0	5.091037	3.963296	1.883253
28	6	0	6.364425	1.940344	1.127432
29	6	0	5.858212	3.774300	-0.503651
30	1	0	4.370274	4.738710	1.609290
31	1	0	4.712596	3.429186	2.760600
32	1	0	6.033176	4.449546	2.155140
33	1	0	6.563938	1.234117	0.314944
34	1	0	7.304169	2.439837	1.380370
35	1	0	6.033076	1.378193	2.006306
36	1	0	6.806290	4.256857	-0.246212
37	1	0	6.031252	3.104194	-1.351802
38	1	0	5.149982	4.549094	-0.809992
39	6	0	1.027554	4.073641	-0.865183
40	6	0	1.737670	4.782398	-2.031501
41	6	0	-0.367100	3.625388	-1.308861
42	6	0	0.901337	5.039744	0.326274
43	1	0	2.730837	5.131106	-1.735147
44	1	0	1.844891	4.110397	-2.888914
45	1	0	1.147529	5.649354	-2.344541
46	1	0	-0.903258	3.112184	-0.503198
47	1	0	-0.949875	4.505633	-1.595414
48	1	0	-0.317679	2.958079	-2.175463
49	1	0	0.305871	5.908246	0.028088
50	1	0	0.403301	4.553363	1.171032
51	1	0	1.883028	5.391650	0.655218
52	6	0	-4.194108	-1.336348	-0.164455
53	6	0	-4.148577	0.102399	-0.055308
54	6	0	-5.482036	-2.013761	-0.263205
55	7	0	-5.381659	0.784614	-0.072324
56	7	0	-6.628884	-1.194286	-0.260696
57	6	0	-6.611571	0.170657	-0.101380
58	6	0	-7.910741	-1.922734	-0.345699
59	6	0	-8.391498	-2.368616	1.027647
60	1	0	-7.725917	-2.781803	-0.987885
61	1	0	-8.629037	-1.264479	-0.827631
62	1	0	-9.336737	-2.909563	0.930810

63	1	0	-7.657532	-3.034591	1.488451
64	1	0	-8.551345	-1.505611	1.679161
65	6	0	-5.278775	2.252971	0.048668
66	6	0	-5.207112	2.690739	1.504455
67	1	0	-6.139932	2.682367	-0.456896
68	1	0	-4.373256	2.536494	-0.485098
69	1	0	-5.124169	3.779592	1.559275
70	1	0	-6.107351	2.384278	2.043352
71	1	0	-4.331688	2.252081	1.990025
72	16	0	-8.034285	1.053774	0.053623
73	8	0	-5.613623	-3.226252	-0.339663
74	8	0	-3.113952	0.747031	0.057116
75	7	0	6.263811	-3.993944	-0.315974
76	6	0	6.732423	-5.059434	0.554432
77	1	0	5.990375	-5.864530	0.667046
78	1	0	7.649666	-5.476400	0.135746
79	1	0	6.959400	-4.653838	1.543731
80	6	0	6.351447	-4.272330	-1.741968
81	1	0	6.265564	-3.342585	-2.311178
82	1	0	7.331268	-4.703274	-1.954870
83	1	0	5.572217	-4.969241	-2.084346

6-31G* Energies:

SCF Done: E (RM062X) = –2865.9080627 a.u.
Thermal correction to Gibbs Free Energy = 0.6121 a.u.
Single point 6-311+G(2d,p) energy: –2866.5018 a.u.

1Na. Conformation B.

Standard orientation:

Center Number	Atomic Number	Atomic Type	Coordinates (Angstroms) X	Y	Z
1	6	0	5.516124	-0.304796	0.285136
2	6	0	4.316297	0.291496	0.141031
3	6	0	3.100334	-0.477932	-0.032281
4	6	0	3.299137	-1.910667	0.056828
5	6	0	4.525111	-2.443908	0.233671
6	1	0	4.265604	1.371242	0.121000
7	1	0	2.449455	-2.577103	0.039253
8	8	0	5.634194	-1.661703	0.315628
9	6	0	1.871249	0.129640	-0.220328
10	6	0	0.677566	-0.603991	-0.591953
11	6	0	0.549042	-1.765808	-1.358345
12	16	0	-0.872812	-0.003811	-0.098314
13	6	0	-0.776029	-2.156227	-1.520760
14	1	0	1.388595	-2.272061	-1.817841
15	6	0	-1.710346	-1.315600	-0.902364
16	1	0	-1.086663	-3.023790	-2.093704
17	6	0	1.728395	1.581543	-0.002010
18	6	0	1.934971	2.303896	1.137289
19	16	0	1.298131	2.656668	-1.322821
20	6	0	1.754914	3.711107	0.989793
21	1	0	2.221062	1.831756	2.071903
22	6	0	1.386316	4.064899	-0.287374
23	1	0	1.884230	4.420558	1.796384
24	6	0	-3.089097	-1.602630	-1.013947
25	1	0	-3.266843	-2.504999	-1.598345
26	6	0	6.865913	0.361852	0.422380
27	6	0	7.760619	-0.095279	-0.742897
28	6	0	6.730315	1.885265	0.395639
29	6	0	7.496157	-0.073996	1.756688

30	1	0	7.889196	-1.181221	-0.739237
31	1	0	7.329423	0.201645	-1.704309
32	1	0	8.747582	0.368800	-0.649467
33	1	0	6.103425	2.246340	1.217361
34	1	0	7.721724	2.335080	0.503086
35	1	0	6.301612	2.232907	-0.549662
36	1	0	8.479466	0.394245	1.866268
37	1	0	6.871577	0.233743	2.601469
38	1	0	7.625436	-1.159107	1.796007
39	6	0	4.878642	-3.907388	0.372776
40	6	0	5.540901	-4.125143	1.744283
41	6	0	3.632685	-4.787662	0.260495
42	6	0	5.871009	-4.279187	-0.742997
43	1	0	6.445943	-3.519862	1.845465
44	1	0	4.853363	-3.862897	2.554762
45	1	0	5.817029	-5.178701	1.853546
46	1	0	3.137126	-4.659566	-0.707586
47	1	0	3.926217	-5.837377	0.353440
48	1	0	2.912191	-4.567342	1.054633
49	1	0	6.144980	-5.334857	-0.650396
50	1	0	5.423410	-4.124312	-1.729995
51	1	0	6.782903	-3.679589	-0.675974
52	6	0	-4.263364	-1.035741	-0.569739
53	6	0	-4.336228	0.156295	0.245991
54	6	0	-5.492561	-1.727847	-0.951349
55	7	0	-5.619417	0.586425	0.635292
56	7	0	-6.699697	-1.170544	-0.488257
57	6	0	-6.786713	-0.082589	0.348963
58	6	0	-7.915074	-1.912169	-0.881848
59	6	0	-8.216850	-3.044432	0.089176
60	1	0	-7.719736	-2.300298	-1.879781
61	1	0	-8.732441	-1.197310	-0.930651
62	1	0	-9.115234	-3.577914	-0.232842
63	1	0	-7.384913	-3.752748	0.116161
64	1	0	-8.387859	-2.653687	1.095535
65	6	0	-5.635794	1.793613	1.486602
66	6	0	-5.449774	1.442633	2.955685
67	1	0	-6.580141	2.302678	1.312062
68	1	0	-4.820746	2.422475	1.132950
69	1	0	-5.460486	2.355152	3.557797
70	1	0	-6.255826	0.790048	3.300937
71	1	0	-4.491322	0.939601	3.107303
72	16	0	-8.257568	0.408903	0.994928
73	8	0	-5.521039	-2.743542	-1.628703
74	8	0	-3.356841	0.788656	0.614316
75	7	0	1.056453	5.314799	-0.771948
76	6	0	1.385445	6.422507	0.109323
77	1	0	2.467160	6.501399	0.295911
78	1	0	1.033686	7.349444	-0.346050
79	1	0	0.874675	6.295465	1.067270
80	6	0	1.255986	5.560279	-2.192159
81	1	0	0.704595	4.821640	-2.780661
82	1	0	0.856347	6.545812	-2.436838
83	1	0	2.316512	5.522295	-2.481768

6-31G* Energies:

SCF Done: E (RM062X) = –2865.90840522 a.u.
Thermal correction to Gibbs Free Energy = 0.6134 a.u.
Single point 6-311+G(2d,p) energy: –2866.5018 a.u.

2a. Conformation A.

Standard orientation:

Center Number	Atomic Number	Atomic Type	Coordinates (Angstroms) X	Y	Z
1	6	0	-0.936588	-0.711433	3.489055
2	6	0	-0.957715	-0.737664	2.139584
3	6	0	0.000000	0.000000	1.346779
4	6	0	0.957715	0.737664	2.139584
5	6	0	0.936588	0.711433	3.489055
6	1	0	-1.750937	-1.289392	1.658763
7	1	0	1.750937	1.289392	1.658763
8	8	0	0.000000	0.000000	4.166961
9	6	0	-0.000000	-0.000000	-0.044986
10	6	0	0.770556	0.957805	-0.838515
11	6	0	1.389562	0.700733	-2.057894
12	16	0	0.913022	2.630508	-0.408108
13	6	0	1.989874	1.832079	-2.609309
14	1	0	1.405409	-0.283004	-2.511254
15	6	0	1.843118	2.991193	-1.843619
16	1	0	2.541861	1.834031	-3.543087
17	6	0	-0.770556	-0.957805	-0.838515
18	6	0	-1.389562	-0.700733	-2.057894
19	16	0	-0.913022	-2.630508	-0.408108
20	6	0	-1.989874	-1.832079	-2.609309
21	1	0	-1.405409	0.283004	-2.511254
22	6	0	-1.843118	-2.991193	-1.843619
23	1	0	-2.541861	-1.834031	-3.543087
24	6	0	2.421792	4.208576	-2.285223
25	1	0	2.926990	4.089059	-3.243257
26	6	0	-2.421792	-4.208576	-2.285223
27	1	0	-2.926990	-4.089059	-3.243257
28	6	0	-1.888648	-1.410153	4.431868
29	6	0	-2.615344	-0.347547	5.274450
30	6	0	-2.912386	-2.244053	3.658646
31	6	0	-1.071178	-2.330798	5.355449
32	1	0	-1.905105	0.253602	5.849049
33	1	0	-3.202644	0.320713	4.636767
34	1	0	-3.295302	-0.841208	5.975804
35	1	0	-2.425502	-3.006902	3.041382
36	1	0	-3.568840	-2.752742	4.370515
37	1	0	-3.536890	-1.617710	3.013588
38	1	0	-1.746966	-2.841254	6.048607
39	1	0	-0.534796	-3.088728	4.775629
40	1	0	-0.344923	-1.760020	5.940472
41	6	0	1.888648	1.410153	4.431868
42	6	0	2.615344	0.347547	5.274450
43	6	0	2.912386	2.244053	3.658646
44	6	0	1.071178	2.330798	5.355449
45	1	0	1.905105	-0.253602	5.849049
46	1	0	3.202644	-0.320713	4.636767
47	1	0	3.295302	0.841208	5.975804
48	1	0	2.425502	3.006902	3.041382
49	1	0	3.568840	2.752742	4.370515
50	1	0	3.536890	1.617710	3.013588
51	1	0	1.746966	2.841254	6.048607
52	1	0	0.534796	3.088728	4.775629
53	1	0	0.344923	1.760020	5.940472
54	6	0	-2.503785	-5.494869	-1.812667
55	6	0	-3.230404	-6.443752	-2.659771
56	6	0	-1.922167	-5.948859	-0.565400
57	7	0	-3.316463	-7.766669	-2.192223
58	7	0	-2.094317	-7.304401	-0.237397
59	6	0	-2.707641	-8.233089	-1.049052
60	6	0	2.503785	5.494869	-1.812667
61	6	0	3.230404	6.443752	-2.659771
62	6	0	1.922167	5.948859	-0.565400
63	7	0	3.316463	7.766669	-2.192223

64	7	0	2.094317	7.304401	-0.237397
65	6	0	2.707641	8.233089	-1.049052
66	8	0	1.294967	5.222505	0.191790
67	8	0	-1.294967	-5.222505	0.191790
68	8	0	3.742506	6.145823	-3.726299
69	8	0	-3.742506	-6.145823	-3.726299
70	16	0	2.712037	9.864634	-0.659955
71	16	0	-2.712037	-9.864634	-0.659955
72	6	0	1.464510	7.719113	1.033586
73	6	0	-0.000000	8.083729	0.838247
74	1	0	2.038460	8.556043	1.422904
75	1	0	1.565695	6.872192	1.710004
76	1	0	-0.437109	8.380049	1.795529
77	1	0	-0.099783	8.917657	0.138792
78	1	0	-0.557567	7.225560	0.454509
79	6	0	-1.464510	-7.719113	1.033586
80	6	0	0.000000	-8.083729	0.838247
81	1	0	-2.038460	-8.556043	1.422904
82	1	0	-1.565695	-6.872192	1.710004
83	1	0	0.437109	-8.380049	1.795529
84	1	0	0.099783	-8.917657	0.138792
85	1	0	0.557567	-7.225560	0.454509
86	6	0	4.040378	8.693646	-3.086962
87	6	0	3.122152	9.268835	-4.155481
88	1	0	4.841352	8.110694	-3.537831
89	1	0	4.473418	9.474131	-2.466634
90	1	0	3.688041	9.942638	-4.804335
91	1	0	2.705282	8.466584	-4.769642
92	1	0	2.304631	9.832644	-3.699022
93	6	0	-4.040378	-8.693646	-3.086962
94	6	0	-3.122152	-9.268835	-4.155481
95	1	0	-4.841352	-8.110694	-3.537831
96	1	0	-4.473418	-9.474131	-2.466634
97	1	0	-3.688041	-9.942638	-4.804335
98	1	0	-2.705282	-8.466584	-4.769642
99	1	0	-2.304631	-9.832644	-3.699022

6-31G* Energies:

SCF Done: E (RM062X) = –3738.89979237 a.u.
Thermal correction to Gibbs Free Energy = 0.7170 a.u.
Single point 6-311+G(2d,p) energy: –3739.6659 a.u.

2a. Conformation B.

Standard orientation:

Center Number	Atomic Number	Atomic Type	Coordinates (Angstroms) X	Y	Z
1	6	0	0.979296	-0.654720	6.015231
2	6	0	1.016356	-0.661095	4.666739
3	6	0	-0.000000	0.000000	3.875736
4	6	0	-1.016356	0.661095	4.666739
5	6	0	-0.979296	0.654720	6.015231
6	1	0	1.801462	-1.218662	4.176971
7	1	0	-1.801462	1.218662	4.176971
8	8	0	-0.000000	0.000000	6.692832
9	6	0	-0.000000	0.000000	2.488462
10	6	0	-1.173417	0.443651	1.735712
11	6	0	-2.508623	0.172244	2.006231
12	16	0	-1.003534	1.442706	0.331694
13	6	0	-3.374671	0.765936	1.084859
14	1	0	-2.827990	-0.458474	2.827543

15	6	0	-2.732739	1.498617	0.085385
16	1	0	-4.454044	0.661580	1.109787
17	6	0	1.173417	-0.443651	1.735712
18	6	0	2.508623	-0.172244	2.006231
19	16	0	1.003534	-1.442706	0.331694
20	6	0	3.374671	-0.765936	1.084859
21	1	0	2.827990	0.458474	2.827543
22	6	0	2.732739	-1.498617	0.085385
23	1	0	4.454044	-0.661580	1.109787
24	6	0	-3.505083	2.132728	-0.924958
25	1	0	-4.572312	1.972312	-0.774451
26	6	0	3.505083	-2.132728	-0.924958
27	1	0	4.572312	-1.972312	-0.774451
28	6	0	1.941248	-1.332851	6.963589
29	6	0	1.157822	-2.345722	7.816606
30	6	0	3.049506	-2.056771	6.196979
31	6	0	2.563939	-0.262122	7.876844
32	1	0	0.369591	-1.851480	8.391192
33	1	0	0.700468	-3.114779	7.186032
34	1	0	1.839936	-2.835766	8.518440
35	1	0	3.628943	-1.362922	5.579048
36	1	0	3.732733	-2.523709	6.912296
37	1	0	2.644473	-2.844402	5.553675
38	1	0	3.256554	-0.739470	8.577080
39	1	0	3.120677	0.475355	7.289822
40	1	0	1.795816	0.259610	8.454269
41	6	0	-1.941248	1.332851	6.963589
42	6	0	-1.157822	2.345722	7.816606
43	6	0	-3.049506	2.056771	6.196979
44	6	0	-2.563939	0.262122	7.876844
45	1	0	-0.369591	1.851480	8.391192
46	1	0	-0.700468	3.114779	7.186032
47	1	0	-1.839936	2.835766	8.518440
48	1	0	-3.628943	1.362922	5.579048
49	1	0	-3.732733	2.523709	6.912296
50	1	0	-2.644473	2.844402	5.553675
51	1	0	-3.256554	0.739470	8.577080
52	1	0	-3.120677	-0.475355	7.289822
53	1	0	-1.795816	-0.259610	8.454269
54	6	0	3.247229	-2.891312	-2.037371
55	6	0	4.419499	-3.334043	-2.798257
56	6	0	1.920256	-3.274545	-2.479788
57	7	0	4.169824	-4.102321	-3.947890
58	7	0	1.836986	-4.046013	-3.651117
59	6	0	2.922530	-4.523865	-4.351651
60	6	0	-3.247229	2.891312	-2.037371
61	6	0	-4.419499	3.334043	-2.798257
62	6	0	-1.920256	3.274545	-2.479788
63	7	0	-4.169824	4.102321	-3.947890
64	7	0	-1.836986	4.046013	-3.651117
65	6	0	-2.922530	4.523865	-4.351651
66	8	0	-0.897124	2.968892	-1.886459
67	8	0	0.897124	-2.968892	-1.886459
68	8	0	-5.568742	3.075418	-2.481730
69	8	0	5.568742	-3.075418	-2.481730
70	16	0	-2.732700	5.577117	-5.642628
71	16	0	2.732700	-5.577117	-5.642628
72	6	0	-0.463737	4.417434	-4.051680
73	6	0	-0.000000	5.683775	-3.346677
74	1	0	-0.460668	4.534935	-5.132204
75	1	0	0.166888	3.571636	-3.783728
76	1	0	1.018477	5.928248	-3.660034
77	1	0	-0.651172	6.525073	-3.597418
78	1	0	-0.002974	5.538693	-2.263353
79	6	0	0.463737	-4.417434	-4.051680
80	6	0	0.000000	-5.683775	-3.346677
81	1	0	0.460668	-4.534935	-5.132204
82	1	0	-0.166888	-3.571636	-3.783728
83	1	0	-1.018477	-5.928248	-3.660034
84	1	0	0.651172	-6.525073	-3.597418
85	1	0	0.002974	-5.538693	-2.263353

86	6	0	-5.379766	4.536450	-4.677156
87	6	0	-5.945155	5.826657	-4.101526
88	1	0	-6.096317	3.722869	-4.580685
89	1	0	-5.107201	4.648868	-5.723325
90	1	0	-6.842299	6.115561	-4.655541
91	1	0	-6.216875	5.687342	-3.052075
92	1	0	-5.214746	6.636149	-4.178424
93	6	0	5.379766	-4.536450	-4.677156
94	6	0	5.945155	-5.826657	-4.101526
95	1	0	6.096317	-3.722869	-4.580685
96	1	0	5.107201	-4.648868	-5.723325
97	1	0	6.842299	-6.115561	-4.655541
98	1	0	6.216875	-5.687342	-3.052075
99	1	0	5.214746	-6.636149	-4.178424

6-31G* Energies:

SCF Done: E (RM062X) = –3738.90038613 a.u.
Thermal correction to Gibbs Free Energy = 0.7171 a.u.
Single point 6-311+G(2d,p) energy: –3739.6662 a.u.

1Hb. Conformation A.

Standard orientation:

Center Number	Atomic Number	Atomic Type	Coordinates (Angstroms) X	Y	Z
1	6	0	2.884085	-2.064491	0.296755
2	6	0	2.156727	-0.930657	0.369152
3	6	0	2.734904	0.366879	0.087436
4	6	0	4.157661	0.313387	-0.192405
5	6	0	4.825195	-0.854987	-0.265265
6	1	0	1.134668	-1.024301	0.698923
7	1	0	4.694500	1.230174	-0.393034
8	6	0	6.287144	-1.062509	-0.588504
9	6	0	6.393103	-1.936100	-1.850608
10	6	0	6.990426	0.274129	-0.830903
11	6	0	6.953042	-1.785441	0.595465
12	1	0	5.911345	-2.906266	-1.700732
13	1	0	5.922865	-1.441822	-2.706719
14	1	0	7.447667	-2.108288	-2.088121
15	1	0	6.938860	0.919928	0.051724
16	1	0	8.045260	0.088498	-1.053570
17	1	0	6.554172	0.808194	-1.681075
18	1	0	8.011557	-1.952811	0.372625
19	1	0	6.883403	-1.184425	1.507795
20	1	0	6.482980	-2.755268	0.780368
21	6	0	2.404797	-3.472431	0.568675
22	6	0	2.628566	-4.315978	-0.698916
23	6	0	3.228787	-4.057316	1.728812
24	6	0	0.918959	-3.489066	0.935790
25	1	0	2.056206	-3.913295	-1.540653
26	1	0	3.686190	-4.340086	-0.975603
27	1	0	2.296305	-5.342649	-0.515966
28	1	0	3.089064	-3.468981	2.641332
29	1	0	2.903326	-5.083409	1.926821
30	1	0	4.294733	-4.075215	1.485274
31	1	0	0.606703	-4.522478	1.112227
32	1	0	0.724446	-2.918686	1.849871
33	1	0	0.297019	-3.082594	0.130810
34	8	0	4.197358	-2.044630	-0.046674
35	6	0	2.047708	1.566983	0.079190
36	6	0	0.611877	1.744436	0.171281
37	6	0	-0.034856	2.961500	0.384090
38	6	0	-1.431054	2.871101	0.359686
39	1	0	0.508910	3.883205	0.548034
40	6	0	-1.904772	1.589755	0.117476
41	1	0	-2.098033	3.711779	0.517671
42	6	0	2.814749	2.831096	-0.039541
43	6	0	2.924568	3.669423	-1.113777
44	6	0	3.730844	4.820516	-0.855441
45	1	0	2.440659	3.463881	-2.062342
46	6	0	4.218018	4.842961	0.417632
47	1	0	3.939239	5.591326	-1.587899
48	1	0	4.853473	5.586972	0.878118
49	6	0	-3.275465	1.218562	0.059214
50	1	0	-3.993876	2.020585	0.203285
51	6	0	-3.782450	-0.025592	-0.139616
52	6	0	-5.148327	-0.473541	-0.155063
53	8	0	-2.947555	-1.115812	-0.265175
54	6	0	-5.102469	-1.842912	-0.240357
55	6	0	-3.701379	-2.266834	-0.317781
56	16	0	-0.568069	0.485209	-0.083071
57	16	0	3.699357	3.467400	1.319320
58	8	0	-3.199666	-3.355350	-0.418275
59	6	0	-6.181981	-2.759439	-0.318549
60	7	0	-7.056374	-3.517828	-0.379852
61	6	0	-6.336721	0.383179	-0.084244
62	6	0	-7.419933	0.007544	0.721250

63	6	0	-6.411634	1.561883	-0.838942
64	6	0	-8.553917	0.809202	0.781632
65	1	0	-7.361452	-0.900320	1.314054
66	6	0	-7.552047	2.355638	-0.779475
67	1	0	-5.591732	1.834823	-1.496139
68	6	0	-8.621455	1.983407	0.032872
69	1	0	-9.384998	0.517863	1.415287
70	1	0	-7.607388	3.261637	-1.373793
71	1	0	-9.509332	2.605686	0.079249

6-31G* Energies:

SCF Done: E (RM062X) = –2390.33759577 a.u.
Thermal correction to Gibbs Free Energy = 0.4968 a.u.
Single point 6-311+G(2d,p) energy: –2390.8741 a.u.

1Hb. Conformation B.

Standard orientation:

Center Number	Atomic Number	Atomic Type	Coordinates (Angstroms) X	Y	Z
1	6	0	4.479796	1.937483	-0.129750
2	6	0	3.236917	1.413030	-0.138198
3	6	0	3.009249	-0.013508	-0.036180
4	6	0	4.234518	-0.790211	-0.006867
5	6	0	5.448019	-0.204577	0.014462
6	1	0	2.416735	2.098449	-0.278292
7	1	0	4.178775	-1.868737	0.044328
8	6	0	6.797164	-0.882216	0.088338
9	6	0	7.519245	-0.404105	1.360071
10	6	0	6.645301	-2.403847	0.127609
11	6	0	7.616122	-0.480051	-1.150776
12	1	0	7.655884	0.680811	1.352167
13	1	0	6.952343	-0.677639	2.255705
14	1	0	8.505624	-0.875021	1.418913
15	1	0	6.139076	-2.779278	-0.767675
16	1	0	7.637812	-2.861683	0.172576
17	1	0	6.083066	-2.727920	1.009201
18	1	0	8.601357	-0.954970	-1.107321
19	1	0	7.117039	-0.804378	-2.069549
20	1	0	7.758182	0.603259	-1.195174
21	6	0	4.851745	3.399302	-0.233154
22	6	0	5.653834	3.787275	1.021662
23	6	0	5.724769	3.597631	-1.484339
24	6	0	3.604620	4.281019	-0.333234
25	1	0	5.053489	3.647258	1.926235
26	1	0	6.563564	3.187040	1.110054
27	1	0	5.940885	4.841557	0.958318
28	1	0	5.175509	3.322203	-2.390322
29	1	0	6.015357	4.649895	-1.563706
30	1	0	6.634061	2.992532	-1.430338
31	1	0	3.912570	5.328938	-0.393290
32	1	0	3.020510	4.050648	-1.230149
33	1	0	2.957443	4.170405	0.543663
34	8	0	5.581308	1.151169	-0.022960
35	6	0	1.770913	-0.626269	0.023849
36	6	0	0.486179	0.028252	0.174321
37	6	0	0.302685	1.374209	0.489796
38	6	0	-1.038496	1.773143	0.504712
39	1	0	1.133251	2.036743	0.697905

40	6	0	-1.928508	0.755795	0.191976
41	1	0	-1.371771	2.778021	0.740737
42	6	0	1.698167	-2.105175	-0.065465
43	6	0	1.890002	-2.908599	-1.154751
44	6	0	1.769337	-4.301762	-0.860862
45	1	0	2.110352	-2.509492	-2.138799
46	6	0	1.493039	-4.537184	0.453213
47	1	0	1.879683	-5.088434	-1.597775
48	1	0	1.347120	-5.488364	0.946536
49	6	0	-3.343286	0.885739	0.153323
50	1	0	-3.738298	1.873233	0.374532
51	6	0	-4.251311	-0.088573	-0.112561
52	6	0	-5.687862	-0.035302	-0.113863
53	8	0	-3.846750	-1.387281	-0.338572
54	6	0	-6.119877	-1.325978	-0.292954
55	6	0	-4.953018	-2.200211	-0.444330
56	16	0	-1.058725	-0.723844	-0.126083
57	16	0	1.380251	-3.068642	1.350366
58	8	0	-4.860227	-3.384248	-0.634407
59	6	0	-7.450592	-1.805234	-0.397797
60	7	0	-8.533990	-2.208560	-0.481775
61	6	0	-6.505304	1.170982	0.053266
62	6	0	-7.645838	1.133107	0.866297
63	6	0	-6.172549	2.355996	-0.617213
64	6	0	-8.431392	2.269880	1.017529
65	1	0	-7.901318	0.219253	1.394175
66	6	0	-6.966879	3.487662	-0.467218
67	1	0	-5.313540	2.377559	-1.280750
68	6	0	-8.093235	3.447697	0.352428
69	1	0	-9.307428	2.236717	1.656711
70	1	0	-6.709109	4.398800	-0.996834
71	1	0	-8.710178	4.332777	0.469577

6-31G* Energies:

SCF Done: E (RM062X) = –2390.33837450 a.u.
Thermal correction to Gibbs Free Energy = 0.4972 a.u.
Single point 6-311+G(2d,p) energy: –2390.8667 a.u.

1Nb. Conformation A.

Standard orientation:

Center Number	Atomic Number	Atomic Type	Coordinates (Angstroms) X	Y	Z
1	6	0	2.038757	-2.887983	0.361571
2	6	0	1.515657	-1.644643	0.356315
3	6	0	2.292890	-0.490155	-0.044764
4	6	0	3.672999	-0.808137	-0.359203
5	6	0	4.131680	-2.075119	-0.350326
6	1	0	0.504881	-1.535526	0.715500
7	1	0	4.345486	-0.014105	-0.652480
8	6	0	5.524214	-2.552990	-0.693031
9	6	0	5.432921	-3.533360	-1.875200
10	6	0	6.429015	-1.378789	-1.070667
11	6	0	6.108777	-3.277345	0.532310
12	1	0	4.803536	-4.392673	-1.627778
13	1	0	5.016471	-3.039671	-2.759030
14	1	0	6.433848	-3.899981	-2.124261
15	1	0	6.519667	-0.661980	-0.248036
16	1	0	7.429054	-1.757011	-1.302379
17	1	0	6.053804	-0.851520	-1.953544
18	1	0	7.114933	-3.639003	0.297636
19	1	0	6.176940	-2.599700	1.389393
20	1	0	5.492023	-4.135025	0.814655
21	6	0	1.340849	-4.168158	0.761746
22	6	0	1.369670	-5.133406	-0.436479
23	6	0	2.099688	-4.793514	1.945011
24	6	0	-0.111131	-3.902948	1.166944
25	1	0	0.839106	-4.706174	-1.293311
26	1	0	2.396539	-5.357611	-0.738169
27	1	0	0.878476	-6.071658	-0.160102
28	1	0	2.096355	-4.121342	2.809077
29	1	0	1.614865	-5.730851	2.235640
30	1	0	3.137349	-5.011285	1.677191
31	1	0	-0.584159	-4.850818	1.439771
32	1	0	-0.170885	-3.236235	2.033392
33	1	0	-0.688996	-3.463160	0.346797
34	8	0	3.323362	-3.119756	-0.013425
35	6	0	1.821132	0.808659	-0.125798
36	6	0	0.436278	1.224843	-0.013348
37	6	0	0.002545	2.537534	0.172981
38	6	0	-1.389105	2.676674	0.178893
39	1	0	0.695964	3.359375	0.298464
40	6	0	-2.072345	1.483897	-0.015371
41	1	0	-1.906125	3.619088	0.325371
42	6	0	2.782241	1.914432	-0.335757
43	6	0	2.936310	2.739380	-1.408810
44	6	0	3.922318	3.758382	-1.237426
45	1	0	2.341256	2.629083	-2.309654
46	6	0	4.536295	3.699340	-0.009266
47	1	0	4.161923	4.494284	-1.993698
48	6	0	-3.485319	1.341043	-0.028756
49	1	0	-4.059185	2.252678	0.111501
50	6	0	-4.195056	0.191976	-0.178394
51	6	0	-5.614543	-0.026150	-0.141550
52	8	0	-3.553399	-1.022571	-0.296531
53	6	0	-5.795811	-1.386618	-0.190817
54	6	0	-4.485854	-2.036092	-0.295388
55	16	0	-0.939351	0.170195	-0.209108
56	16	0	3.869745	2.398030	0.954294
57	8	0	-4.171616	-3.194072	-0.378040
58	6	0	-7.012296	-2.115369	-0.211321
59	7	0	-8.000454	-2.721164	-0.224641
60	6	0	-6.644920	1.014252	-0.053470
61	6	0	-7.742351	0.835077	0.798905

62	6	0	-6.557771	2.175195	-0.833771
63	6	0	-8.728790	1.810971	0.879822
64	1	0	-7.808360	-0.059166	1.411282
65	6	0	-7.551890	3.144517	-0.753464
66	1	0	-5.730700	2.299286	-1.526073
67	6	0	-8.634874	2.966306	0.105259
68	1	0	-9.570746	1.669813	1.549377
69	1	0	-7.483560	4.036240	-1.367761
70	1	0	-9.408103	3.725254	0.167592
71	7	0	5.580511	4.467710	0.471528
72	6	0	5.654751	4.666824	1.911276
73	1	0	6.578262	5.199878	2.144069
74	1	0	4.801861	5.241088	2.302520
75	1	0	5.690500	3.700315	2.421507
76	6	0	5.900492	5.634457	-0.333312
77	1	0	5.064178	6.348186	-0.387409
78	1	0	6.767354	6.134040	0.102138
79	1	0	6.158151	5.321740	-1.348488

6-31G* Energies:

SCF Done: E (RM062X) = –2524.24246256 a.u.
Thermal correction to Gibbs Free Energy = 0.5656 a.u.
Single point 6-311+G(2d,p) energy: –2524.8190 a.u.

1Nb. Conformation B.

Standard orientation:

Center Number	Atomic Number	Atomic Type	Coordinates (Angstroms) X	Y	Z
1	6	0	-4.128671	-2.598705	-0.179696
2	6	0	-2.953220	-1.951917	-0.062279
3	6	0	-2.884516	-0.504238	0.029064
4	6	0	-4.180961	0.144115	-0.061668
5	6	0	-5.322420	-0.563904	-0.148929
6	1	0	-2.042672	-2.532625	-0.088918
7	1	0	-4.234676	1.223295	-0.025296
8	6	0	-6.735869	-0.029704	-0.203306
9	6	0	-7.519129	-0.585195	0.998757
10	6	0	-6.747213	1.499145	-0.157566
11	6	0	-7.392930	-0.507988	-1.509791
12	1	0	-7.539640	-1.678441	0.984620
13	1	0	-7.068837	-0.257506	1.941269
14	1	0	-8.550784	-0.220560	0.964343
15	1	0	-6.203451	1.929237	-1.004901
16	1	0	-7.781645	1.852223	-0.204599
17	1	0	-6.302672	1.875295	0.769319
18	1	0	-8.421668	-0.137045	-1.559706
19	1	0	-6.847598	-0.130333	-2.380721
20	1	0	-7.417836	-1.599956	-1.561737
21	6	0	-4.346919	-4.089028	-0.314423
22	6	0	-5.234167	-4.562262	0.850223
23	6	0	-5.060948	-4.363841	-1.649085
24	6	0	-3.017607	-4.845065	-0.281107
25	1	0	-4.747273	-4.373161	1.812399
26	1	0	-6.200330	-4.050355	0.842904
27	1	0	-5.413419	-5.638404	0.760122
28	1	0	-4.448647	-4.031361	-2.493471
29	1	0	-5.239923	-5.438633	-1.754795
30	1	0	-6.024218	-3.848306	-1.693746

31	1	0	-3.212346	-5.918094	-0.367780
32	1	0	-2.368121	-4.551446	-1.111941
33	1	0	-2.482460	-4.672809	0.658693
34	8	0	-5.314257	-1.927850	-0.194767
35	6	0	-1.710999	0.208695	0.156497
36	6	0	-0.432456	-0.430863	0.436671
37	6	0	-0.145186	-1.541893	1.217062
38	6	0	1.224021	-1.850609	1.259197
39	1	0	-0.903185	-2.084213	1.769409
40	6	0	2.012693	-0.979363	0.524307
41	1	0	1.646310	-2.675045	1.823935
42	6	0	-1.701661	1.670372	-0.029208
43	6	0	-2.097626	2.403985	-1.110280
44	6	0	-1.997057	3.816250	-0.939879
45	1	0	-2.462940	1.935067	-2.018537
46	6	0	-1.493610	4.164795	0.291709
47	1	0	-2.273081	4.533714	-1.701229
48	6	0	3.432003	-1.072427	0.425412
49	1	0	3.892953	-1.888596	0.974475
50	6	0	4.268693	-0.259516	-0.263735
51	6	0	5.706995	-0.289070	-0.353835
52	8	0	3.794268	0.838372	-0.948063
53	6	0	6.069395	0.830284	-1.055237
54	6	0	4.856590	1.557553	-1.450295
55	16	0	1.026122	0.242461	-0.233689
56	16	0	-1.179174	2.742208	1.261180
57	8	0	4.706998	2.565782	-2.085682
58	6	0	7.368985	1.248243	-1.442016
59	7	0	8.426180	1.600632	-1.759928
60	6	0	6.585168	-1.319385	0.210804
61	6	0	7.766002	-0.944581	0.865018
62	6	0	6.269974	-2.678277	0.075586
63	6	0	8.609649	-1.916335	1.390362
64	1	0	8.008375	0.107782	0.978400
65	6	0	7.122553	-3.645630	0.596450
66	1	0	5.377298	-2.973901	-0.467086
67	6	0	8.289293	-3.266640	1.257731
68	1	0	9.517344	-1.618882	1.904852
69	1	0	6.878346	-4.696252	0.479350
70	1	0	8.951478	-4.023491	1.665659
71	7	0	-1.189890	5.423372	0.771346
72	6	0	-1.223758	5.620994	2.212081
73	1	0	-0.862863	6.626220	2.436273
74	1	0	-2.233980	5.501673	2.631068
75	1	0	-0.553853	4.909582	2.703029
76	6	0	-1.700388	6.525190	-0.026289
77	1	0	-2.799935	6.531677	-0.075869
78	1	0	-1.357790	7.463764	0.412012
79	1	0	-1.305746	6.457603	-1.043326

6-31G* Energies:

SCF Done: E (RM062X) = –2524.24317791 a.u.
Thermal correction to Gibbs Free Energy = 0.5655 a.u.
Single point 6-311+G(2d,p) energy: –2524.8193 a.u.

2b. Conformation A.

Standard orientation:

Center Number	Atomic Number	Atomic Type	Coordinates (Angstroms) X	Y	Z
1	6	0	0.704752	-0.943364	3.747855
2	6	0	0.715023	-0.977495	2.399963
3	6	0	0.000000	-0.000000	1.604551
4	6	0	-0.715023	0.977495	2.399963
5	6	0	-0.704752	0.943364	3.747855
6	1	0	1.322978	-1.727932	1.916993
7	1	0	-1.322978	1.727932	1.916993
8	8	0	0.000000	-0.000000	4.428451
9	6	0	-1.431131	1.872324	4.693418
10	6	0	-2.413604	1.044150	5.539494
11	6	0	-0.394427	2.540488	5.613836
12	6	0	-2.197476	2.949864	3.923929
13	1	0	-3.160110	0.557266	4.903906
14	1	0	-1.888931	0.274818	6.112840
15	1	0	-2.934789	1.702068	6.242084
16	1	0	0.322440	3.128246	5.031589
17	1	0	-0.905830	3.212252	6.310329
18	1	0	0.155613	1.795468	6.195167
19	1	0	-2.694384	3.613612	4.637472
20	1	0	-1.525911	3.556993	3.307533
21	1	0	-2.966181	2.512894	3.278517
22	6	0	1.431131	-1.872324	4.693418
23	6	0	2.413604	-1.044150	5.539494
24	6	0	0.394427	-2.540488	5.613836
25	6	0	2.197476	-2.949864	3.923929
26	1	0	3.160110	-0.557266	4.903906
27	1	0	1.888931	-0.274818	6.112840
28	1	0	2.934789	-1.702068	6.242084
29	1	0	-0.322440	-3.128246	5.031589
30	1	0	0.905830	-3.212252	6.310329
31	1	0	-0.155613	-1.795468	6.195167
32	1	0	2.694384	-3.613612	4.637472
33	1	0	1.525911	-3.556993	3.307533
34	1	0	2.966181	-2.512894	3.278517
35	6	0	0.000000	-0.000000	0.219016
36	6	0	-0.478689	1.133453	-0.577266
37	6	0	-1.164073	1.072684	-1.778276
38	16	0	-0.109038	2.785322	-0.171465
39	6	0	-1.414886	2.334177	-2.344833
40	1	0	-1.474942	0.134053	-2.221504
41	6	0	-0.913857	3.385331	-1.595963
42	1	0	-1.957475	2.491250	-3.270633
43	6	0	0.478689	-1.133453	-0.577266
44	6	0	1.164073	-1.072684	-1.778276
45	16	0	0.109038	-2.785322	-0.171465
46	6	0	1.414886	-2.334177	-2.344833
47	1	0	1.474942	-0.134053	-2.221504
48	6	0	0.913857	-3.385331	-1.595963
49	1	0	1.957475	-2.491250	-3.270633
50	6	0	-1.051435	4.764677	-1.941507
51	1	0	-1.573844	4.971113	-2.871092
52	6	0	1.051435	-4.764677	-1.941507
53	1	0	1.573844	-4.971113	-2.871092
54	6	0	-0.617357	5.835600	-1.237527
55	6	0	-0.774311	7.243717	-1.516448
56	8	0	0.000000	5.682370	-0.015461
57	6	0	-0.291595	7.905636	-0.420312
58	6	0	0.214803	6.922048	0.546039
59	6	0	0.617357	-5.835600	-1.237527
60	6	0	0.774311	-7.243717	-1.516448
61	8	0	-0.000000	-5.682370	-0.015461

62	6	0	0.291595	-7.905636	-0.420312
63	6	0	-0.214803	-6.922048	0.546039
64	6	0	-1.347784	7.819974	-2.736722
65	6	0	-2.231175	8.903847	-2.646582
66	6	0	-0.995149	7.315976	-3.996176
67	6	0	-2.765740	9.463534	-3.801131
68	1	0	-2.513401	9.290027	-1.671777
69	6	0	-1.527192	7.886369	-5.147461
70	1	0	-0.279276	6.503349	-4.071859
71	6	0	-2.415434	8.955991	-5.051560
72	1	0	-3.457811	10.295472	-3.724382
73	1	0	-1.242440	7.498664	-6.119883
74	1	0	-2.831779	9.397153	-5.951397
75	6	0	1.347784	-7.819974	-2.736722
76	6	0	2.231175	-8.903847	-2.646582
77	6	0	0.995149	-7.315976	-3.996176
78	6	0	2.765740	-9.463534	-3.801131
79	1	0	2.513401	-9.290027	-1.671777
80	6	0	1.527192	-7.886369	-5.147461
81	1	0	0.279276	-6.503349	-4.071859
82	6	0	2.415434	-8.955991	-5.051560
83	1	0	3.457811	-10.295472	-3.724382
84	1	0	1.242440	-7.498664	-6.119883
85	1	0	2.831779	-9.397153	-5.951397
86	6	0	-0.192286	9.304920	-0.205066
87	7	0	-0.109025	10.445111	-0.016656
88	6	0	0.192286	-9.304920	-0.205066
89	7	0	0.109025	-10.445111	-0.016656
90	8	0	0.734152	7.058411	1.619886
91	8	0	-0.734152	-7.058411	1.619886

6-31G* Energies:

SCF Done: E (RM062X) = –3055.57297719 a.u.
Thermal correction to Gibbs Free Energy = 0.6250 a.u.
Single point 6-311+G(2d,p) energy: –3056.3048 a.u.

2b. Conformation B.

Standard orientation:

Center Number	Atomic Number	Atomic Type	Coordinates (Angstroms) X	Y	Z
1	6	0	0.493532	-1.070654	5.737697
2	6	0	0.521645	-1.096137	4.390843
3	6	0	-0.000000	0.000000	3.596782
4	6	0	-0.521645	1.096137	4.390843
5	6	0	-0.493532	1.070654	5.737697
6	1	0	0.905710	-1.978477	3.899401
7	1	0	-0.905710	1.978477	3.899401
8	8	0	-0.000000	0.000000	6.418771
9	6	0	-0.961569	2.150601	6.686651
10	6	0	-2.049898	1.562043	7.601437
11	6	0	0.234865	2.608101	7.538966
12	6	0	-1.531353	3.346059	5.921104
13	1	0	-2.909619	1.221817	7.015234
14	1	0	-1.665398	0.716835	8.178818
15	1	0	-2.392465	2.330272	8.301852
16	1	0	1.024910	3.027301	6.907702
17	1	0	-0.091484	3.380933	8.242091
18	1	0	0.651981	1.775423	8.112036

19	1	0	-1.869399	4.101404	6.636575
20	1	0	-0.776447	3.806232	5.275628
21	1	0	-2.388605	3.055612	5.304878
22	6	0	0.961569	-2.150601	6.686651
23	6	0	2.049898	-1.562043	7.601437
24	6	0	-0.234865	-2.608101	7.538966
25	6	0	1.531353	-3.346059	5.921104
26	1	0	2.909619	-1.221817	7.015234
27	1	0	1.665398	-0.716835	8.178818
28	1	0	2.392465	-2.330272	8.301852
29	1	0	-1.024910	-3.027301	6.907702
30	1	0	0.091484	-3.380933	8.242091
31	1	0	-0.651981	-1.775423	8.112036
32	1	0	1.869399	-4.101404	6.636575
33	1	0	0.776447	-3.806232	5.275628
34	1	0	2.388605	-3.055612	5.304878
35	6	0	-0.000000	0.000000	2.215629
36	6	0	-0.734225	1.013100	1.453149
37	6	0	-2.012141	1.494280	1.664659
38	16	0	-0.000000	1.792522	0.082571
39	6	0	-2.397444	2.477107	0.732411
40	1	0	-2.652361	1.122230	2.456330
41	6	0	-1.421846	2.756514	-0.207689
42	1	0	-3.367168	2.962518	0.722114
43	6	0	0.734225	-1.013100	1.453149
44	6	0	2.012141	-1.494280	1.664659
45	16	0	-0.000000	-1.792522	0.082571
46	6	0	2.397444	-2.477107	0.732411
47	1	0	2.652361	-1.122230	2.456330
48	6	0	1.421846	-2.756514	-0.207689
49	1	0	3.367168	-2.962518	0.722114
50	6	0	-1.573728	3.699138	-1.273486
51	1	0	-2.524921	4.221957	-1.313225
52	6	0	1.573728	-3.699138	-1.273486
53	1	0	2.524921	-4.221957	-1.313225
54	6	0	-0.662900	4.022875	-2.218336
55	6	0	-0.748710	5.000668	-3.279817
56	8	0	0.597955	3.468341	-2.215102
57	6	0	0.490214	5.045883	-3.856736
58	6	0	1.361945	4.065986	-3.193249
59	6	0	0.662900	-4.022875	-2.218336
60	6	0	0.748710	-5.000668	-3.279817
61	8	0	-0.597955	-3.468341	-2.215102
62	6	0	-0.490214	-5.045883	-3.856736
63	6	0	-1.361945	-4.065986	-3.193249
64	6	0	-1.938529	5.779500	-3.636577
65	6	0	-1.813852	7.142669	-3.936087
66	6	0	-3.196093	5.165081	-3.711855
67	6	0	-2.935886	7.882503	-4.290283
68	1	0	-0.842547	7.623327	-3.868763
69	6	0	-4.312918	5.909612	-4.075566
70	1	0	-3.290100	4.100556	-3.520947
71	6	0	-4.185429	7.267910	-4.360129
72	1	0	-2.834916	8.940005	-4.510410
73	1	0	-5.281858	5.426279	-4.142706
74	1	0	-5.059216	7.847124	-4.640619
75	6	0	1.938529	-5.779500	-3.636577
76	6	0	1.813852	-7.142669	-3.936087
77	6	0	3.196093	-5.165081	-3.711855
78	6	0	2.935886	-7.882503	-4.290283
79	1	0	0.842547	-7.623327	-3.868763
80	6	0	4.312918	-5.909612	-4.075566
81	1	0	3.290100	-4.100556	-3.520947
82	6	0	4.185429	-7.267910	-4.360129
83	1	0	2.834916	-8.940005	-4.510410
84	1	0	5.281858	-5.426279	-4.142706
85	1	0	5.059216	-7.847124	-4.640619
86	6	0	0.924426	5.825300	-4.960785
87	7	0	1.291339	6.458655	-5.858825
88	6	0	-0.924426	-5.825300	-4.960785
89	7	0	-1.291339	-6.458655	-5.858825

90	8	0	2.506328	3.761955	-3.389349
91	8	0	-2.506328	-3.761955	-3.389349

6-31G* Energies:

SCF Done: E (RM062X) = –3055.57456341 a.u.
Thermal correction to Gibbs Free Energy = 0.6211 a.u.
Single point 6-311+G(2d,p) energy: –3056.3059 a.u.

1Hc. Conformation A.

Standard orientation:

Center Number	Atomic Number	Atomic Type	Coordinates (Angstroms) X	Y	Z
1	6	0	-4.794303	1.339629	-0.291512
2	6	0	-4.314036	0.080140	-0.253244
3	6	0	-2.924898	-0.199747	0.047911
4	6	0	-2.154453	0.980185	0.371221
5	6	0	-2.700385	2.215339	0.334720
6	1	0	-4.980441	-0.736080	-0.494667
7	1	0	-1.133235	0.904932	0.708972
8	8	0	-3.995247	2.406430	-0.017594
9	6	0	-2.441222	-1.499209	0.025105
10	6	0	-1.063544	-1.915978	0.139740
11	6	0	-0.640097	-3.237441	0.315753
12	16	0	0.327299	-0.873400	-0.034451
13	6	0	0.747041	-3.391170	0.326611
14	1	0	-1.341347	-4.054035	0.428833
15	6	0	1.443632	-2.202289	0.150016
16	1	0	1.253122	-4.340963	0.463074
17	6	0	-3.407137	-2.614375	-0.132962
18	6	0	-3.652389	-3.385019	-1.234984
19	16	0	-4.393711	-3.134246	1.204508
20	6	0	-4.643725	-4.390355	-1.014303
21	1	0	-3.136545	-3.233039	-2.176774
22	6	0	-5.133641	-4.371287	0.257748
23	1	0	-4.976086	-5.090361	-1.771598
24	6	0	2.850394	-2.049227	0.134020
25	6	0	3.539416	-0.874924	-0.015118
26	1	0	3.404905	-2.979592	0.255376
27	1	0	3.006544	0.065832	-0.135171
28	6	0	4.950183	-0.819812	-0.018311
29	6	0	5.875043	-1.996475	0.126606
30	6	0	5.748245	0.297384	-0.152609
31	8	0	7.195468	-1.439161	0.059499
32	1	0	5.769469	-2.722266	-0.684334
33	6	0	7.125343	-0.116604	-0.100725
34	1	0	5.768391	-2.505089	1.088738
35	6	0	8.290751	0.609388	-0.187068
36	6	0	9.545694	-0.060601	-0.104628
37	7	0	10.567182	-0.604676	-0.037833
38	6	0	8.286058	2.022349	-0.358557
39	7	0	8.304021	3.173219	-0.498431
40	6	0	5.259055	1.621811	-0.312225
41	7	0	4.800555	2.678403	-0.438846
42	6	0	-6.200338	1.780449	-0.626929
43	6	0	-6.145903	2.706030	-1.854889
44	6	0	-7.093406	0.576487	-0.931895
45	6	0	-6.771521	2.552396	0.575514

46	1	0	-5.527394	3.586286	-1.659019
47	1	0	-5.737323	2.178561	-2.722696
48	1	0	-7.157371	3.043986	-2.101290
49	1	0	-7.158249	-0.101808	-0.074877
50	1	0	-8.103044	0.929378	-1.161294
51	1	0	-6.727208	0.014013	-1.796679
52	1	0	-7.787519	2.887100	0.343877
53	1	0	-6.812537	1.914758	1.464365
54	1	0	-6.163556	3.431909	0.804756
55	6	0	-2.019294	3.524281	0.663180
56	6	0	-2.761798	4.182540	1.839113
57	6	0	-0.553415	3.302326	1.041038
58	6	0	-2.097840	4.437887	-0.572858
59	1	0	-3.809005	4.372291	1.587934
60	1	0	-2.725774	3.545754	2.728734
61	1	0	-2.286695	5.138622	2.079928
62	1	0	0.005736	2.834641	0.223710
63	1	0	-0.089771	4.269123	1.256962
64	1	0	-0.458204	2.676590	1.934361
65	1	0	-1.618094	5.395345	-0.347446
66	1	0	-1.581487	3.985746	-1.425614
67	1	0	-3.136205	4.631139	-0.855777
68	1	0	-5.887611	-5.011850	0.694350

6-31G* Energies:

SCF Done: E (RM062X) = –2347.15697536 a.u.
Thermal correction to Gibbs Free Energy = 0.4629 a.u.
Single point 6-311+G(2d,p) energy: –2347.6815 a.u.

1Hc. Conformation B.

Standard orientation:

Center Number	Atomic Number	Atomic Type	Coordinates (Angstroms) X	Y	Z
1	6	0	5.613938	0.375298	0.186291
2	6	0	4.391635	0.912901	0.006225
3	6	0	3.205367	0.085738	-0.100296
4	6	0	3.460568	-1.328445	0.089434
5	6	0	4.706889	-1.798881	0.296444
6	1	0	4.300605	1.985785	-0.091967
7	1	0	2.637463	-2.026990	0.121019
8	8	0	5.784874	-0.970154	0.314258
9	6	0	1.955364	0.626884	-0.328276
10	6	0	0.782395	-0.178576	-0.612480
11	6	0	0.666686	-1.392723	-1.280549
12	16	0	-0.786084	0.389602	-0.106338
13	6	0	-0.654394	-1.856058	-1.366941
14	1	0	1.511792	-1.897515	-1.731809
15	6	0	-1.580785	-1.006719	-0.781150
16	1	0	-0.945617	-2.776111	-1.862558
17	6	0	1.757487	2.089235	-0.247805
18	6	0	1.904759	2.906794	0.840982
19	16	0	1.286917	3.021110	-1.644118
20	6	0	1.636036	4.280708	0.560645
21	1	0	2.192965	2.526782	1.815236
22	6	0	1.285787	4.488880	-0.740584
23	1	0	1.692647	5.072415	1.298068
24	6	0	-2.986905	-1.213602	-0.744450
25	6	0	-3.914315	-0.379420	-0.189840

26	1	0	-3.317017	-2.141007	-1.212140
27	1	0	-3.609354	0.549141	0.287620
28	6	0	-5.299915	-0.673695	-0.205817
29	6	0	-5.926247	-1.897290	-0.813611
30	6	0	-6.324543	0.080777	0.317416
31	8	0	-7.333599	-1.745100	-0.580493
32	1	0	-5.598741	-2.824391	-0.335044
33	6	0	-7.568333	-0.605888	0.071354
34	1	0	-5.764425	-1.965380	-1.892895
35	6	0	-8.861503	-0.269856	0.393000
36	6	0	-9.928196	-1.140576	0.024133
37	7	0	-10.796243	-1.847686	-0.275804
38	6	0	-9.176427	0.931225	1.089907
39	7	0	-9.454147	1.903440	1.657026
40	6	0	-6.153540	1.324183	0.985254
41	7	0	-5.954954	2.332031	1.520631
42	6	0	6.937125	1.101745	0.267693
43	6	0	7.846577	0.594449	-0.864956
44	6	0	6.742869	2.612701	0.129532
45	6	0	7.586281	0.789689	1.627350
46	1	0	8.016875	-0.482527	-0.781923
47	1	0	7.402823	0.803009	-1.843718
48	1	0	8.815064	1.101583	-0.810174
49	1	0	6.105634	3.009350	0.926505
50	1	0	7.716554	3.106664	0.197455
51	1	0	6.298014	2.873157	-0.836162
52	1	0	8.550794	1.302434	1.697710
53	1	0	6.951874	1.134108	2.450368
54	1	0	7.757912	-0.283796	1.745176
55	6	0	5.118053	-3.233400	0.539769
56	6	0	5.786446	-3.324811	1.922542
57	6	0	3.907865	-4.167855	0.493275
58	6	0	6.125895	-3.645612	-0.547464
59	1	0	6.667223	-2.679118	1.977909
60	1	0	5.088235	-3.030917	2.712784
61	1	0	6.103058	-4.356075	2.107917
62	1	0	3.408416	-4.128520	-0.480489
63	1	0	4.242511	-5.195838	0.660314
64	1	0	3.178648	-3.920042	1.271225
65	1	0	6.442620	-4.679476	-0.377714
66	1	0	5.673558	-3.582443	-1.542391
67	1	0	7.012548	-3.006143	-0.526514
68	1	0	1.023694	5.421214	-1.221794

6-31G* Energies:

SCF Done: E (RM062X) = –2347.15801464 a.u.
Thermal correction to Gibbs Free Energy = 0.4634 a.u.
Single point 6-311+G(2d,p) energy: –2347.6817 a.u.

2c. Conformation A.

Standard orientation:

Center Number	Atomic Number	Atomic Type	Coordinates (Angstroms) X	Y	Z
1	6	0	0.753076	0.905098	3.425533
2	6	0	0.763325	0.939976	2.076326
3	6	0	0.000000	0.000000	1.284936
4	6	0	-0.763325	-0.939976	2.076326
5	6	0	-0.753076	-0.905098	3.425533
6	1	0	1.410254	1.656616	1.592405
7	1	0	-1.410254	-1.656616	1.592405
8	8	0	0.000000	0.000000	4.102579
9	6	0	-0.000000	-0.000000	-0.103773
10	6	0	-0.514085	-1.115849	-0.897941
11	6	0	-1.185318	-1.036926	-2.107400
12	16	0	-0.203237	-2.780660	-0.487552
13	6	0	-1.471257	-2.289973	-2.674886
14	1	0	-1.461181	-0.089183	-2.554118
15	6	0	-1.013966	-3.354224	-1.917097
16	1	0	-2.010417	-2.430191	-3.605449
17	6	0	0.514085	1.115849	-0.897941
18	6	0	1.185318	1.036926	-2.107400
19	16	0	0.203237	2.780660	-0.487552
20	6	0	1.471257	2.289973	-2.674886
21	1	0	1.461181	0.089183	-2.554118
22	6	0	1.013966	3.354224	-1.917097
23	1	0	2.010417	2.430191	-3.605449
24	6	0	-1.180714	-4.735914	-2.230616
25	6	0	-0.780268	-5.789696	-1.466692
26	1	0	-1.679283	-4.923086	-3.181186
27	1	0	-0.288111	-5.627109	-0.510288
28	6	0	1.180714	4.735914	-2.230616
29	6	0	0.780268	5.789696	-1.466692
30	1	0	1.679283	4.923086	-3.181186
31	1	0	0.288111	5.627109	-0.510288
32	6	0	-0.981706	-7.137341	-1.867875
33	6	0	-1.622770	-7.581492	-3.152175
34	6	0	-0.634513	-8.273185	-1.179248
35	8	0	-1.593699	-9.014797	-3.100078
36	1	0	-1.066748	-7.260047	-4.037201
37	6	0	-1.024609	-9.420348	-1.965226
38	6	0	0.981706	7.137341	-1.867875
39	6	0	1.622770	7.581492	-3.152175
40	6	0	0.634513	8.273185	-1.179248
41	8	0	1.593699	9.014797	-3.100078
42	1	0	1.066748	7.260047	-4.037201
43	6	0	1.024609	9.420348	-1.965226
44	1	0	-2.665510	-7.263815	-3.239021
45	1	0	2.665510	7.263815	-3.239021
46	6	0	-0.896376	-10.764580	-1.717214
47	6	0	0.896376	10.764580	-1.717214
48	6	0	-1.372658	-11.706471	-2.675960
49	7	0	-1.759752	-12.472158	-3.455004
50	6	0	1.372658	11.706471	-2.675960
51	7	0	1.759752	12.472158	-3.455004
52	6	0	-0.296903	-11.255904	-0.522351
53	7	0	0.187608	-11.676351	0.443070
54	6	0	0.296903	11.255904	-0.522351
55	7	0	-0.187608	11.676351	0.443070
56	6	0	0.000000	-8.285428	0.093665
57	7	0	0.515432	-8.233950	1.129515
58	6	0	-0.000000	8.285428	0.093665
59	7	0	-0.515432	8.233950	1.129515
60	6	0	1.527662	1.792945	4.371901
61	6	0	2.456045	0.911629	5.225442

62	6	0	2.359377	2.821012	3.602737
63	6	0	0.526053	2.522950	5.284408
64	1	0	1.885045	0.177254	5.800205
65	1	0	3.175729	0.379092	4.595617
66	1	0	3.011595	1.541567	5.927154
67	1	0	1.727301	3.466066	2.983303
68	1	0	2.892333	3.455367	4.316832
69	1	0	3.102874	2.337543	2.960989
70	1	0	1.072198	3.166241	5.981238
71	1	0	-0.152572	3.149212	4.696577
72	1	0	-0.069026	1.813284	5.865557
73	6	0	-1.527662	-1.792945	4.371901
74	6	0	-2.456045	-0.911629	5.225442
75	6	0	-2.359377	-2.821012	3.602737
76	6	0	-0.526053	-2.522950	5.284408
77	1	0	-1.885045	-0.177254	5.800205
78	1	0	-3.175729	-0.379092	4.595617
79	1	0	-3.011595	-1.541567	5.927154
80	1	0	-1.727301	-3.466066	2.983303
81	1	0	-2.892333	-3.455367	4.316832
82	1	0	-3.102874	-2.337543	2.960989
83	1	0	-1.072198	-3.166241	5.981238
84	1	0	0.152572	-3.149212	4.696577
85	1	0	0.069026	-1.813284	5.865557

6-31G* Energies:

SCF Done: E (RM062X) = –2969.21063172 a.u.
Thermal correction to Gibbs Free Energy = 0.5550 a.u.
Single point 6-311+G(2d,p) energy: –2969.9177 a.u.

2c. Conformation B.

Standard orientation:

Center Number	Atomic Number	Atomic Type	Coordinates (Angstroms) X	Y	Z
1	6	0	0.945994	-0.702697	6.159239
2	6	0	0.983114	-0.711035	4.811276
3	6	0	0.000000	0.000000	4.020035
4	6	0	-0.983114	0.711035	4.811276
5	6	0	-0.945994	0.702697	6.159239
6	1	0	1.742202	-1.302782	4.320315
7	1	0	-1.742202	1.302782	4.320315
8	8	0	0.000000	0.000000	6.837494
9	6	0	0.000000	0.000000	2.635517
10	6	0	-1.137385	0.520698	1.877185
11	6	0	-2.487852	0.324696	2.101973
12	16	0	-0.889200	1.546144	0.491926
13	6	0	-3.310712	0.987069	1.170924
14	1	0	-2.858736	-0.304428	2.902868
15	6	0	-2.600994	1.693605	0.217236
16	1	0	-4.394238	0.940449	1.171756
17	6	0	1.137385	-0.520698	1.877185
18	6	0	2.487852	-0.324696	2.101973
19	16	0	0.889200	-1.546144	0.491926
20	6	0	3.310712	-0.987069	1.170924
21	1	0	2.858736	0.304428	2.902868
22	6	0	2.600994	-1.693605	0.217236
23	1	0	4.394238	-0.940449	1.171756
24	6	0	-3.170104	2.436832	-0.862835
25	6	0	-2.483458	3.128351	-1.811577

26	1	0	-4.259282	2.425357	-0.890795
27	1	0	-1.396106	3.157925	-1.799915
28	6	0	3.170104	-2.436832	-0.862835
29	6	0	2.483458	-3.128351	-1.811577
30	1	0	4.259282	-2.425357	-0.890795
31	1	0	1.396106	-3.157925	-1.799915
32	6	0	-3.137170	3.840053	-2.855186
33	6	0	-4.623103	3.923548	-3.059289
34	6	0	-2.552246	4.571737	-3.857040
35	8	0	-4.787177	4.740955	-4.226645
36	1	0	-5.139695	4.408939	-2.226607
37	6	0	-3.597259	5.118070	-4.692721
38	6	0	3.137170	-3.840053	-2.855186
39	6	0	4.623103	-3.923548	-3.059289
40	6	0	2.552246	-4.571737	-3.857040
41	8	0	4.787177	-4.740955	-4.226645
42	1	0	5.139695	-4.408939	-2.226607
43	6	0	3.597259	-5.118070	-4.692721
44	1	0	-5.079923	2.949748	-3.256269
45	1	0	5.079923	-2.949748	-3.256269
46	6	0	-3.529259	5.909619	-5.811886
47	6	0	3.529259	-5.909619	-5.811886
48	6	0	-4.732056	6.317433	-6.460391
49	7	0	-5.709369	6.648984	-6.987468
50	6	0	4.732056	-6.317433	-6.460391
51	7	0	5.709369	-6.648984	-6.987468
52	6	0	-2.285031	6.342505	-6.353727
53	7	0	-1.283084	6.705448	-6.809576
54	6	0	2.285031	-6.342505	-6.353727
55	7	0	1.283084	-6.705448	-6.809576
56	6	0	-1.149814	4.747825	-4.018889
57	7	0	-0.000000	4.857965	-4.101417
58	6	0	1.149814	-4.747825	-4.018889
59	7	0	0.000000	-4.857965	-4.101417
60	6	0	1.873613	-1.426554	7.108227
61	6	0	1.041788	-2.401683	7.959437
62	6	0	2.946834	-2.202393	6.342656
63	6	0	2.545833	-0.387809	8.023173
64	1	0	0.276089	-1.871281	8.532238
65	1	0	0.550419	-3.148500	7.327689
66	1	0	1.698587	-2.923178	8.662752
67	1	0	3.560664	-1.536610	5.727059
68	1	0	3.605008	-2.703260	7.058535
69	1	0	2.505172	-2.968276	5.697201
70	1	0	3.215368	-0.898075	8.722617
71	1	0	3.136464	0.323797	7.437332
72	1	0	1.802967	0.168339	8.601449
73	6	0	-1.873613	1.426554	7.108227
74	6	0	-1.041788	2.401683	7.959437
75	6	0	-2.946834	2.202393	6.342656
76	6	0	-2.545833	0.387809	8.023173
77	1	0	-0.276089	1.871281	8.532238
78	1	0	-0.550419	3.148500	7.327689
79	1	0	-1.698587	2.923178	8.662752
80	1	0	-3.560664	1.536610	5.727059
81	1	0	-3.605008	2.703260	7.058535
82	1	0	-2.505172	2.968276	5.697201
83	1	0	-3.215368	0.898075	8.722617
84	1	0	-3.136464	-0.323797	7.437332
85	1	0	-1.802967	-0.168339	8.601449

6-31G* Energies:

SCF Done: E (RM062X) = –2969.21226334 a.u.
Thermal correction to Gibbs Free Energy = 0.5557 a.u.
Single point 6-311+G(2d,p) energy: –2969.9189 a.u.

6b. Conformation A.

Standard orientation:

Center Number	Atomic Number	Atomic Type	Coordinates (Angstroms) X	Y	Z
1	6	0	3.781501	1.000429	0.029243
2	6	0	2.813308	0.062414	-0.023748
3	6	0	3.132106	-1.349637	-0.075367
4	6	0	4.555904	-1.628474	-0.075171
5	6	0	5.474116	-0.645598	-0.019232
6	1	0	1.791882	0.408363	-0.031493
7	1	0	4.887484	-2.658796	-0.118276
8	6	0	6.979424	-0.779675	-0.007190
9	6	0	7.519344	-0.148714	1.288038
10	6	0	7.398670	-2.249213	-0.072656
11	6	0	7.550569	-0.028462	-1.222404
12	1	0	7.248246	0.908557	1.354418
13	1	0	7.123165	-0.666100	2.167680
14	1	0	8.611120	-0.225973	1.304847
15	1	0	7.041956	-2.726914	-0.990782
16	1	0	8.490810	-2.311306	-0.061667
17	1	0	7.019219	-2.812702	0.785735
18	1	0	8.642257	-0.108718	-1.221603
19	1	0	7.173899	-0.456359	-2.156986
20	1	0	7.283646	1.031487	-1.192243
21	6	0	3.594492	2.499900	0.086868
22	6	0	4.245823	3.024830	1.378327
23	6	0	4.290340	3.128028	-1.133220
24	6	0	2.109947	2.871938	0.075832
25	1	0	3.769380	2.586122	2.260908
26	1	0	5.313430	2.789894	1.405280
27	1	0	4.130563	4.112084	1.428928
28	1	0	3.847309	2.762682	-2.065209
29	1	0	4.173808	4.215812	-1.099442
30	1	0	5.358898	2.896085	-1.140668
31	1	0	2.013792	3.960650	0.120153
32	1	0	1.615032	2.526866	-0.837874
33	1	0	1.582770	2.451557	0.938545
34	8	0	5.098018	0.665745	0.036911
35	6	0	2.238294	-2.389235	-0.123982
36	6	0	0.807283	-2.386562	-0.124521
37	6	0	0.028040	-3.542961	-0.197374
38	6	0	-1.346435	-3.296639	-0.178407
39	1	0	0.472185	-4.529287	-0.262459
40	6	0	-1.672479	-1.948202	-0.089150
41	1	0	-2.106837	-4.068727	-0.227194
42	6	0	-2.991009	-1.428161	-0.052364
43	1	0	-3.792981	-2.158935	-0.106807
44	6	0	-3.362225	-0.122312	0.028774
45	6	0	-4.671053	0.465439	0.019766
46	8	0	-2.415215	0.880154	0.038058
47	6	0	-4.481762	1.826039	-0.022809
48	6	0	-3.044165	2.105045	-0.003139
49	16	0	-0.216195	-0.981285	-0.029249
50	8	0	-2.429023	3.139209	-0.007268
51	6	0	-5.458362	2.853890	-0.014578
52	7	0	-6.247791	3.702821	-0.011566
53	6	0	-5.944510	-0.262590	0.042828
54	6	0	-6.997068	0.148754	-0.785513
55	6	0	-6.129721	-1.349632	0.907859
56	6	0	-8.210641	-0.528554	-0.758626
57	1	0	-6.853904	0.984972	-1.463261
58	6	0	-7.348710	-2.018692	0.934915
59	1	0	-5.330917	-1.645369	1.580952
60	6	0	-8.388049	-1.612548	0.100101
61	1	0	-9.018044	-0.210811	-1.409980

62	1	0	-7.488415	-2.853169	1.614116
63	1	0	-9.337638	-2.137471	0.121762
64	1	0	2.663503	-3.389607	-0.168642

6-31G* Energies:

SCF Done: E (RM062X) = –1838.62055736 a.u.
Thermal correction to Gibbs Free Energy = 0.4553 a.u.
Single point 6-311+G(2d,p) energy: –1839.0813 a.u.

6b. Conformation B.

Standard orientation:

Center Number	Atomic Number	Atomic Type	Coordinates (Angstroms) X	Y	Z
1	6	0	4.870038	1.166805	0.327693
2	6	0	3.578883	0.812643	0.175091
3	6	0	3.209799	-0.527100	-0.241318
4	6	0	4.337150	-1.429974	-0.388619
5	6	0	5.605742	-1.007030	-0.239805
6	1	0	2.814613	1.535719	0.418774
7	1	0	4.150113	-2.464341	-0.650587
8	6	0	6.876680	-1.809541	-0.398678
9	6	0	7.655348	-1.768565	0.927569
10	6	0	6.565364	-3.262122	-0.762811
11	6	0	7.724594	-1.171511	-1.512768
12	1	0	7.911557	-0.741710	1.202647
13	1	0	7.067065	-2.210164	1.738384
14	1	0	8.583849	-2.339214	0.824748
15	1	0	6.022342	-3.329881	-1.710910
16	1	0	7.503767	-3.814058	-0.870469
17	1	0	5.972182	-3.751832	0.016100
18	1	0	8.657236	-1.732637	-1.629624
19	1	0	7.189351	-1.189399	-2.467574
20	1	0	7.974565	-0.134264	-1.273192
21	6	0	5.408315	2.509388	0.768571
22	6	0	6.221190	2.313525	2.059931
23	6	0	6.325657	3.057157	-0.338721
24	6	0	4.272144	3.500814	1.025243
25	1	0	5.589155	1.918261	2.861660
26	1	0	7.053655	1.622809	1.900107
27	1	0	6.629262	3.275791	2.385116
28	1	0	5.768935	3.199483	-1.270580
29	1	0	6.733483	4.024746	-0.029454
30	1	0	7.160062	2.377441	-0.531947
31	1	0	4.696840	4.461054	1.332405
32	1	0	3.674904	3.668066	0.122785
33	1	0	3.609470	3.153787	1.824532
34	8	0	5.883219	0.289837	0.086667
35	6	0	1.935259	-0.976461	-0.455357
36	6	0	0.723131	-0.201404	-0.444964
37	6	0	0.495060	1.154225	-0.663624
38	6	0	-0.862359	1.502346	-0.609436
39	1	0	1.278103	1.856863	-0.916665
40	6	0	-1.709504	0.432938	-0.358436
41	1	0	-1.238105	2.507372	-0.769748
42	6	0	-3.126145	0.529965	-0.277586
43	1	0	-3.541869	1.525122	-0.407690
44	6	0	-4.012639	-0.470155	-0.044254

45	6	0	-5.444387	-0.414293	0.085256
46	8	0	-3.584378	-1.756685	0.205219
47	6	0	-5.844771	-1.670998	0.461297
48	6	0	-4.667353	-2.542294	0.534299
49	16	0	-0.782566	-1.035216	-0.176639
50	8	0	-4.555242	-3.708977	0.801278
51	6	0	-7.161380	-2.147916	0.687049
52	7	0	-8.232616	-2.547629	0.876754
53	6	0	-6.284238	0.766549	-0.142362
54	6	0	-7.334867	1.055745	0.738395
55	6	0	-6.064796	1.593081	-1.252847
56	6	0	-8.142281	2.165776	0.518594
57	1	0	-7.502239	0.422088	1.604226
58	6	0	-6.880959	2.697814	-1.470943
59	1	0	-5.278805	1.348727	-1.960700
60	6	0	-7.916437	2.988003	-0.584597
61	1	0	-8.947613	2.389876	1.210209
62	1	0	-6.711650	3.327852	-2.337865
63	1	0	-8.550454	3.852027	-0.755621
64	1	0	1.817380	-2.040570	-0.646605

6-31G* Energies:

SCF Done: E (RM062X) = –1838.61837674 a.u.
Thermal correction to Gibbs Free Energy = 0.4564 a.u.
Single point 6-311+G(2d,p) energy: –1839.0789a.u.
